# Completing the series of boron-nucleophilic cyanoborates: boryl anions of type NHC–B(CN)_2_^–^ †
†Dedicated to Prof. Dr Evamarie Hey-Hawkins on the occasion of her 60^th^ birthday.
‡
‡Electronic supplementary information (ESI) available: Experimental procedures, full characterisation, crystallographic data and computational details. CCDC 1550754 (**1B**), 1550755 (**2B**), 1550760 (**7B**), 1550767 (**K-8B**), 1550769 (**9**), 1550740 (**1C**), 1550756 (**2C**), 1550774 (**7C**), 1550768 {**[K(18-cr-6)]-8C**}, 1550770 (**11**), 1550771 (**13**), 1550772 (**14**), 1550757 (**3A**), 1550759 (**7A**), 1550758 (**4**). For ESI and crystallographic data in CIF or other electronic format see DOI: 10.1039/c7sc02238g



**DOI:** 10.1039/c7sc02238g

**Published:** 2017-07-03

**Authors:** Richard Böser, Lisa C. Haufe, Matthias Freytag, Peter G. Jones, Gerald Hörner, René Frank

**Affiliations:** a Technical University of Braunschweig , Department of Life Sciences , Institute of Analytical and Inorganic Chemistry , Hagenring 30 , 38106 , Braunschweig , Germany . Email: r.frank@tu-braunschweig.de; b Technical University of Berlin , Department of Chemistry , Institute of Bioinorganic Chemistry , Strasse des 17. Juni 135 , 10623 Berlin , Germany . Email: gerald.hoerner@tu-berlin.de

## Abstract

Since the first seminal report of boron-centred nucleophiles, the area of boryl anions has developed only sporadically and requires further systematisation. The boryl anions of type NHC–B(CN)_2_^–^ (NHC = N-heterocyclic carbene) described herein complete a consistent series with the known anions cAAC–B(CN)_2_^–^ [cAAC = cyclic(alkyl)(amino)carbene] and B(CN)_3_^2–^. A novel approach towards NHC-stabilised cyanoboranes based on alkylthio-cyano exchange at boron is presented, and in contrast to other methods affords the products in better purity and yield. Reduction of suitable NHC–dicyanoboranes gave two unprecedented examples of NHC–B(CN)_2_^–^ boryl anions. The latter were shown to react as boron-centred nucleophiles with facile formation of B–E bonds, where E = C, Si, Sn, P, Au. Bonding analysis by DFT calculations suggests a systematic variation of the energy of the boron-centred HOMO depending on the carbene, which in turn can control the nucleophilic character.

## Abstract 2

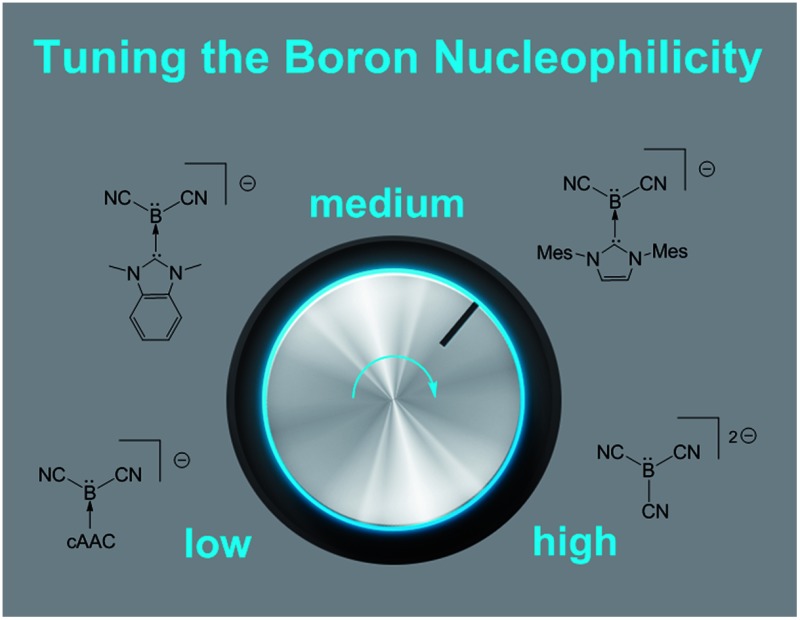
The novel boryl anions NHC–B(CN)_2_^–^ complete a series of cyanoborates with continuously increasing boron-centred nucleophilicity.

## Introduction

**Scheme 1:**
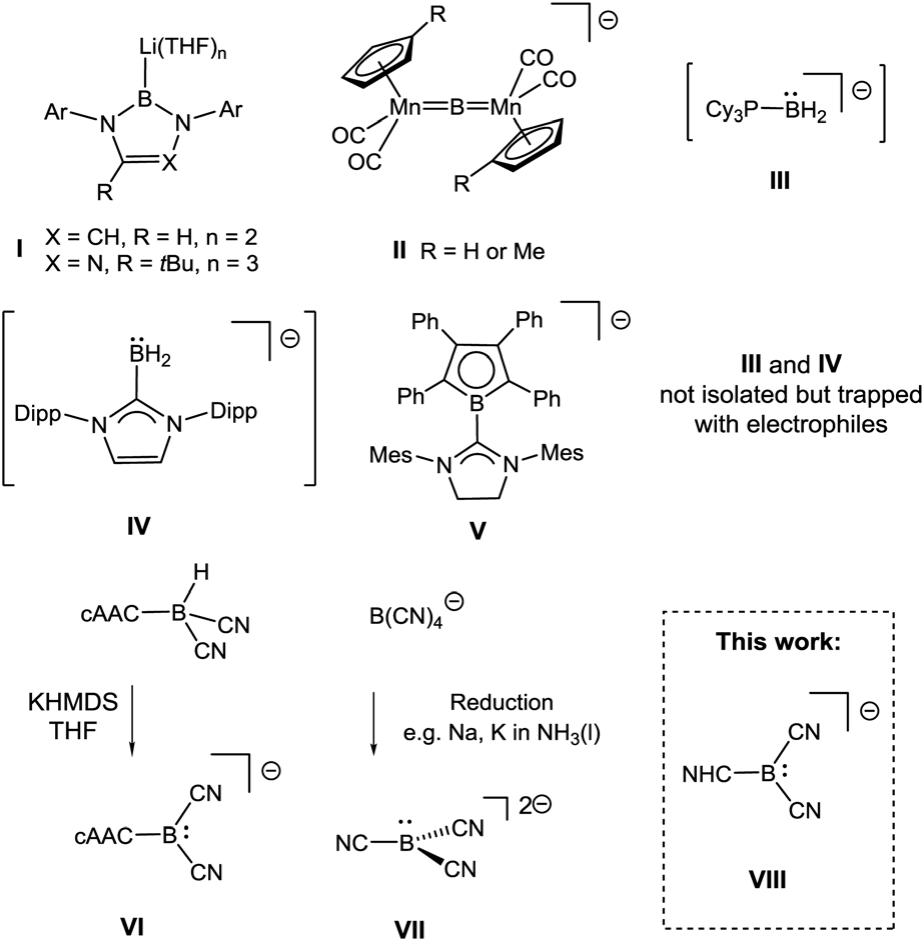
Anionic species with boron-centred nucleophilicity. Cations omitted. For rare examples of neutral boron nucleophiles see ref. 5, Ar = aryl, Mes = 2,4,6-Me_3_C_6_H_2_, Dipp = 2,6-(iPr)_2_C_6_H_3_.

The electron-deficient nature of boron has limited the reactivity of mononuclear boron centres to Lewis-acidity. This is obvious for compounds of type BR_3_ with a vacant p-orbital but also holds for four-coordinate borates BR_4_^–^. Although the latter serve as transfer reagents of the nucleophile R^–^, the boron centre displays Lewis-acidic properties to stabilise the substituent R^–^, with the most prominent example being BH_4_^–^ as a common reducing agent.^1^ Boron-centred nucleophiles were long ago considered as attractive alternatives to classical boron reagents; attempts to target them date back 50 years, but include erroneous reports.^2^ For example, the preparation of nucleophilic boryl anions of type BR_2_^–^, R = *n*-Bu, Ph, was claimed in at least two cases but was unambiguously refuted later.^3^ The discovery of the first (structurally confirmed) anions with boron-centred nucleophilicity (**I**, X = CH, R = H, Scheme 1) in 2006 therefore represented a significant breakthrough.^4^

These boron nucleophiles opened up routes to species such as boryl complexes of electropositive metals or metalloids,^6^ which are difficult to obtain with traditional electrophilic boron reagents. Most remarkably, the stronger electron-releasing character of boryl anions **I** compared to carbanions has led to the isolation of species for which no organometallic precedents are known.^7^ Recent examples documenting this behaviour include stable radicals ˙MR_2_, M = Ga, In, Tl^8^ or a germanium analogue of vinylidene Ge=GeR_2_,^9^ and these results have stimulated the quest for further anionic boryl species. Subsequently, a triazaborol-3-yl anion (**I**, X = N, R = *t*Bu, Scheme 1) was prepared,^10^ and nucleophilic behaviour at the bridging boron atom was found in an anionic dimanganese borylene complex **II**.^11^ Attempts to stabilise the proposed six-electron species of type BR_2_^–^ involved the use of strong σ-donor ligands L; the parent anionic species L–BH_2_^–^, **III** (L = PCy_3_)^12^ and **IV** (L = IDipp)^13^ were obtained, although isolation or crystallisation of pure material was impossible because of instability at ambient temperature.

In contrast, the NHC-substituted π-borolyl anion **V** displayed better stability, but its behaviour as a true nucleophile was called into question because of strong evidence of radical pathways.^14^ Cyano moieties facilitate boron-centred nucleophilicity since the π-acidic character stabilises p_*z*_-located lone pairs. Thus, the boryl anion **VI** cAAC–B(CN)_2_^–^ [cAAC = cyclic (alkyl)(amino)carbene] was obtained in a remarkable deprotonation reaction from the parent hydroborane cAAC–BH(CN)_2_.^15^ Tricyanoborate anion **VII** B(CN)_3_^2–^ was obtained by the reduction of B(CN)_4_^–^ or BF(CN)_3_^–^ or by deprotonation of BH(CN)_3_^–^.^16,17^ Both **VI** and **VII** react as boron-centred nucleophiles, although steric congestion caused by the cAAC-moiety strongly means that **VI** only reacts with small electrophiles. N-Heterocyclic carbenes (NHCs) behave as strong σ-donor (but also as weak acceptor) ligands^18^ and, considering their ability to stabilise main group elements, it is surprising that boryl anions of type NHC–B(CN)_2_^–^ **VIII** are unknown. We therefore set out to develop routes to anions **VIII** with various the NHC moieties, with the intention of studying the nucleophilic behaviour of such species.

## Results and discussion

**Scheme 2:**
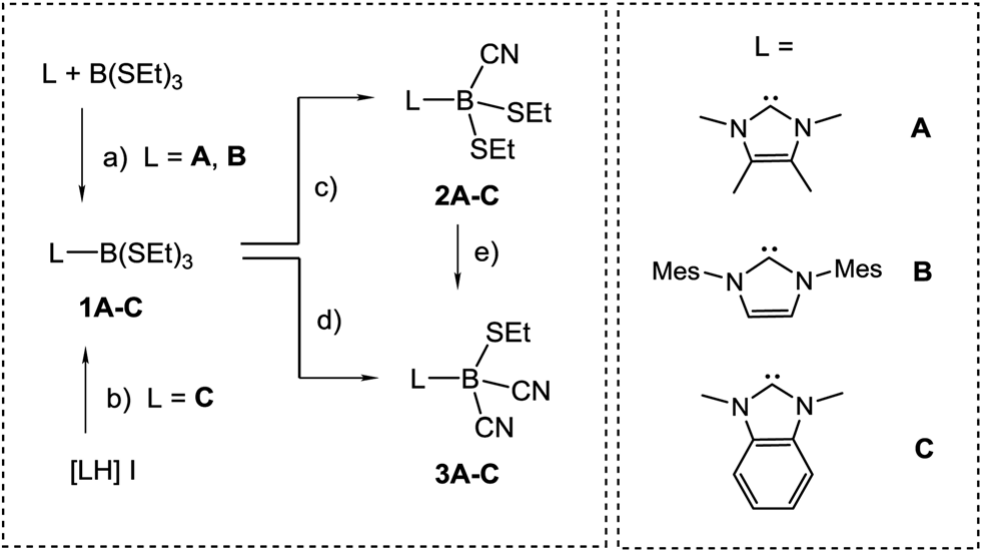
Reagents and conditions. (a) Toluene, rt, 5 min; (b) Na[N(SiMe_3_)_2_], THF, –78 °C to rt, 72 h; (c) 1 eq. Me_3_SiCN, toluene, 45 °C, 24 h; (d) 2.3 eq. Me_3_SiCN, cat. B(SEt)_3_, toluene, 95 °C, 24 h; (e) 1.3 eq. Me_3_SiCN, toluene, 95 °C, 24 h.

The preparation of anions **VI** and **VII** by deprotonation of the parent hydroboranes by strong bases prompted us to study analogous reactions with IMes–BH(CN)_2_ [IMes = *cyclo*-C{N(Mes)CH}_2_, Mes = 2,4,6-Me_3_C_6_H_2_]. However, the use of various bases of hydride, hydrocarbyl or amide character in THF or 1,4-dioxane proved unsuccessful in our hands; no deprotonation was observed and the starting material was recovered unchanged. While the stronger π-acidity of cAAC-type compared to NHC-type carbenes^19^ better stabilises the anion **VI**, the deprotonation of BH(CN)_3_^–^ is driven by the poor solubility of the alkali metal salts of **VII** in organic solvents.^17^ We interpret the ineffective deprotonation of IMes–BH(CN)_2_ as an absence of such conditions. An alternative approach was then chosen, which involves borane precursors of type NHC–BX(CN)_2_ where X is a reducible leaving group such as halide. The introduction of cyano groups into carbene–borane adducts has already been accomplished by nucleophilic replacement of boron-bound triflate moieties by cyanide, *i.e.* B–OTf → B–CN.^20^ However, the respective mono- or dicyanoborane adducts are obtained as impure samples containing *ca.* 20% of inseparable isonitrile boranes with B–NC moieties. For an improved synthetic protocol towards mono- or dicyanoboranes, we first established the NHC–borane adducts L–B(SEt)_3_ (**1A–C**, Scheme 2), in which electronic and steric properties of the NHC–moiety L (**A–C**) vary widely. For isolable N-heterocyclic carbenes **A** and **B** the adduct formation proceeds in reactions with B(SEt)_3_ to afford **1A** or **1B**, respectively. Carbene **C** is prone to rapid dimerization but can be made *in situ* by deprotonation of the imidazolium iodide [LH]I, L = **C**, and is then trapped by B(SEt)_3_ to give **1C**.^21^ Evidence for the formation of **1A–C** is provided by the high-field shift by *ca.* 60 ppm and the significant signal narrowing in the ^11^B{^1^H}-NMR spectra, *e.g. δ*(^11^B) = 60.2 ppm, *ω*_1/2_ = 72 Hz for B(SEt)_3_ *vs. δ*(^11^B) = –2.0 ppm, *ω*_1/2_ = 3 Hz for **1B**, which is expected for a shift from three- to four-coordinated boron centres. Structural authentication by X-ray crystallography is provided for compounds **1B** and **1C** (section ESI‡) and shows the expected bond geometries for this class of compounds.

**Scheme 3:**
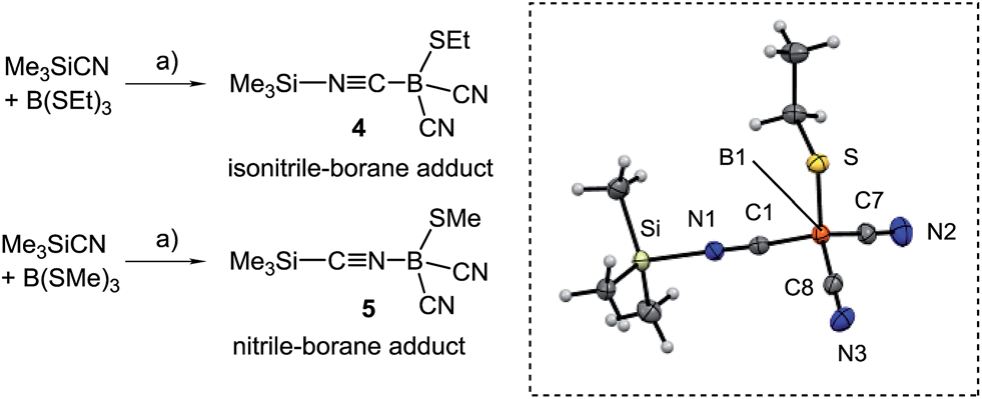
Reagents and conditions. (a) Toluene, rt, 30 min. Dashed box: molecular structure of isonitrile–borane adduct **4**. Thermal ellipsoids are presented at 50% probability levels. Selected bond lengths (Å) and angles (°). Si–N1 1.836(1), N1–C1 1.141(2), S–B1 1.898(2), B1–C8 1.588(2), B1–C7 1.588(2), B1–C1 1.608(2), N2–C7 1.144(2), N3–C8 1.146(2), C1–N1–Si 177.54(10), N1–C1–B1 176.10(12).

For the introduction of nitrile groups, we found Me_3_SiCN to be an excellent cyanation agent. Thus, the reaction of **1A–C** with Me_3_SiCN (1 eq.) in toluene neatly afforded the monocyanoboranes **2A–C**. Although carbon bound alkylthio moieties are commonly poor leaving groups, their nucleophilic replacement has been observed in boron chemistry before.^22^ In the ^11^B{^1^H}-NMR spectra the monocyanoboranes **2A–C** are high-field shifted in comparison to the starting material, *e.g. δ*(^11^B) = –13.7 ppm, *ω*_1/2_ = 3 Hz for **2B**. The IR-spectra display typical bands for the C≡N stretch vibration, *e.g.* at 2194 cm^–1^ for **2B**. X-ray crystallography confirmed the constitution of compounds **2B** and **2C** and again showed bond lengths and angles in the expected range (section ESI‡). Attempts to obtain dicyanoboranes **3A–C** were performed with analytically pure samples of **1A–C** or **2A–C** with an excess of Me_3_SiCN at various temperatures, but decomposition was invariably observed. In contrast, impure crude products **1A–C** neatly afforded the desired compounds **3A–C** upon treatment with a slight excess (0.3 eq.) of Me_3_SiCN at elevated temperature. The compounds **3A–C** show ^11^B{^1^H}-NMR-signals shifted further upfield, *e.g. δ*(^11^B) = –24.8 ppm, *ω*_1/2_ = 3 Hz for **3B**, and display two bands in the IR-spectra, as is expected for both the symmetric and antisymmetric C≡N stretch vibration, *e.g.* 2124 cm^–1^ and 2200 cm^–1^ in **3B**. Structural elucidation by X-ray crystallography confirmed the identity of **3A** with bonding parameters in the expected range (section ESI‡). The fact that the dicyanoboranes **3A–C** were only accessible from crude products **1A–C** or **2A–C** was confusing, and we hypothesised that trace amounts of B(SEt)_3_ in crude samples could be responsible for this unusual result. This assumption is corroborated by the observation that analytically pure samples of **1A–C** or **2A–C** readily afforded dicyanoboranes **3A–C** when they were doped with catalytic amounts of B(SEt)_3_. For further insight into this system we reacted B(SEt)_3_ with Me_3_SiCN in toluene at ambient temperature. Solution NMR-spectra of the reaction mixture revealed only one new boron species *δ*(^11^B) = –28.1 ppm, and we obtained compound **4**, which rapidly crystallised from the solution (Scheme 3). The formulation of compound **4** as a formal isonitrile-borane adduct Me_3_Si–N≡C–B(CN)_2_SEt was confirmed by X-ray crystallography, and in particular the assignment of the carbon and nitrogen atoms within the linear isonitrile moiety of **4** is unambiguous. This result is in sharp contrast to a previous report in which the analogous reaction of B(SMe)_3_ with Me_3_SiCN under comparable conditions gave the nitrile–borane adduct **5**.^23^ Although X-ray crystallographic data had not been reported by the authors the nature of **5** as a nitrile–borane adduct was clarified based on ^11^B{^1^H}-NMR chemical shifts, *i.e. δ*(^11^B) = 0.3 ppm for **5**, whereas we found *δ*(^11^B) = –28.1 ppm for the dissolved crystals of **4**. The reason for the formation of different products from very similar reactions is, however, currently unclear.

**Scheme 4:**
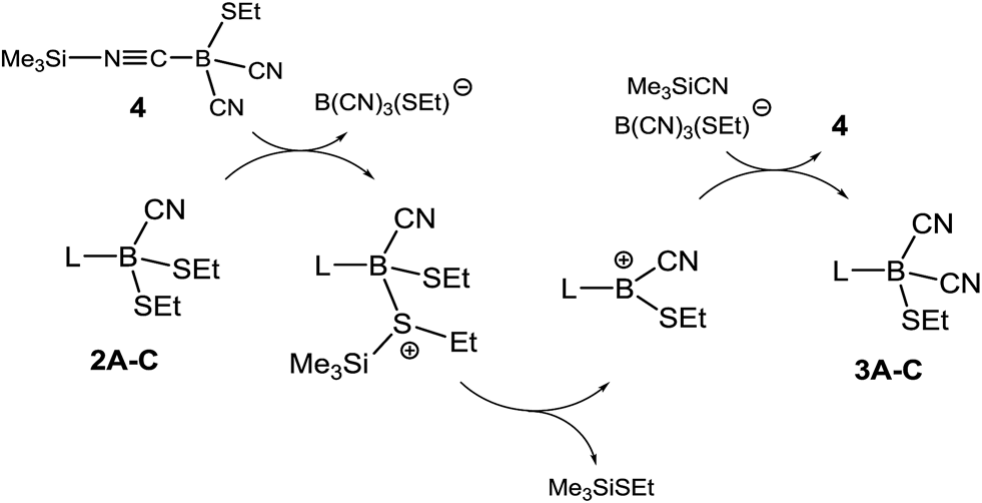
Proposed mechanism for the formation of dicyanoboranes **3A–C**. L = carbene moiety **A**, **B** or **C**.

We further investigated the role of **4** in the dicyanation step and found that analytically pure samples of **1A–C** or **2A–C** doped with catalytic amounts of isolated **4** also were efficiently dicyanated. Compound **4** is proposed in this system as an active source of silyl cations SiMe_3_^+^ (silylium ions) originating from heterolytic dissociation (Scheme 4). Silylium ions are widely known to be efficient Lewis acidic catalysts.^24^ The alkylthio groups in **2A–C** could be activated by the formation of sulfonium salts, from which thioether Me_3_SiSEt can readily be eliminated with concomitant formation of boryl cations L–B(CN)(SEt)^+^. The latter could react with Me_3_SiCN to form dicyanoboranes **3A–C** and re-form compound **4**.

The involvement of silylium ions was further investigated with trimethylsilyl perchlorate, Me_3_SiOClO_3_,^25^ which is a confirmed silylium transfer reagent. Indeed, analytically pure samples of **1A–C** or **2A–C** reacted with Me_3_SiCN (2.3 eq. or 1.3 eq., respectively) in the presence of Me_3_SiOClO_3_ (catalytic amounts) to afford dicyanoboranes **3A–C**, which provides strong support for the involvement of silylium cations in the dicyanation step. We observed no indication of the introduction of a third cyano group to give the percyanated species L–B(CN)_3_.

**Scheme 5:**
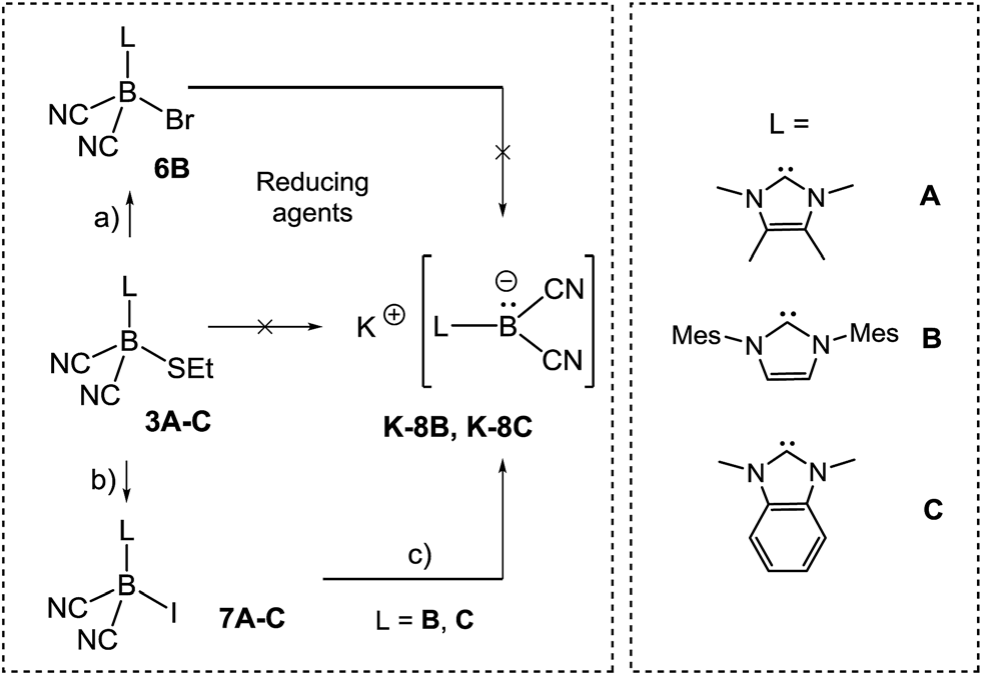
Reagents and conditions. (a) Br_2_, CH_2_Cl_2_, rt, 1 min; (b) MeI, adapted conditions **7A–C**; (c) for **K-8B**: 6 eq. KC_8_, THF, rt, 20 min; for **K-8C**: 6 eq. KC_8_, DME, rt, 20 min; for **K-8B** or **K-8C**: 6 eq. K in NH_3_, –60 °C, 20 min.

In approaches towards boryl anions of type **VIII**, we considered the ethylthio moieties in **3A–C** as conceivable reducible leaving groups (Scheme 5).

**Fig. 1:**
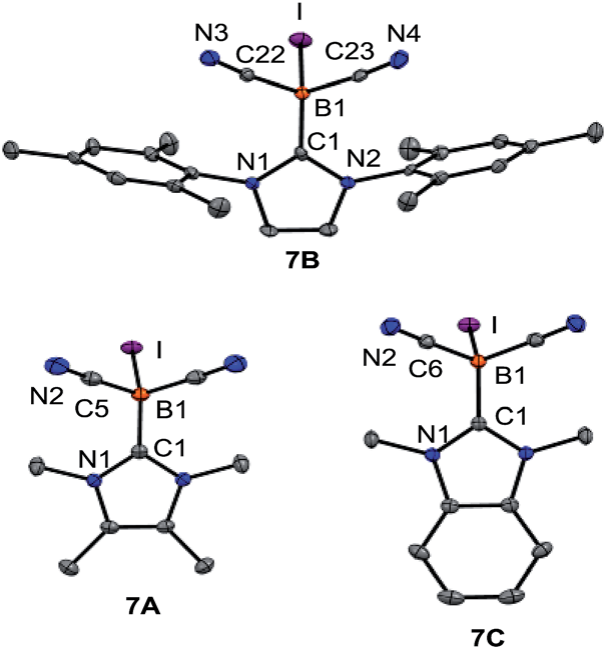
Molecular structures of iodoboranes **7A–C**. Thermal ellipsoids are presented at 50% probability levels. Hydrogen atoms omitted for clarity. Selected bond lengths (Å) and bond angles (°). For **7A** (CH_2_Cl_2_ omitted for clarity): I–B1 2.291(3), B1–C1 1.596(4), B1–C5 1.584(2), N2–C5 1.146(3), C1–B1–I 106.14(16), for **7B**, with three molecules in the asymmetric unit, average values are: I–B1 2.281(3), B1–C1 1.607(3), B1–C22 1.586(3), B1–C23 1.589(3), N3–C22 1.143(3), N4–C23 1.144(3), C1–B1–I 106.81(14), for **7C**: I–B1 2.271(2), B1–C1 1.605(3), B1–C6 1.584(2), N2–C6 1.146(2), C6–B1–I 108.35(10).

However, attempts to reduce **3A–C**, *e.g.* with KC_8_ or NaC_10_H_8_, gave a mixture of several species as assessed by ^11^B{^1^H}-NMR monitoring. A more successful approach involved the introduction of halogen atoms and their subsequent removal by reducing agents. Compound **3B** reacted with elemental bromine to give bromoborane **6B** with an ^11^B{^1^H} chemical shift of *δ*(^11^B) = –29.3 ppm, *ω*_1/2_ = 38 Hz. The same compound **6B** was formed from the parent hydroborane IMes–BH(CN)_2_ and bromine. Although B–Br bonds are expected to be labile, the reduction of bromoborane **6B** did not afford the boryl anion of type **VIII**. With the intention of introducing better leaving groups, we found that dicyanoboranes **3A–C** react with methyl iodide to give iodoboranes **7A–C**. In contrast, the parent hydroborane IMes–BH(CN)_2_ did not react with elemental iodine to yield the iodinated compound **7B**. The ^11^B{^1^H}-NMR spectra indicate a significant upfield shift of the signals with concomitant line broadening, *e.g. δ*(^11^B) = –24.8 ppm, *ω*_1/2_ = 3 Hz for **3B** *vs. δ*(^11^B) = –41.7 ppm, *ω*_1/2_ = 46 Hz for **7B**, which is consistent with spin–orbit coupling effects of iodine showing normal halogen dependence (NHD-effect).^26^ Structural authentication of compounds **7A–C** is provided by X-ray crystallographic analysis (Fig. 1). The iodoboranes **7A–C** display the expected tetrahedral geometry at boron. The bond lengths B1–I decrease in the order **7A** → **7B** → **7C** and correlate with the σ-donating properties of the carbene, which fall from **7A** to **7C**. Similarly, the bond angles I–B1–C1 increase systematically from **7A** to **7C**. The reduction of iodoboranes **7A–C** was attempted with KC_8_ (in THF or DME) and gave compounds **K–8B** and **K–8C** in 80–85% yield. The reactions required an excess of KC_8_ (6 eq.), lower quantities led to unidentified side products as indicated by ^11^B{^1^H}-NMR spectroscopy. The alternative reduction of **7B** or **7C** with K/NH_3_ afforded samples of comparable yield and purity.

**Fig. 2:**
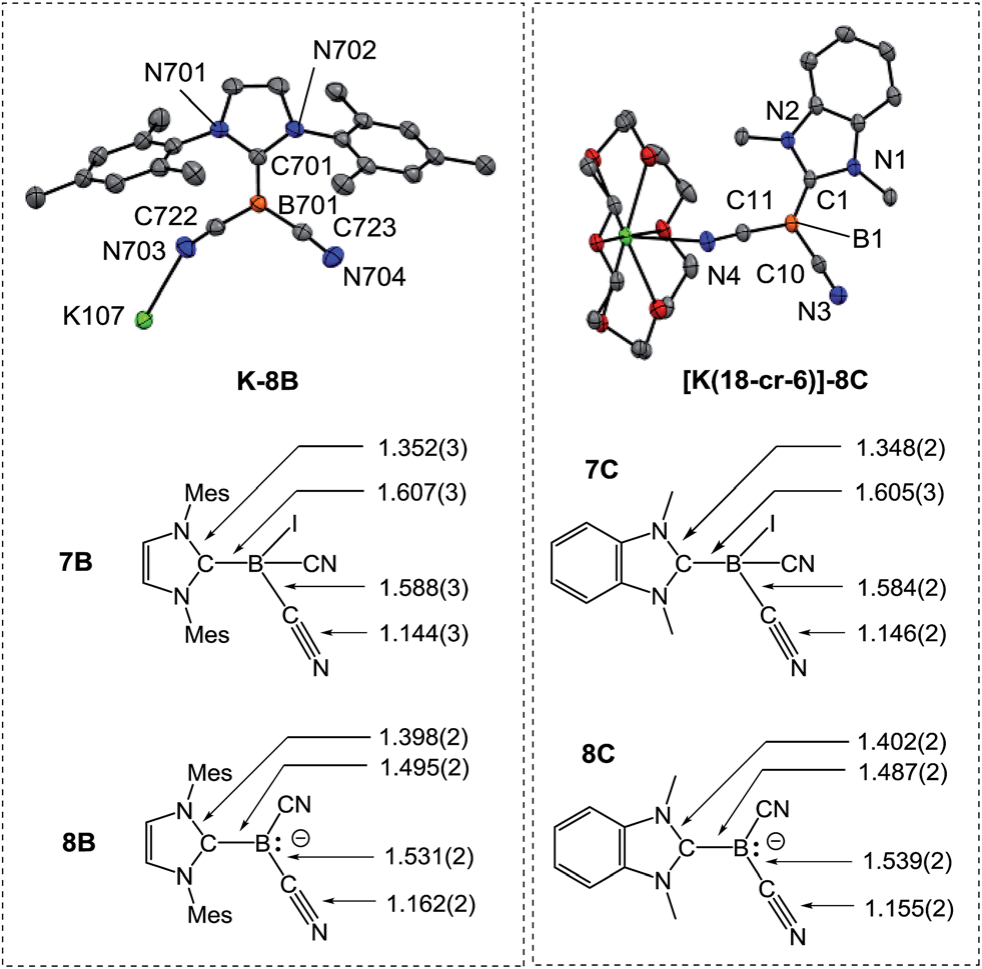
Molecular structures of compounds **K-8B** and **[K(18-cr-6)]-8C**. For **K-8B** a representative K[IMes–B(CN)_2_] unit of the nonamer is illustrated. **[K(18-cr-6)]-8C** crystallises as a bridged dimer located on a centre of inversion and only the symmetry independent unit is presented (for the dimer see section ESI‡). Thermal ellipsoids are presented at 50% probability levels. Hydrogen atoms omitted for clarity. The bond lengths in iodoboranes **7B**, **7C** and boryl anions **8B**, **8C** (reported in Å) represent average values calculated from each crystallographically independent molecule in the asymmetric unit.

In sharp contrast, the reduction of iodoborane **7A** under the same conditions did not yield the expected compound **K-8A**. Samples recorded after the reduction were ^11^B-NMR silent, whereas the anions **8B** and **8C** give rise to signals at *δ*(^11^B) = –28.3 ppm and *δ*(^11^B) = –24.1 ppm, respectively. Crystals suitable for X-ray crystallography were obtained from a solution of **K-8B** in THF by slow diffusion of pentane (Fig. 2 and section ESI‡). Compound **K-8B** crystallised as a nonamer of bridged K[IMes–B(CN)_2_] units. Compound **K-8C** crystallised in the presence of 18-crown-6 to afford [K(18-cr-6)][BAC–B(CN)_2_], **[K(18-cr-6)]-8C**, (Fig. 2).

The boron centres in the anions feature trigonal planar geometry. All B–C bonds of the boryl anions are shortened compared to the iodoboranes, whereby the bonds to the carbene are most affected. A lengthening of the C–N bonds is observed for both the nitrile groups and carbenes. These observations indicate strong resonance stabilisation of the boron-centred lone pair with the p_*z*_-orbital at the carbene carbon atom and the π*-orbital of the nitrile groups. These resonance effects are also obvious in the IR-spectra, in which the C≡N stretch vibrations are red-shifted, *e.g.* 2207 cm^–1^ in **7B** *vs.* 2090 cm^–1^ and 2123 cm^–1^ in **8B**. Theoretical calculations performed at the B3LYP-D3/TZVP level reliably reproduced both the bond lengths of the boryl anions **8B**, **8C** and the IR-spectra with respect to position and relative intensity of the C≡N stretch vibrations (section ESI‡).

**Fig. 3:**
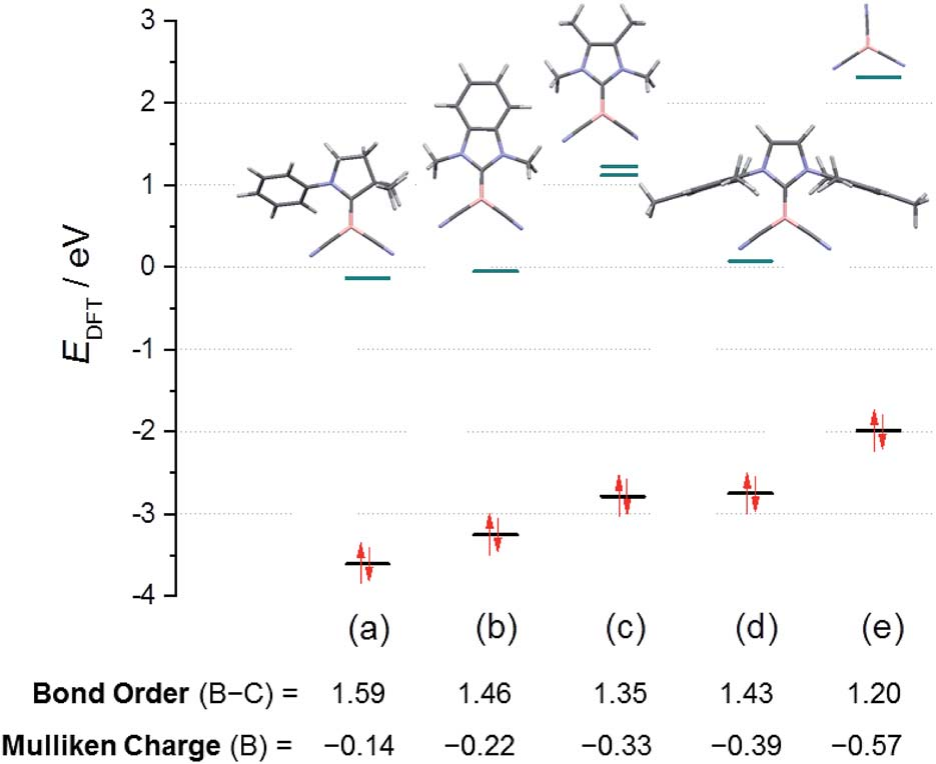
Energy levels of the frontier orbitals in the boryl anion series L–B(CN)_2_^–^, L = carbene or CN^–^: (a) truncated anion **VI**; (b) **8C**; (c) hypothetical **8A**; (d) **8B**; (e) anion **VII**.

For a systematic investigation we calculated frontier orbitals of the reported boryl anions **VI** and **VII** and compared them with our novel anions of type **VIII**. As expected, the HOMOs of the boryl anions are essentially boron-centred and display significant delocalisation into nitrile groups or carbene moieties (where applicable, section ESI‡). The energy levels of the HOMOs show a systematic increase in correlation with the falling π-acceptor properties of the substituents L in the anions L–B(CN)_2_^*n*–^, L = carbene or CN^–^ (Fig. 3). In particular, the anions **8B** and **8C** occupy a central position between the reported anions **VI** and **VII**. Interestingly, the attempted anion **8A** shows no peculiarities of its orbitals, and the failure to synthesize it must be of kinetic rather than thermodynamic origin. The decreasing π-acceptor character of the substituent L in the anions gives rise to a concomitant decrease of the bond order B–C between the boron centre and the substituent L, which is also consistent with increasingly negative Mulliken charges at boron. The nucleophilic activity of a Lewis base can be correlated with the energy level of its lone pair. In view of the Klopman–Salem-concept^27^ and in consideration of the structural similarity of the anions L–B(CN)_2_^–^ (boron as the nucleophilic centre, similar orbital shapes) the nucleophilicity can be expected to rise with increasing HOMO-energy. The distinct colours [**K-8C** – bright orange (*λ*_max_ < 400 nm) *vs.* **K-8B** – deep red (shoulder, *λ*_max_ = 480 nm)] are readily apparent from the lower HOMO–LUMO gap in **8B**. Detailed excited-state calculations reveal a HOMO → LUMO+1 transition (*f*_osc_ = 0.028, *λ*_max_ = 480 nm) in **8B**, which represents a charge-transfer from the essentially boron-centred lone-pair towards the asymmetric π*-orbitals of the mesityl substituent; the further red-shifted HOMO → LUMO transition is substantially less intense (*f*_osc_ = 0.005, *λ*_max_ = 518 nm). In contrast, the HOMO → LUMO transition in **8C** occurs from the lone pair into π*-orbitals of the phenylene moieties and is calculated to be below 400 nm (section ESI‡).

**Scheme 6:**
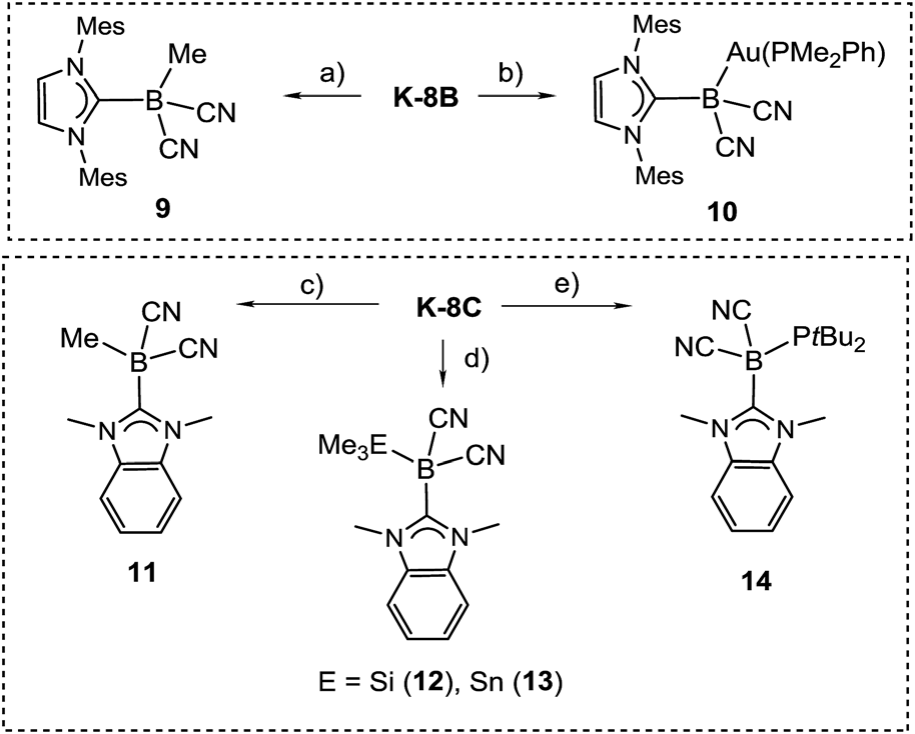
Reactions indicating the nucleophilic behaviour of boryl anions **8B** and **8C**. Reagents and conditions. (a) MeI, THF, rt, 10 min; (b) AuCl(PMe_2_Ph), THF, rt, 2 h; (c) MeI, THF, rt, 10 min; (d) Me_3_ECl, THF, 15 min, rt; (e) ClP(*t*Bu)_2_, DME, 1 h, rt.

The reactivity of the boryl anions **8B** and **8C** was probed with electrophiles, revealing a boron-centred nucleophilicity in both cases (Scheme 6). Thus, the reaction of **K-8B** with methyl iodide or gold electrophiles afforded the methylated species 9 or the gold boryl complex 10, giving rise to signals at *δ*(^11^B) = –27.6 or –29.7 ppm, respectively. Structural characterisation by X-ray crystallography was performed for **9** (section ESI‡), but no suitable crystals of **10** could be obtained. The identity of **10** is, among other data, unambiguously confirmed by the coupling of the ^11^B with the ^31^P nucleus, *i.e.* ^2^ *J*(^31^P–^11^B) = 65 Hz observed in both ^11^B{^1^H} and ^31^P{^1^H}-NMR spectra. Only three examples of gold boryl complexes are currently known^28^ but due to the lack of X-ray crystallographic analysis structural comparison cannot be drawn. The steric congestion by the carbene IMes in **8B** prevented simple reactions with bulkier electrophiles including Me_3_ECl (E = Si, Sn). Reactions of the sterically less crowded **8C** with main group electrophiles (including bulkier representatives) cleanly afforded boron-substituted products **11–14**, which were characterised by X-ray crystallography except for **12** (section ESI‡). Compound **13** shows a characteristic signal of *δ*(^11^B) = –35.9 ppm in the ^11^B{^1^H}-NMR spectrum accompanied by well resolved tin satellites ^1^ *J*(^117^Sn–^11^B) = 325 Hz, ^1^ *J*(^119^Sn–^11^B) = 338 Hz, while **14** resonates at *δ*(^11^B) = –29.6 ppm as a doublet, ^1^ *J*(^31^P–^11^B) = 24 Hz.

## Conclusions

The boryl anions of type NHC–B(CN)_2_^–^ described herein complete a consistent series with the known anions cAAC–B(CN)_2_^–^ (**VI**) and B(CN)_3_^2–^ (**VII**). Since N-heterocyclic carbenes are a thoroughly studied ligand class, their incorporation into NHC–B(CN)_2_^–^-systems essentially gives rise to the full scope of usual advantages, including a systematic variation of steric and electronic parameters, and in particular careful control of the nucleophilic properties at the boron centre. The novel approach towards NHC-stabilised cyanoboranes employs alkylthio-cyano exchange at boron and cleanly affords the mono- or dicyanated products [NHC–B(CN)(SEt)_2_ or NHC–B(CN)_2_SEt] while avoiding the isomeric isonitriles. The dicyanation step was shown to be silylium-catalysed. Facile iodination of dicyanated boranes to give NHC–B(CN)_2_I was shown to occur with methyl iodide. Only the iodoboranes were able to afford two novel boryl anions of type NHC–B(CN)_2_^–^ upon reduction, while other conceivable leaving groups – *e.g.* Br, SEt – were ineffective. Crystal structures and DFT calculations suggest a boron-centred lone pair, which is resonance-stabilised by π-acidic NHC and CN substituents. The energy level of the chiefly boron-centred HOMO, and thus the nucleophilicity, can be controlled by the π-acidity of the carbene. The species NHC–B(CN)_2_^–^ showed distinct boron-centred nucleophilicity with facile formation of B–E bonds, where E = C, Si, Sn, P, Au. Future investigations will concentrate on the preparation of further examples and the exploitation in salt metathesis reactions to form M–B bonds, M = metal or metalloid.
