# Supplementary material for: Completing the series of boron-nucleophilic cyanoborates: boryl anions of type NHC–B(CN)2 –
Source: Chem Sci. 2017 Jul 3;8(9):6274–80. doi: 10.1039/c7sc02238g (PMC5628389; doi:10.1039/c7sc02238g)
Supplement: Supplementary file 1 [file SC-008-C7SC02238G-s001.pdf]

Electronic Supplementary Material (ESI) for Chemical Science.
This journal is © The Royal Society of Chemistry 2017

Supporting Information

Completing the series of boron-nucleophilic cyanoborates: Boryl anions of type NHC−B(CN)$_2^-$

Richard Böser[a], Lisa C. Haufe[a], Matthias Freytag[a], Peter G. Jones[a], Gerald Hörner[b],*, René Frank[a]

[a] Institut für Anorganische und Analytische Chemie
Technische Universität Braunschweig
Hagenring 30, 38106 Braunschweig (Germany)
Tel.: (+49) 531-391-5308
E-mail: r.frank@tu-bs.de

[b] Institut für Chemie
Bioanorganische Chemie
Technische Universität Berlin
Straße des 17. Juni 135, 10623 Berlin (Germany)
Tel.: (+49) 30-314-27936
E-mail: gerald.hoerner@tu-berlin.de

Contents

Supporting Information 1

Synthetic details and procedures 2

Synthesis of IMes-substituted compounds..5

Synthesis of BAC-substituted compounds..16

Synthesis of IMe(Me)$_2$-substituted compounds 26

NMR Spectra 31

IMes-substituted compounds 31

BAC-substituted compounds 41

IMe(Me)$_2$-substituted compounds 51

X-ray Structure Analysis...55

DFT Calculations 69

Synthetic details and procedures

General Remarks: All procedures involving air- or moisture-sensitive compounds were performed in a glove box (MBraun 200B) under dry argon atmosphere or using Schlenk techniques. Solvents were purified using a Solvent Purification System (Braun) and stored over molecular sieve (3-4 Å). All commercially available compounds (TCI, abcr, Sigma Aldrich) were used without further purification if not otherwise stated. The compounds $B(SEt)_3$[1], Me_3SiCN [2], 1,3-dimethyl-benzimidazolium-iodide (BAC·HI)[3], **B** (IMes)[4] and **A** [IMe(Me)$_2$][5] were prepared according to literature methods. Abbreviations used:

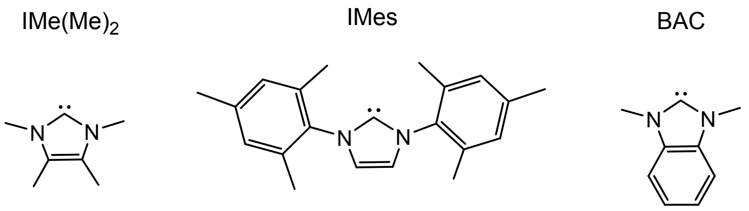

A **B** **C**

NMR spectra were recorded on Bruker Avance II-300, Avance III-HD, Avance III-400 and Bruker DRX-40 instruments. The chemical shifts (δ) are reported in parts per million (ppm).

The residual solvent peaks (THF-d_7, δ = 3.58 ppm; benzene-d_5, δ = 7.16 ppm; $CHCl_3$, δ = 7.26 ppm; toluene-d_7, δ = 2.08 ppm; $CHDCl_2$, δ = 5.32 ppm) are used for referencing of the ^{1}H spectra. The ^{13}C spectra are internally calibrated by using the ^{13}C resonances of the solvent peaks (THF-d_8, δ = 67.21 ppm; $CDCl_3$, δ = 77.16 ppm; benzene-d_5, δ = 128.06 ppm; toluene-d_8, δ = 20.43 ppm; CD_2Cl_2, δ = 53.84 ppm). For ^{19}F and ^{11}B NMR an external calibration with $CFCl_3$ and $BF_3 \cdot Et_2O$ was used. Coupling constants are stated in Hertz (Hz), multiplicities are defined as s (singlet), br s (broad singlet), d (doublet), t (triplet), q (quartet), br q (broad quartet), quin (quintet) or m (multiplet).

In general, ^{13}C-NMR NMR signals of boron bound carbon atoms are broad due to the quadrupolar environment of ^{10}B and ^{11}B nuclei and could only be detected for the compounds as stated. In all other cases the carbon signal is reported as not observed and further increase of the substance concentration led to signal broadening of the remaining 13C-NMR signals due to substance aggregation or elevated viscosity.

Elemental analyses were accomplished by combustion and gas chromatographic analysis using a VarioMICRO Tube and HW detection. Values are reported in %.

The IR spectra were recorded on a Bruker Vertex 70.

The UV/Vis spectra were recorded on a Varian Cary 50 Scan.

GC-MS were recorded on a Finnigan MAT 8400-MSS I, Finnigan MAT 4515 and Finnigan LCQDeca using ES and $ESI^{+/-}$ methods using a Finnigan MAT 95 XL Trap.

X-ray crystallography

Single crystals were mounted on glass fibres in perfluorinated oil. Intensity measurements (at 100 K) were registered on Oxford Diffraction diffractometers, either Xcalibur E with monochromated Mo *K*α or Nova A with mirror-focused Cu *K*α radiation. Absorption corrections were based on multi-scans. The structures were refined anisotropically on F^2 using the program SHELXL-97 or SHELXL-14.[6]

Treatment of hydrogen atoms: Hydrogen atoms were added using rigid methyl groups or a riding model.

Exceptions and special features: Compound **1B** was measured at 130 K because the crystals shatter at 100 K, presumably because of a phase transition. It is achiral and crystallizes only by chance in a chiral (Söhncke) space group (the same is true of **3A**). For compound **2B**, the methyl hydrogens at C11 were refined in an idealized hexagon of half-occupied sites.

Compound **7A** crystallizes as a dichloromethane solvate; both residues display crystallographic mirror symmetry. Compound **7B** crystallizes as a pseudomerohedral twin because its β angle is close to 90°; the relative volume of the smaller twin component refined to 0.4131(4). The asymmetric unit contains three molecules related approximately by the translation **c**/3; however, the separation is not exact. Each molecule displays approximate mirror symmetry (r.m.s deviations 0.08, 0.05, 0.03 Å). The molecule of **7C** displays crystallographic mirror symmetry. For **K-8B**, regions of badly disordered THF were removed with the program SQUEEZE;[13] the quoted composition, formula mass etc. involve an idealized content of 4 THF per asymmetric unit. The compound **[K(18-cr-6)]-8C** crystallizes as an inversion-symmetric dimer. Compound **11** crystallizes with two independent molecules, each with mirror symmetry; the dichloromethane molecule also has mirror symmetry. Compound **14** crystallizes as a dichloromethane solvate.

For those molecules not shown as Figures in the main publication, ellipsoid diagrams are given below (*pp*. 58 *ff*.). For all compounds with informative packing diagrams, these are also given.

Complete data have been deposited at the Cambridge Crystallographic Data Centre under the CCDC numbers 1550754 (**1B**), 1550755 (**2B**), 1550760 (**7B**), 1550767 (**K-8B**), 1550769 (**9**), 1550740 (**1C**), 1550756 (**2C**), 1550774 (**7C**), 1550768 {**[K(18-cr-6]-8C**}, 1550770 (**11**), 1550771 (**13**), 1550772 (**14**), 1550757 (**3A**), 1550759 (**7A**), 1550758 (**4**) and can be obtained free of charge at http://www.ccdc.cam.ac.uk/.

Synthesis of IMes-substituted compounds.

IMes-B(SEt)$_3$ (1B).

B(SEt)$_3$ (1.28 g, 6.57 mmol) was added to a solution of IMes (2.00 g, 6.57 mmol) in toluene (25 mL). After stirring for 5 min, pentane (40 mL) was added to complete the precipitation of a crystalline solid. After filtration, washing with pentane (40 mL) and drying *in vacuo* the product (2.16 g, 4.34 mmol, 66%) was obtained as colorless crystalline material. Crystals suitable for X-ray crystallography were obtained by slow diffusion of pentane into a solution of the product in THF.

^{1}H NMR (300.1 MHz, C_6D_6): δ (ppm) = 6.75 (m, 4 H, Mes-*meta*-C*H*), 5.76 (s, 2 H, NC*H*), 2.57 (br q, 6 H, BSCH_2, $^3J_{HH}$ = 7.4 Hz), 2.24 (s, 12 H, Mes-*ortho*-CH_3), 2.10 (s, 6 H, Mes-*para*-CH_3), 1.32 (t, 9 H, BSCH$_2$CH_3, $^3J_{HH}$ = 7.5 Hz).

^{13}C{^{1}H} NMR (75.5 MHz, C_6D_6): δ (ppm) = 139.3 (s, Mes-*para*-*C*), 136.1 (s,Mes-*ipso*-C), 135.9 (s, Mes-*ortho*-*C*), 129.2 (s, Mes-*meta*-*C*H), 123.1 (s, N*C*H), 24.1 (s, BS*C*H$_2$CH$_3$), 21.1 (s, Mes-*para*-*C*H$_3$), 19.1 (s, 4 C, Mes-*ortho*-*C*H$_3$), 17.7 (s, BSCH$_2$*C*H$_3$), not observed (B*C*N).

^{11}B{^{1}H} NMR (96.3 MHz, C_6D_6): δ (ppm) = −2.0 (s, $\omega_{½}$ = 3 Hz).

Elemental Analysis. Calcd. for $C_{27}H_{40}BN_2S_3$: C 65.04, H 7.88, N 5.62. Found: C 64.97, H 7.83, N 5.82.

MS: Representative mass spectra could not be recorded.

IR (ATR, cm^{-1}): $\tilde{\nu}$ = 3159 (w), 3100 (w), 2976 (m), 2956 (m), 2919 (m), 2861 (m), 1609 (w), 1566 (w), 1483 (m), 1453 (m), 1408 (m), 1376 (m), 1222 (m), 854 (s), 773 (s), 742 (s), 684 (s).

IMes-B(SEt)$_2$CN (2B).

A solution of Me_3SiCN (56 mg, 0.57 mmol) in benzene (0.5 mL) was slowly added to IMes-B(SEt)$_3$ (**1B**, 101 mg, 0.20 mmol) dissolved in toluene (2 mL). The mixture was heated to 45 °C for 24 h, and colorless crystals precipitated. The supernatant solution was removed, the solid residue washed with toluene (0.6 mL) and dried *in vacuo* (59 mg, 0.13 mmol, 64%). Crystals suitable for X-ray crystallography were obtained by slow diffusion of hexane into a solution of the product in THF.

^{1}H NMR (400 MHz, CD_2Cl_2): δ (ppm) = 7.05 (s, 2H, NC*H*), 7.02-7.03 (m, 4H, Mes-*meta*-C*H*), 2.38 (s, 6 H, Mes-*para*-CH_3), 2.25 (q, 6 H, BSCH_2, $^3J_{HH}$ = 7.5 Hz), 2.20 (s, 12 H, Mes-*ortho*-CH_3), 1.08 (t, 6 H, BSCH2C*H*3, $^3J_{HH}$ = 7.5 Hz).

^{13}C{^{1}H} NMR (100 MHz, CD_2Cl_2): δ (ppm) = 140.7 (s, Mes-*para*-*C*), 136.2 (s, Mes-*ipso*-C), 134.2 (s, Mes-*ortho*-*C*), 129.3 (s, Mes-*meta*-*C*H), 124.6 (s, BCN*C*H), 25.2 (s, BS*C*H_2CH_3), 21.3 (s, Mes-*para*-*C*H_3), 18.6 (s, Mes-*ortho*-*C*H_3), 16.8 (s, BSCH_2*C*H_3), not observed (B*C*N).

^{11}B{^{1}H} NMR (96.3 MHz, CD_2Cl_2): δ (ppm) = −13.7 (s, $\omega_{1/2}$ = 3 Hz).

Elemental Analysis. Calcd. for $C_{26}H_{35}BN_3S_2$: C 67.37, H 7.39, N 9.07. Found: C 67.38, H 7.08, N 8.89.

MS (EI): m/z = 402.2 [M−SEt]$^+$.

IR (ATR, cm^{-1}): $\tilde{\nu}$ = 3171 (w), 3120 (w), 3081 (w), 2976 (w), 2914 (m), 2859 (w), 2736 (w), 2194 (w), 1764 (w), 1731 (w), 1610 (w), 1564 (w), 1485 (m), 1440 (m), 1415 (m), 1372 (w), 1301 (w), 1260 (m), 1230 (m), 1172 (w), 1150 (w), 1091 (w), 1060 (w), 1038 (w), 1017 (w), 984 (w), 967 (w), 931 (w), 901 (w), 879 (m), 864 (m), 850 (m), 784 (s), 768 (m),750 (m), 737 (m), 696 (s), 672 (m).

IMes-B(CN)$_2$SEt (3B).

Me_3SiCN (2.28 g, 23 mmol) dissolved in toluene (15 mL) was added dropwise to a solution of IMes-B(SEt)$_3$ (**1B**, 5.00 g, 10 mmol) in toluene (50 mL). After addition of B(SEt)$_3$ (150 mg, 0.77 mmol) the reaction mixture was heated to 95 °C for 12 h, whereupon the product precipitated. The hot suspension was filtered and the solid product (2.72 g, 6.35 mmol, 64%) was dried *in vacuo*. The material was sufficiently pure for further reactions. Analytically pure samples could be obtained by washing the product with hot toluene several times.

^{1}H NMR (400 MHz, CD_2Cl_2): δ (ppm) = 7.19 (s, 2 H, BCNC*H*), 7.06-7.08 (m, 4 H, Mes-*meta*-C*H*), 2.39 (s, 6 H, Mes-*para*-C*H*$_3$), 2.36-2.28 (m, 2 H, BSC*H*$_2$), 2.17 (s, 12 H, Mes-*ortho*-C*H*$_3$), 1.12 (t, 3 H, BSCH$_2$C*H*$_3$, $^3J_{HH}$ = 7.4 Hz).

^{13}C{^{1}H} NMR (100 MHz, CD_2Cl_2): δ (ppm) = 141.5 (s, Mes-*para-C*), 136.0 (s, Mes-*ipso-C*), 133.0 (s, Mes-*ortho-C*), 129.7 (s, Mes-*meta*-*C*H), 125.2 (s, N*C*H), 25.1 (s, S*C*H$_2$CH$_3$), 21.4 (s, Mes-*para-C*H$_3$), 18.3 (s, Mes-*ortho*-*C*H$_3$), not observed (SCH$_2$*C*H$_3$), not observed (B*C*N).

^{11}B{^{1}H} NMR (96.3 MHz, CD_2Cl_2): δ (ppm) = −24.8 (s, $\omega_{½}$ = 3 Hz).

Elemental Analysis. Calcd. for $C_{25}H_{29}BN_4S$: C 70.09, H 6.82, N 13.08, S 7.48. Found: C 69.75, H 6.88, N 12.64, S 7.23.

MS (EI): m/z = 428.2 [M]$^+$, 367.2 [M−SEt]$^+$.

IR (ATR, cm^{-1}): $\tilde{\nu}$ = 3165 (w), 3133 (w), 3100 (w), 2972 (w), 2925 (w), 2857 (w), 2732 (w), 2200 (w), 2124 (w), 1723 (w), 1610(w), 1561 (w), 1485 (m), 1440 (m), 1375 (w), 1322 (w), 1290 (w), 1262 (w), 1236 (m), 1177 (w), 1160 (w), 1112 (w), 1067 (w), 1038 (w), 1001 (w), 977 (w), 951 (w), 933 (m), 918 (w), 897 (m), 867 (m), 849 (m), 815 (w), 793 (m), 767 (m), 742 (m), 711 (s), 674 (w), 618 (w).

Me_3Si-N≡C-B(CN)$_2$SEt (4).

B(SEt)$_3$ (490 mg, 2.53 mmol) was added to a solution of Me_3SiCN (910 mg, 9.19 mmol) in toluene (3 mL). After 30 min a crystalline precipitate had formed. The supernatant solution was decanted and the remaining solids were washed with toluene. After drying *in vacuo* the product (230 mg, 1.03 mmol, 41%) was obtained as colorless crystals.

^{1}H NMR (300 MHz, CD_2Cl_2): δ (ppm) = 2.60 (q, 2 H, SCH_2CH$_3$, $^3J_{HH}$ = 7 Hz), 1.33 (t, 3 H, SCH$_2$CH_3, $^3J_{HH}$ = 7 Hz), 0.62 [s, 9 H, Si(CH_3)$_3$].

^{13}C{^{1}H} NMR (75 MHz, CD_2Cl_2): δ (ppm) = 24.6 (s, SCH$_2$CH$_3$), 16.9 (s, BSCH$_2$$CH_3$), −0.8 [s, Si($CH_3$)$_3$].

^{11}B{^{1}H} NMR (96.3 MHz, CD_2Cl_2): δ (ppm) = −28.1 (s, $\omega_{½}$ = 14 Hz).

Elemental Analysis. Calcd. for $C_8H_{14}BN_3SSi$: C 43.05, H 6.32, N 18.83, S 14.37. Found: C 42.74, H 6.13, N 18.39, S 13.91.

MS (EI): m/z = 124.0 [B(CN)$_2$SEt]$^+$.

IR (ATR, cm^{-1}): $\tilde{\nu}$ = 2976 (w), 2930 (w), 2871 (w), 2615 (w), 2316 (w), 2271 (m), 2223 (w), 2034 (w), 1975 (w), 1892 (w), 1671 (w), 1494 (w), 1457 (w), 1438 (w), 1375 (w), 1254 (m), 1061 (w), 984 (w), 951 (w), 904 (w), 864 (s), 843 (s), 795 (m), 774 (s), 677 (w), 634 (m).

IMes-B(CN)$_2$Br (6B).

Br_2 (56 mg, 0.35 mmol) was added to a solution of IMes-B(CN)$_2$SEt (**3B**, 150 mg, 0.35 mmol) in CH_2Cl_2 (20 mL). After stirring for 90 s, the pale brown solution was evacuated to dryness. The remaining solid was recrystallized twice by slow diffusion of pentane into a solution of the solid in $CHCl_3$. The product was obtained as a pale pink powder (104 mg, 0.23 mmol, 67%).

In an alternative approach IMes-B(CN)$_2$H (368 mg, 1.00 mmol) was dissolved in CH_2Cl_2 (20 mL). Br_2 (160 mg, 1.00 mmol) was added and the red solution was stirred for 5 min. All volatile components were removed *in vacuo*. The remaining solid was recrystallized by slow diffusion of pentane into a solution of the solid in $CHCl_3$. The product was obtained as a colorless powder (344 mg, 0.77 mmol, 77%).

Samples obtained from both procedures gave identical ^{1}H and ^{11}B NMR spectra.

^{1}H NMR (400 MHz, CD_2Cl_2): δ (ppm) = 7.26 (s, 2 H, NC*H*), 7.08 (m, 4 H, Mes-*meta*-C*H*), 2.39 (s, 6 H, Mes-*para*-C*H*$_3$), 2.16 (s, 12 H, Mes-*ortho*-C*H*$_3$).

^{13}C{^{1}H} NMR (150 MHz, CD_2Cl_2): δ (ppm) = 141.9 (s, Mes-*para-C*), 135.8 (s, Mes-*ipso*-C), 132.4 (s, Mes-*ortho*-*C*), 129.9 (s, Mes-*meta*-*C*H), 125.5 (s, N*C*H), 21.4 (s, Mes-*para*-*C*H$_3$), 18.3 (s, Mes-*ortho*-*C*H$_3$), not observed (B*C*N).

^{11}B{^{1}H} NMR (128.5 MHz, CD_2Cl_2): δ (ppm) = −29.3 (s, $\omega_{½}$ = 38 Hz).

Elemental Analysis. Calcd. for $C_{23}H_{24}BBrN_4$: C 61.78, H 5.41, N 12.53. Found: C 61.74, H 5.35, N 12.23.

MS (EI): m/z = 446.1 $[M]^+$, 420.1 $[M-CN]^+$.

IR (ATR, cm^{-1}): $\tilde{\nu}$ = 3160 (w), 3135 (w), 2920 (w), 2854 (w), 2211 (w), 1748 (w), 1633 (w), 1609 (w), 1561 (w), 1483 (m), 1454 (m), 1414 (w), 1380 (m), 1323 (w), 1290 (w), 1233 (m), 1261 (w), 1184 (w), 1134 (m), 1178 (w), 1163 (w), 1094 (w), 1035 (w), 980 (m), 947 (w), 932 (w), 896 (w), 874 (m), 857 (m), 778 (s), 681 (s).

IMes-B(CN)$_2$I (7B).

Methyl iodide (9.10 g, 64 mmol) was added to a solution of IMes-B(CN)$_2$SEt (**3B**, 2.75 g, 6.4 mmol) in CH_2Cl_2 (120 mL). After stirring for 3 d the mixture was washed with brine (3 × 100 mL). The organic phase was dried over $MgSO_4$, and the solvent was removed *in vacuo*. The solid residue was recrystallized by slow diffusion of pentane into a highly concentrated solution in CH_2Cl_2. After 5 d the supernatant solution was removed, the colorless crystals (1.5 g, 3 mmol, 47%) were washed with CH_2Cl_2 (3×5 mL) and dried *in vacuo*. A second crop obtained from the yellow solution increased the yield to 68%.

^{1}H NMR (400 MHz, CD_2Cl_2): δ (ppm) = 7.26 (s, 2 H, NC*H*), 7.08 (br s, 4 H, Mes-*meta*-C*H*), 2.40 (s, 6 H, Mes-*para*-CH_3), 2.19 (s, 12 H, Mes-*ortho*-CH_3).

^{13}C{^{1}H} NMR (100 MHz, CD_2Cl_2): δ (ppm) = 142.0 (s, Mes-*para*-*C*), 135.7 (s, Mes-*ipso*-C), 132.4 (s, Mes-*ortho*-*C*), 130.1 (s, Mes-*meta*-*C*H), 125.7 (s, N*C*H), 21.4 (s, Mes-*para*-*C*H$_3$), 18.9 (s, Mes-*ortho*-*C*H$_3$), not observed (B*C*N).

^{11}B{^{1}H} NMR (96.3 MHz, CD_2Cl_2): δ (ppm) = −41.7 (s, $\omega_{½}$ = 46 Hz).

Elemental Analysis. Calcd. for $C_{23}H_{25}BIN_4$: C 55.90, H 4.90, N 11.34. Found: C 55.67, H 4.81, N 11.32.

MS (EI): m/z = 494.0 [M]$^+$, 367.2 [M−I]$^+$.

IR (ATR, cm^{-1}): $\tilde{\nu}$ = 3164 (w), 3138 (w), 2921 (w), 2858 (w), 2733 (w), 2207 (w), 1803 (w), 1766 (w), 1611 (w), 1561 (w), 1484 (m), 1449 (m), 1410 (w), 1378 (m), 1322 (w), 1291 (w), 1233 (m), 1184 (w), 1167 (m), 1095 (w), 1036 (w), 990 (w), 956 (m), 930 (w), 897 (w), 876 (s), 864 (s), 775 (s), 727 (m), 703 (w), 683 (w), 666 (m), 648 (s).

K[IMes-B(CN)$_2$] (K-8B).

IMes-B(CN)$_2$I (**7B**, 1.4 g, 2.8 mmol) was added to a suspension of KC$_8$ (2.28 g, 16.8 mmol) in THF (30 mL). After stirring for 20 min at r.t. the mixture was filtered. The solution was dried *in vacuo* to obtain the product as a deep-red solid (966 mg, 2.38 mmol, 85% yield). Crystals suitable for X-ray crystallography were obtained by slow diffusion of pentane into a solution of the product in THF.

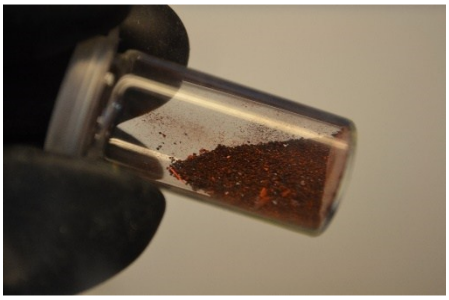

^{1}H NMR (300 MHz, THF-*d8*): δ (ppm) = 6.76 (br s, 4 H, Mes-*meta*-C*H*), 6.1 (br s, NC*H*), 2.20 (br s, 6 H, Mes-*para*-C*H$_3$*), 2.15 (br s, 12 H, Mes-*ortho*-C*H*$_3$).

^{13}C{^{1}H} NMR (75 MHz, THF-*d8*): δ (ppm) = 138.5 (s, Mes-*para-C*), 137.7 (s, Mes-*ipso*-C), 136.1 (s, Mes-*ortho-C*), 128.8(s, Mes-*meta*-*C*H), 115.7 (s, N*C*H), 21.2 (s, Mes-*para*-*C*H$_3$), 18.8 (s, Mes-*ortho*-*C*H$_3$), not observed (B*C*N).

^{11}B{^{1}H} NMR (96.3 MHz, THF-*d8*): δ (ppm) = −28.3 (s, $\omega_{1/2}$ = 107 Hz).

MS: Representative mass spectra could not be recorded.

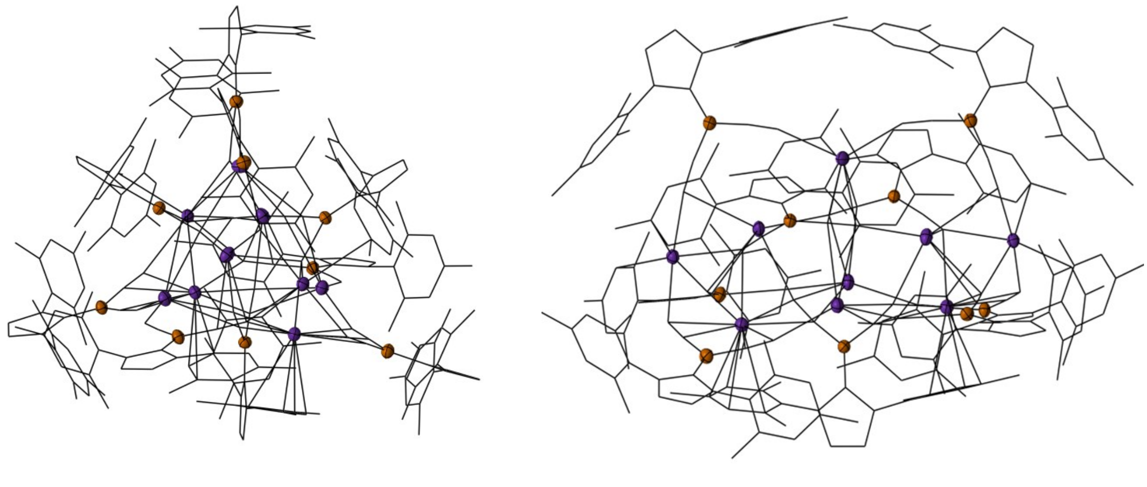

Figure 1:Molecular structure of ***(K-8B)$_9$*** *(nonameric unit) as found in the asymmetric unit. THF solvent molecules were treated with SQUEEZE for clarity. Hydrogen atoms omitted.*

Experimental IR spectrum of [**K(18-cr-6)]-8B**, THF solution, cm^{-1}: υ = 2123 (s), 2090 (s).

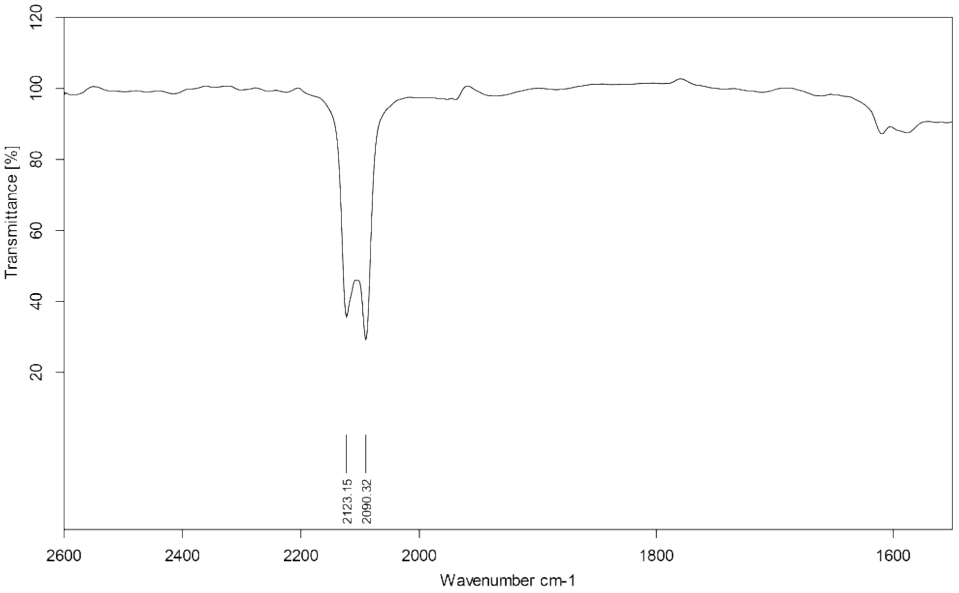

*Figure 2: Experimental IR spectrum of **[K(18-cr-6)]-8B**.*

Calculated IR spectrum of **8B**, cm^{-1}: υ = 2140 (s), 2055 (m).

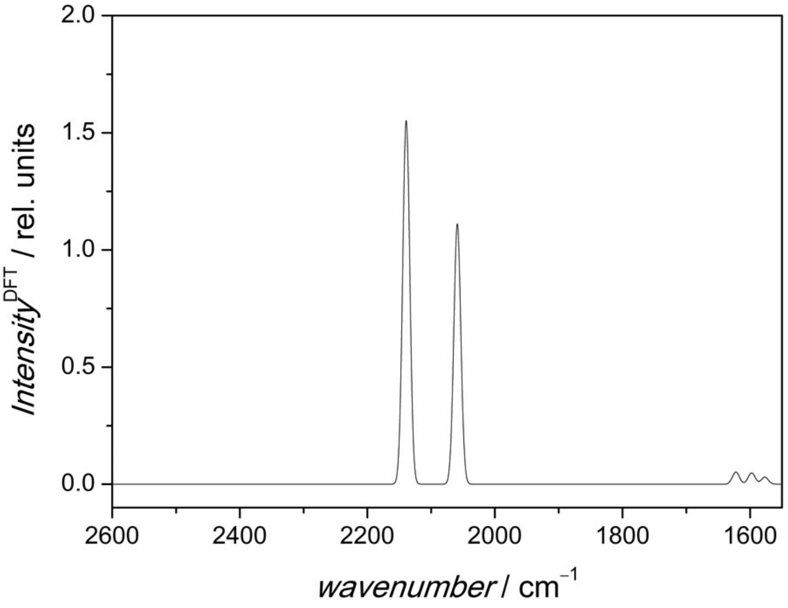

*Figure 3: Calculated IR spectrum of anion **8B**.*

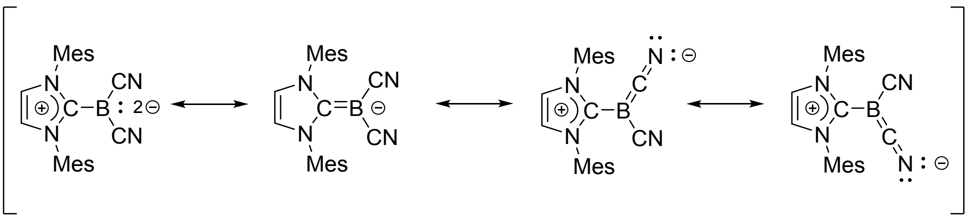

Experimental UV-Vis (THF):

λ_{max} = 361 nm, $\varepsilon = 9.96 \cdot 10^4$ L·mol^{-1}·cm^{-1}, shoulder λ_{max} = 480 nm, $\varepsilon = 5.52 \cdot 10^3$ L·mol^{-1}·cm^{-1}.

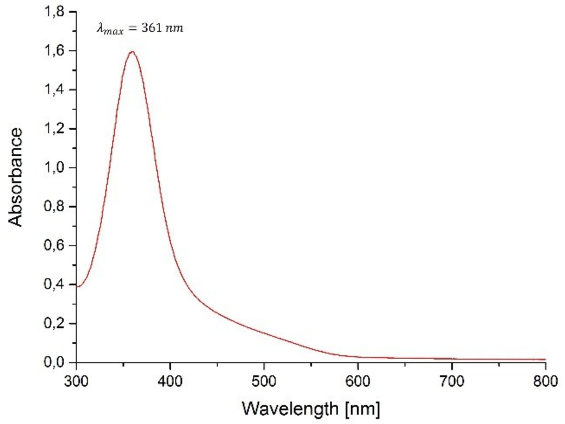

*Figure 4: Experimental UV-Vis spectrum of **K-8B**.*

TD-DFT analysis diagnostic UV-VIS transitions:

Intense charge transfer within the B−C carbene bond, partly delocalized (f_{osc} = 0.507, λ = 295 nm) HOMO → LUMO+4 transition (60%).

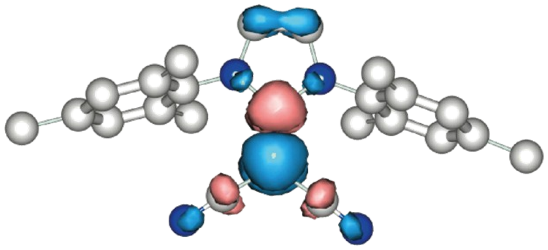

*Figure 5: Computed difference electron density of N-UV-transition 19 in anion **8B** (blue: donor orbitals, red: acceptor orbitals).*

Moderately intense charge transfer from the boron-centred lone pair into the symmetrical π-orbital of the mesityl groups (f_{osc} = 0.028, λ = 478 nm) HOMO → LUMO+1 transition (98%).

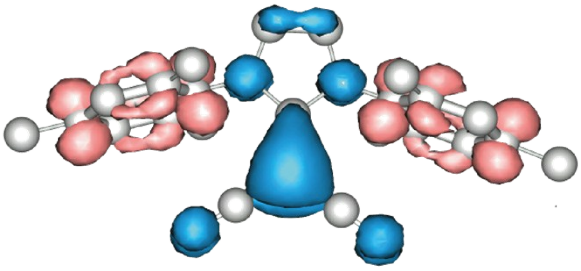

*Figure 6: Computed difference electron density of Vis-transition 5 in anion **8B** (blue: donor orbitals, red: acceptor orbitals).*

IMes-B(CN)$_2$Me (9).

Methyl iodide (0.05 mL, 0.80 mmol) was added dropwise to a solution of **K-8B** (300 mg, 0.74 mmol) in THF (15 mL). A colorless precipitate formed immediately. After stirring for 10 min at r.t. the precipitate was removed by filtration. The filtrate was evaporated to dryness *in vacuo* and the solid recrystallized by slow diffusion of pentane into a solution of crude product in $CHCl_3$ (42 mg, 0.12 mmol, 15%).

^{1}H NMR (600 MHz, $CDCl_3$): δ (ppm) = 7.13 (s, 2 H, NC*H*), 7.05 (s, 4 H, Mes-*meta*-C*H*), 2.37 (s, 6 H, Mes-*para*-CH_3), 2.14 (s, 12 H, Mes-*ortho*-CH_3), -0.27 (s, 3 H, BCH_3).

^{13}C{^{1}H} NMR (150 MHz, $CDCl_3$): δ (ppm) = 141.0 (s, Mes-*para-C*), 135.2 (s, Mes-*ortho-C*), 132.9 (s, Mes-*ipso-C*), 129.6 (s, Mes-*meta-C*), 124.2 (s, N*C*H), 21.3 (s, Mes-*para*-*C*H$_3$), 17.8 (s, Mes-*ortho*-*C*H$_3$), not observed (B*C*N).

^{11}B{^{1}H} NMR (96.3 MHz, $CDCl_3$): δ (ppm) = −27.6 (s, $\omega_{½}$ = 44 Hz).

Elemental Analysis. Calcd. for $C_{23}H_{25}BIN_4$: C 75.40, H 7.12, N 14.65. Found: C 75.12, H 7.13, N 14.36.

MS: Representative mass spectra could not be recorded.

IR (ATR, cm^{-1}): ν = 3163 (w), 2980 (s), 2919 (m), 2201 (w), 1654 (w), 1610 (w), 1565 (w), 1486 (s), 1448 (s), 1381 (m), 1315 (w), 1235 (s), 1156 (s), 1006 (s), 965 (s), 930 (s), 854 (s), 770 (m), 723 (s).

IMes-B(CN)$_2$Au(PMe$_2$Ph) (10).

AuCl(PMe$_2$Ph) (187 mg, 0.51 mmol) in THF (4 mL) was added to a solution of **K-8B** (207 mg, 0.51 mmol) in THF (20 mL). After stirring for 2 h, the mixture was filtered, and the filtrate was evaporated to dryness *in vacuo*. The product was obtained as a red solid (190 mg, 0.27 mmol, 53%). Analytically pure samples were obtained by repeated (three times) slow diffusion of pentane into a solution of the crude product in THF to afford a colorless crystalline material.

^{1}H NMR (300 MHz, THF-d$_8$): δ (ppm) = 7.68-7.61 (m, 2 H, Ph-C*H*), 7.43-7.39 (m, 3 H, Ph-C*H*), 7.10 (s, 2 H, NC*H*), 6.88-6.87 (m, 4 H, Mes-*meta*-C*H*), 2.26 (s, 6 H, Mes-*para*-CH_3), 2.18 (s, 12 H, Mes-*ortho*-CH_3), 1.55 (d, $^2J_{PH}$ = 8 Hz, 6 H, PCH_3).

^{13}C{^{1}H} NMR (75 MHz, THF-d$_8$): δ (ppm) = 139.7 (s, Mes-*para-C*), 136.5 (s, Mes-*ortho-C*), 134.8 (s, Mes-*ipso-C*), 132.5 (d, $^2J_{PC}$ = 13 Hz, Ph-*ortho*-*C*H), 130.8 (s, Ph-*para*-*C*H), 129.6 (s, Mes-*meta-C*), 129.3 (d, $^3J_{PC}$ = 10 Hz, Ph-*meta*-*C*H), 122.7 (s, N*C*H), 21.1 (s, Mes-*para*-*C*H$_3$), 18.9 (s, Mes-*ortho*-*C*H$_3$), 14.7 (d, $^1J_{PC}$ = 24 Hz, P-*C*H$_3$), not observed (B*C*N), not observed (Ph-*ipso*-*C*).

^{11}B{^{1}H} NMR (96.3 MHz, THF-d$_8$): δ (ppm) = −29.7 [d, $^2J(^{31}P\text{-}^{11}B)$ = 65 Hz, $\omega_{1/2}$ = 116 Hz].

^{31}P{^{1}H} NMR (121.5 MHz, THF-d$_8$): δ (ppm) = 27.7 [d, $^2J(^{31}P\text{-}^{11}B)$ = 70 Hz].

Elemental Analysis. Calcd. for $C_{31}H_{35}AuBN_4P$: C 53.01, H 5.02, N 7.98. Found: C 53.45, H 5.34, N 7.26.

MS: Representative mass spectra could not be recorded.

IR (ATR, cm^{-1}): $\tilde{\nu}$ = 3154 (w), 2971 (w), 2912 (w), 2856 (w), 2164 (m), 1610 (w), 1562 (w), 1486 (m), 1435 (m), 1408 (m), 1375 (w), 1310 (m), 1232 (s), 1183 (w), 1150 (w), 1105 (m), 1063 (m), 1032 (w), 982 (s), 953 (m), 908 (s), 848 (m), 807 (w), 695 (m), 649 (w).

Synthesis of BAC-substituted compounds

BAC-B(SEt)$_3$ (1C).

A solution of NaHMDS (6.62 g, 36.1 mmol) in THF (30 mL) was slowly added to a cooled (−78 °C) suspension of 1,3-dimethyl-1*H*-benzimidazolium iodide (9.89 g, 36.1 mmol) and THF (40 mL). After stirring for 5 min at −78 °C a solution of B(SEt)$_3$ (7.01 g, 36.1 mmol) in THF (20 mL) was added dropwise. The yellow solution was warmed to room temperature and stirred for 3 d. The solution was filtered to remove precipitated salts, and the filtrate was evaporated to dryness *in vacuo*. The residue was dissolved in toluene (100 mL). The opaque solution was filtrated and evaporated to dryness. The remaining solid was washed with pentane (50 mL) to afford a colorless powder (9.19 g, 26.9 mmol, 75%). Crystals suitable for X-ray crystallography were obtained by slow diffusion of hexane into a solution of the product in toluene.

^{1}H NMR (600 MHz, toluene-*d8*): δ(ppm) = 6.89-6.87 (m, 2 H, ar-H), 6.62-6.60 (m, 2 H, ar-H), 4.10 (s, 6H, NCH_3), 2.77-2.73 (m, 6H, SCH_2CH$_3$), 1.31 (t, $^3J_{HH}$=7.5 Hz, 9H, SCH$_2$$CH_3$).

^{13}C{^{1}H} NMR (150 MHz, toluene-*d8*): δ(ppm) = 133.5 (s, ar-*C*N), 124.7 (s, ar-*C*H), 111.3 (s, ar-*C*H), 34.3 (s, N*C*H$_3$), 23.8 (s, SCH_2CH$_3$), 17.7(s, SCH$_2$$CH_3$), not observed (B*C*N).

^{11}B{^{1}H} NMR(128.5 MHz, toluene-*d8*): δ(ppm) = −1.2 (s, $\omega_{1/2}$= 3 Hz).

Elemental Analysis. Calcd. for $C_{15}H_{25}BN_2S_3$: C 52.93, H 7.40, N 8.23, S 28.26. Found: C 52.59, H 7.26, N 8.39, S 28.32.

MS (EI): m/z = 279.1 [M−SEt]$^+$.

IR (ATR, cm^{-1}): ν = 3060 (w), 2961 (w), 2928 (w), 2916 (w), 1485 (m), 1453 (m), 1439 (m), 1382 (m), 1350 (w), 1326 (w), 1255 (m), 1194 (w), 1139 (w), 1053 (w), 1020 (w), 975 (w), 926 (w), 833 (s), 818 (s), 728 (s), 667 (m), 602 (m).

BAC-B(SEt)$_2$CN (2C).

BAC-B(SEt)$_3$ (**1C**, 2 g, 5.86 mmol) was dissolved in toluene (20 mL) and Me_3SiCN (581 mg, 5.86 mmol) in toluene (5 mL) was added dropwise. After stirring the mixture for 24 h at 45 °C the solvent was evaporated to dryness. The solid residue was dissolved in CH_2Cl_2 and purified by silica pad filtration. The product (1.41 g, 5.0 mmol, 86%) was obtained as a pale yellow solid after drying *in vacuo*. Crystals suitable for X-ray crystallography were obtained by slow diffusion of pentane into a solution of the product in CH_2Cl_2.

^{1}H NMR (400 MHz, C_6D_6): δ(ppm) = 6.93-6.89 (m, 2 H, ar-H), 6.56-6.52 (m, 2 H, ar-H), 3.87 (s, 6 H, NCH_3), 3.03-2.94 (m, 2H, SCH_2CH$_3$), 2.91-2.82 (m, 2 H, SCH_2CH3)$_3$), 1.35 (t, $^3J_{HH}$ = 7 Hz, 6 H, SCH$_2$CH_3).

^{13}C{^{1}H} NMR (100 MHz, C_6D_6): δ(ppm) = 132.7 (s, ar-*C*N), 124.8 (s, ar-*C*H), 111.2 (s, ar-*C*H), 33.6 (s, N*C*H$_3$), 24.4 (s, S*C*H$_2$CH$_3$), 17.3 (s, SCH$_2$*C*H$_3$), not observed (B*C*N).

^{11}B{^{1}H} NMR (96.3 MHz, C_6D_6): δ(ppm) = −13.72 (s, $\omega_{1/2}$= 3 Hz).

Elemental Analysis. Calcd. for $C_{14}H_{20}BN_3S_2$: C 55.08, H 6.60, N 13.77, S 21.00. Found: C 54.81, H 6.62, N 14.00, S 21.00.

MS (EI): m/z = 305.1 $[M]^+$, 244.1 $[M{-}SEt]^+$.

IR (ATR, cm^{-1}): ν = 3040 (w), 2982 (m), 2970 (m), 2922 (m), 2188 (w), 1480 (m), 1466 (s), 1429 (w), 1390 (s), 1372 (m), 1263 (m).

BAC-B(CN)$_2$SEt (3C).

Me_3SiCN (3.54 g, 35.7 mmol) was added to a solution of BAC-B(SEt)$_3$ (**1C**, 5.3 g, 15.6 mmol) in toluene (80 mL). Subsequently B(SEt)$_3$ (100 mg, 0.52 mmol) was added. The mixture was stirred for 12 h at 95 °C. The product precipitated as a yellow solid and was separated from the supernatant solution by filtration. The remaining solid was washed with pentane and dried *in vacuo* to afford the product (3.26 g, 12.1 mmol, 77%).

^{1}H NMR (400 MHz, CD_2Cl_2) δ(ppm) = 7.66-7.60 (m, 4 H, Ar-*H*), 4.30 (s, 6 H, NCH_3), 2.72-2.65 (m, 2 H, SCH_2CH$_3$), 1.33 (t, $^3J_{HH}$= 7.4 Hz, 2 H, SCH$_2$$CH_3$).

^{13}C{^{1}H} NMR (100 MHz, CD_2Cl_2) (ppm) = 133.6 (s, ar-C), 128.0 (q, $^1J_{BC}$ = 70 Hz, B*C*N), 126.8 (s, ar-*C*H), 112.4 (s, ar-*C*H), 34.4 (s, N*C*H$_3$), 24.6 (s, SCH_2CH$_3$), 16.9 (s, SCH$_2$$CH_3$), not observed (carbene-B*C*N).

^{11}B{^{1}H} NMR(128.5 MHz, CD_2Cl_2): δ(ppm) = −24.7 (s, $\omega_{½}$= 2 Hz).

Elemental Analysis. Calcd. for $C_{13}H_{15}BN_4S$: C 57.80, H 5.60, N 20.74, S 11.87. Found: C 57.65, H 5.55, N 20.49, S 11.67.

MS (EI): m/z: 270.1 $[M]^+$, 209.1 $[M-SEt]^+$.

IR (ATR, cm^{-1})**:** υ = 2979 (w), 2202 (w), 2127 (w), 1493 (w), 1471 (s), 1395 (m), 1383 (m), 1350 (w), 1262 (m), 1253 (m), 1133 (w), 1065 (w), 1018 (m), 1008 (m), 940 (m), 912 (s), 829 (w), 808 (m), 763 (s), 758 (s), 692 (m), 665 (m).

BAC-B(CN)$_2$I (7C).

Methyl iodide (2.8 mL, 45.0 mmol) was added to a solution of BAC-B(CN)$_2$SEt (**3C**, 2.0 g, 7.4 mmol) in chloroform (40 mL). The resulting yellow mixture was stirred for 5 h at 70 °C. After cooling to r.t. the suspension was filtered and the residual pale yellow solid dried *in vacuo* (1.84 g, 5.5 mmol, 74%). Crystals suitable for X-ray crystallography were obtained by slow diffusion of pentane into a solution of the product in CH_2Cl_2.

^{1}H NMR (600 MHz, CD_2Cl_2) δ(ppm) = 7.69-7.64 (m, 4 H, ar-*H*), 4.24 (s, 6 H, NCH_3).

^{13}C{^{1}H} NMR (150 MHz, CD_2Cl_2) δ(ppm) = 133.6 (s, ar-*C*N), 127.2 (s, ar-*C*H), 112.5 (s, ar-*C*H), 34.6 (s, N*C*H$_3$), not observed (B*C*N).

^{11}B{^{1}H} NMR (96.3 MHz, CD_2Cl_2): δ(ppm) = −41.2 (s, $\omega_{½}$ = 30 Hz).

Elemental Analysis. Calcd. for $C_{11}H_{10}BIN_4$: C 39.33, H 3.00, N 16.68. Found: C 39.02, H 3.05, N 16.50.

MS (EI): m/z: 335.9 [M]$^+$, 209.9 [M−I]$^+$.

IR (ATR, cm^{-1}): υ = 1473 (m), 1394 (w), 1354 (w), 1255 (w), 1207 (w), 1134 (w), 1020 (m), 1009 (w), 989 (w), 959 (w), 942 (w), 919 (m), 803 (w), 792 (w), 753 (s), 734 (w), 720 (m), 669 (w), 663 (w), 651 (w), 605 (w), υ (CN) not observed.

K[BAC-B(CN)$_2$] (K-8C).

BAC-B(CN)$_2$I (**7C**, 250 mg, 0.7 mmol) was added to a solution of KC_8 (604 mg, 4.5 mmol) in DME (evaporated to dryness *in vacuo*. The product was obtained as a bright-orange solid (139 mg, 0.56 mmol, 80%).

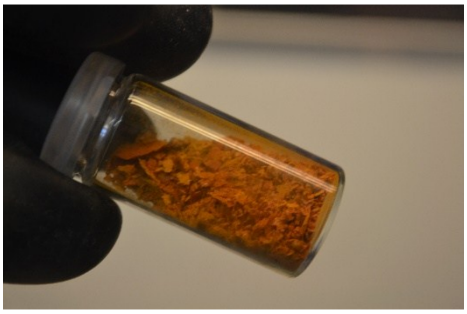

18-crown-6 was added to increase the solubility of **K-8C** and to access further analysis. Crystals suitable for X-ray crystallography were obtained by slow diffusion of pentane into a mixture of 18-crown-6 and **K-8C** in THF to afford single crystals of [K(18-cr-6)][BAC-B(CN)$_2$], **[K(18-cr-6)]-8C.**

^{1}H NMR (300 MHz, THF-*d8*) δ(ppm) = 6.35-6.32 (m, 2 H, ar-*H*), 6.12-6.09 (m, 2 H, ar-*H*), 3.55 (s, 60 H, 18-crown-6 CH_2).

^{13}C{^{1}H} NMR (75 MHz, THF-*d8*) δ(ppm) = 139.5 (s, ar-*C*N), 117.8 (s, ar-*C*H), 101.0 (s, ar-*C*H), 70.9 (s, 18-cr-6 *C*H$_2$), 32.3 (s, N*C*H$_3$), not observed (B*C*N).

^{11}B{^{1}H} NMR (96.3 MHz, THF-*d8*): δ(ppm) = −24.1 (s, $\omega_{½}$ = 20 Hz).

MS: Representative mass spectra could not be recorded.

Experimental IR of **[K(18-cr-6)]-8C**, THF solution, cm^{-1}: υ = 2134 (s), 2115 (m).

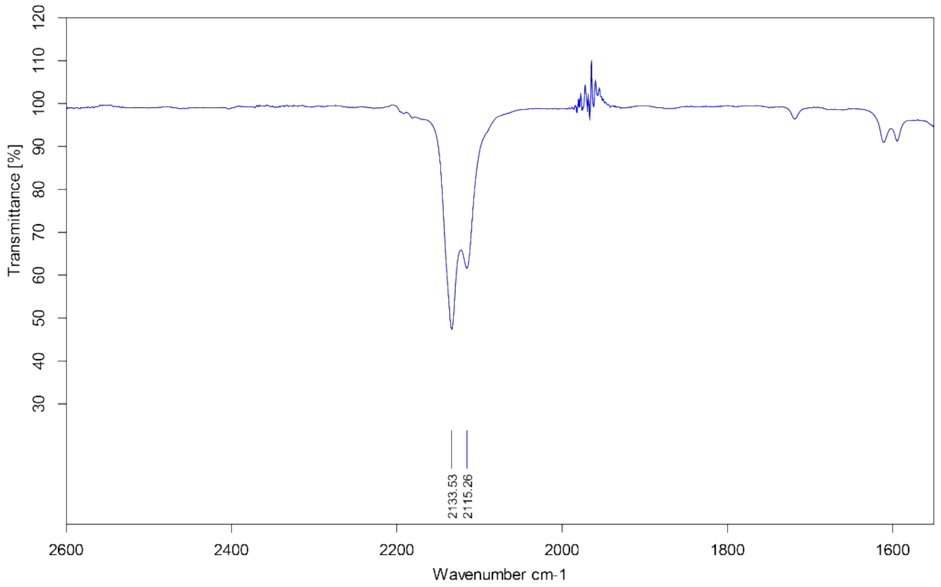

Figure 7: Experimental IR spectrum of ***[K(18-cr-6)]-8C****.*

Calculated IR spectrum of **8C**, cm^{-1}: υ = 2110 (s), 2085 (m).

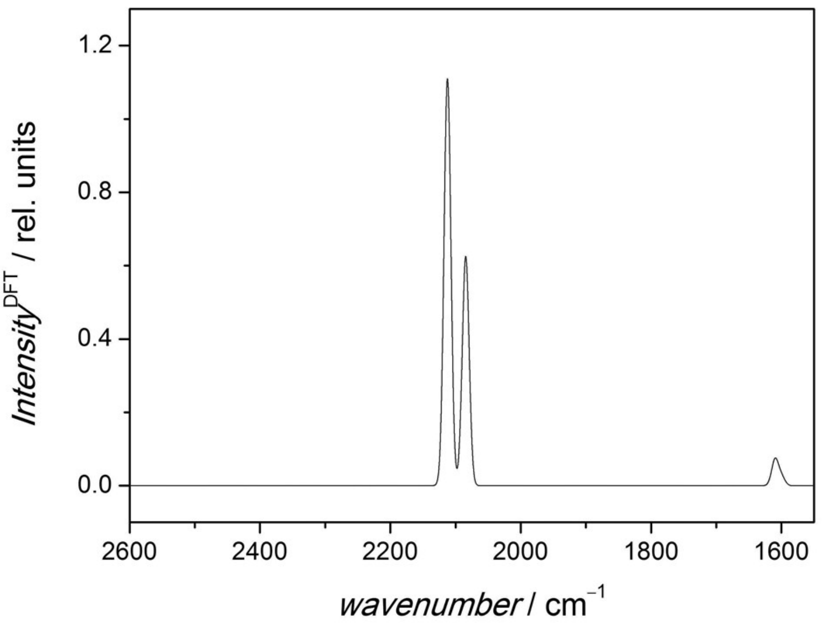

Figure 8: Calculated IR spectrum of anion ***8C****.*

BAC-B(CN)$_2$Me (11).

Methyl iodide (0.2 mL, 3.2 mmol) was added to a suspension of **K-8C** (400 mg, 1.6 mmol) in THF (5 mL). After 10 min the reaction mixture was dried *in vacuo*, the residue was suspended in H_2O and the solution extracted three times with CH_2Cl_2. The combined organic phases were dried over Na_2SO_4 and the solvent was removed *in vacuo*. The residue was dissolved in CH_2Cl_2 and filtered through Celite®545. After evaporation of the filtrate to dryness BAC-B(CN)$_2$Me (233 mg, 1.04 mmol, 65%) was obtained. Crystals suitable for X-ray crystallography were obtained by slow diffusion of pentane into a solution of the product in CH_2Cl_2.

^{1}H NMR (600 MHz, CD_2Cl_2) δ(ppm) = 7.64-7.62 (m, 2 H, ar-*H*), 7.59-7.58 (m, 2 H, ar-*H*), 4.20 (s, 6 H, NC*H*$_3$), 0.37 (s, 3 H, BC*H*$_3$).

^{13}C{^{1}H} NMR (150 MHz, CD_2Cl_2) δ(ppm) = 133.5 (s, ar-*C*N), 126.3 (s, ar-*C*H), 112.1 (s, ar-CH), 34.0 (s, B*C*H$_3$), not observed (B*C*N).

^{11}B{^{1}H} NMR (96.3 MHz, CD_2Cl_2): δ(ppm) = −27.4 (s, $\omega_{½}$ = 18 Hz).

MS (EI): m/z: 224.1 [M]$^+$, 209.1 [M−CH$_3$]$^+$.

IR (ATR, cm^{-1}): υ = 3067 (w), 3040 (w), 2950 (w), 2903 (w), 2202 (w), 1473 (m), 1400 (w), 1354 (w), 1301 (w), 1261 (w), 1203 (w), 1140 (w), 1041 (w), 1016 (m), 1005 (m), 989 (m), 946 (w), 863 (w), 818 (m), 761 (s), 757 (s), 624 (w).

Elemental Analysis. Calcd. for $C_{12}H_{13}BN_4 \cdot 0.11\ CH_2Cl_2$ C 62.33, H 5.67, N 24.02. Found: C 62.49, H 5.72, N 23.68.

BAC-B(CN)$_2$SiMe$_3$ (12).

An excess of Me$_3$SiCl (0.1 mL, 0.8 mmol) was added to a suspension of **K-8C** (60 mg, 0.2 mmol) in THF (5 mL) and the mixture was stirred for 15 min. After removing the solvent *in vacuo,* the residue was dissolved in CH$_2$Cl$_2$, filtrated ovthroughr Celite®545, and the filtrate was evaporated to dryness *in vacuo*. The residue was recrystallized by slow diffusion of pentane into a solution of the solid in CH$_2$Cl$_2$ to afford the product (31 mg, 0.1 mmol, 54%).

^{1}H NMR (400 MHz, CD$_2$Cl$_2$) δ(ppm) = 7.58-7.52 (m, 4 H, ar-*H*), 4.12 (s, 6 H, NC*H$_3$*), 0.14 [s, 9 H, Si(C*H*$_3$)$_3$].

^{13}C{^{1}H} NMR (100 MHz, CD$_2$Cl$_2$) δ(ppm) = 133.7 (s, ar-*C*N), 125.8 (s, ar-*C*H), 111.5 (s, ar-*C*H), 34.2 (s, N*C*H$_3$), -1.0 [s, Si(CH$_3$)$_3$], not observed (B*C*N).

^{11}B{^{1}H} NMR (96.3 MHz, CD$_2$Cl$_2$): δ(ppm) = −33.3 (s, $\omega_{½}$ = 27 Hz).

Elemental Analysis. Calcd. for C$_{14}$H$_{19}$BN$_4$Si: C 59.58, H 6.79, N 19.85. Found: C 59.62, H 6.90, N 19.68.

MS (EI): m/z: 282.1 [M]$^+$, 267.1 [M−Me]$^+$, 209.1 [M−Si(Me)$_3$]$^+$.

IR (ATR, cm^{-1}): υ = 2948 (w), 2892 (w), 2192 (m), 1965 (w), 1496 (w), 1471 (m), 1421 (w), 1395 (w), 1349 (w), 1259 (w), 1239 (m), 1203 (w), 1168 (w), 1127 (w), 1022 (m), 1017 (m), 955 (w), 929 (m), 830 (s), 751 (s), 737 (s), 690 (m), 683 (m), 665 (m), 624 (m), 612 (m).

BAC-B(CN)$_2$SnMe$_3$ (13).

Me_3SnCl (233 mg, 1.2 mmol) was added to a suspension of **K-8C** (290 mg, 1.2 mmol) in THF (10 mL) and the mixture was stirred for 15 min. The solvent was removed *in vacuo*, the solid residue dissolved in CH_2Cl_2 (25 mL) and filtered. Crystallization by slow diffusion of pentane into the solution of CH_2Cl_2 afforded the product (224 mg, 0.6 mmol, 50%). Crystals suitable for X-ray crystallography were obtained by slow diffusion of pentane into a solution of the product in CH_2Cl_2.

^{1}H NMR (600 MHz, CD_2Cl_2) δ(ppm) = 7.54-7.50 (m, 4 H, ar-*H*), 4.06 (s, 6 H, NCH_3), 0.15 [s, 9 H, Sn(CH_3)$_3$].

^{13}C{^{1}H} NMR (150 MHz, CD_2Cl_2) δ(ppm) = 133.6 (s, ar-*C*N), 129.5 (q, $^1J_{BC}$ = 71 Hz, B*C*N), 125.7 (s, ar-*C*H), 111.3 (s, ar-*C*H), 33.9 (s, N*C*H$_3$), -9.1 [s, Sn(CH$_3$)$_3$], not observed (carbene-B*C*N).

^{11}B{^{1}H} NMR (96.3 MHz, CD_2Cl_2): δ(ppm) = −35.9 [satellites: d, $^1J(^{117}Sn\text{-}^{11}B)$ = 325 Hz; d, $^1J(^{119}Sn\text{-}^{11}B)$ = 338 Hz, $\omega_{1/2}$ = 6 Hz].

Elemental Analysis. Calcd. for $C_{14}H_{19}BN_4Sn$: C 45.10, H 5.14, N 15.03. Found: C 45.17, H 5.18, N 14.74.

MS (EI): m/z: 374.1 [M]$^+$, 209.1 [M−Sn(CH$_3$)$_3$]$^+$.

IR (ATR, cm^{-1}): υ = 2971 (w), 2909 (w), 2188 (m), 1604 (w), 1493 (w), 1471 (m), 1421 (w), 1397 (w), 1346 (w), 1261 (w), 1204 (w), 1193 (w), 1168 (w), 1126 (w), 1023 (m), 1017 (m), 962 (w), 938 (m), 802 (w), 753 (s), 724 (m), 650 (w), 612 (w).

BAC-B(CN)$_2$P(*t*Bu)$_2$ (14).

ClP(*t*Bu)$_2$ (0.13 mL, 0.69 mmol) was added to a solution of **K-8C** (230 mg, 0.93 mmol) in DME (20 mL). After stirring for 1 h the mixture was filtered and evaporated to dryness. The remaining solids were washed with pentane (4×5 mL) and dried *in vacuo* (132 mg, 0.37 mmol, 40%). Crystals suitable for X-ray crystallography were obtained by slow diffusion of pentane in a solution of the product in CH_2Cl_2.

^{1}H NMR (400 MHz, THF-d$_8$) δ(ppm) = 7.83-7.55 (m, 4 H, ar-*H*), 4.45 (s, 6 H, NC*H*$_3$), 1.26 [d, $^3J_{PH}$ = 11 Hz, 18 H, C(C*H*$_3$)$_3$].

^{13}C{^{1}H} NMR (150 MHz, THF-d$_8$) δ(ppm) = 134.3 (s, ar-*C*N), 129.8 (q, $^1J_{\mathrm{BC}}$ = 70 Hz, B*C*N), 126.3 (s, ar-*C*H), 112.7 (s, ar-*C*H), 34.6 (d, $^2J_{\mathrm{PC}}$ = 14 Hz, N*C*H$_3$), 33.0 (d, $^2J_{\mathrm{PC}}$ = 26.3 Hz, *C*(CH$_3$)$_3$), 32.1 [d, $^2J_{\mathrm{PC}}$ = 13 Hz, C(*C*H$_3$)$_3$], not observed (carbene-B*C*N).

^{11}B{^{1}H} NMR (128.5 MHz, THF-d$_8$): δ(ppm) = −29.6 [d, $^1J(^{31}P\text{-}^{11}B)$ = 24 Hz, $\omega_{½}$ = 9 Hz].

^{31}P{^{1}H} NMR (162.1 MHz, THF-d$_8$): δ(ppm) = 5.96 [q, $^1J(^{31}P\text{-}^{11}B)$ = 24 Hz].

Elemental Analysis. Calcd. for $C_{19}H_{28}BN_4P$: C 64.42, H 7.97, N 15.82. Found: C 64.47, H 7.86, N 15.94.

MS (ESI, positive mode): 377.2 [M+Na]$^+$.

IR (ATR, cm^{-1}): $\tilde{\nu}$ = 3061 (w), 2918 (w), 2892 (w), 2858 (w), 2197 (w), 1604 (w), 1470 (s), 1390 (m), 1361 (m), 1309 (m), 1259 (w), 1231 (w), 1200 (w), 1171 (w), 1221 (w), 1059 (w), 1016 (s), 983 (w), 947 (w), 882 (w), 853 (w), 806 (m), 747 (s), 662 (w), 645 (w).

Synthesis of IMe(Me)$_2$-substituted compounds

IMe(Me)$_2$-B(SEt)$_3$, (1A).

B(SEt)$_3$ (1.56 g, 8.1 mmol) was added dropwise to a solution of IMe(Me)$_2$ (1 g, 8.1 mmol) in toluene. After stirring the pale yellow solution for 5 min the solvent was removed *in vacuo*. The residue was washed with pentane (40 mL), and after evaporating to dryness *in vacuo* the product was obtained as a colorless powder (2.57 g, 8.0 mmol, 99%).

^{1}H NMR (400 MHz, C_6D_6) δ(ppm) = 3.85 (s, 6 H, NCH_3), 2.92-2.86 (m, 6 H, SCH_2), 1.43 (t, $^3J_{HH}$ = 7.5 Hz, 9 H, SCH$_2$CH_3), 1.11 (s, 6 H, NCCH_3).

^{13}C{^{1}H} NMR (100 MHz, C_6D_6) δ(ppm) = 125.0 (s, N*CC*N), 34.4 (s, N*C*H$_3$) , 23.9 (s, S*C*H$_2$), 17.8 (s, SCH$_2$*C*H$_3$), 8.2 (s, NC*C*H$_3$), not observed (B*C*N).

^{11}B{^{1}H} NMR (96.3 MHz, C_6D_6): δ(ppm) = -1.1 (s, $\omega_{½}$ = 5 Hz).

Elemental Analysis. Calcd. for $C_{13}H_{27}BN_2S_3$: C 49.05, H 8.55, N 8.80, S 30.21. Found: C 49.37, H 8.64, N 9.21, S 29.71.

MS (EI): m/z = 289.1 [M-Et]$^+$, 257.1 [M-SEt]$^+$.

IR (ATR cm^{-1}): υ = 2957 (m), 2923 (m), 2859 (m), 1657 (m), 1438 (s), 1404 (w), 1384 (s), 1370 (m), 1258 (m).

IMe(Me)$_2$-B(CN)(SEt)$_2$ (2A).

Me_3SiCN (164 mg, 1.65 mmol) dissolved in toluene (2 mL) was added dropwise to a solution of IMe(Me)$_2$-B(SEt)$_3$ (500 mg, 1.57 mmol) in toluene (5 mL). The pale yellow solution was left for 24 h at 45 °C, and the solvent was removed *in vacuo*. After washing with pentane (40 mL) the product was obtained as a colorless powder (442 mg, 1.55 mmol, 99%).

^{1}H NMR (400 MHz, C_6D_6) δ(ppm) = 3.60 (s, 6 H, NCH_3), 3.04-2.84 (m, 4 H, SCH_2CH$_3$), 1.40 (t, $^3J_{HH}$ = 8 Hz, 6H, SCH$_2$CH_3), 1.11 (s, 6 H, NCCH_3).

^{13}C{^{1}H} NMR (100 MHz, C_6D_6) δ(ppm) = 125.7 (s, N*CC*N), 34.0 (s, N*C*H$_3$), 24.6 (s, S*C*H$_2$), 17.4 (s, SCH$_2$*C*H$_3$), 7.7 (s, NC*C*H$_3$), not observed (B*C*N).

^{11}B{^{1}H} NMR (96.3 MHz, C_6D_6): δ(ppm) = -13.6 (s, $\omega_{½}$ = 7 Hz).

Elemental Analysis. Calcd. for $C_{12}H_{22}BN_3S_2$: C 50.88, H 7.83, N 14.83, S 22.64. Found: C 50.68, H 7.77, N 15.10, S 22.20.

MS (EI): m/z = 283.1 [M]$^+$, 222 [M-SEt]$^+$.

IR (ATR cm^{-1}): υ = 2970 (m), 2923 (m), 2868 (m), 2181 (w), 1655 (m), 1471 (m), 1442 (s), 1413 (w), 1393 (s), 1371 (m), 1260 (s), 1227 (w).

IMe(Me)$_2$-B(CN)$_2$SEt (3A).

Me_3SiCN (954 mg, 9.61 mmol) dissolved in toluene (2 mL) was added dropwise to a solution of IMe(Me)$_2$-B(SEt)$_3$ (1.33 g, 4.18 mmol) in toluene (10 mL). Subsequently B(SEt)$_3$ (30 mg, 0.15 mmol) was added. After stirring the pale yellow solution for 12 h at 95 °C and removal of the solvent *in vacuo* a yellow solid was obtained. The residue was dissolved in a minimum amount of CH_2Cl_2 and passed through a pad of silica gel. Evaporating to dryness *in vacuo* afforded the product as a colorless crystalline material (853 mg, 3.43 mmol, 82%).

^{1}H NMR (400 MHz, C_6D_6) δ(ppm) = 3.30 (s, 6 H, NC*H*$_3$), 3.08-3.02 (m, 2 H, SC*H*$_2$CH$_3$), 1.49 (t, $^3J_{HH}$ = 7 Hz, 3 H, SCH$_2$C*H*$_3$), 0.94 (s, 6 H, NCC*H*$_3$).

^{13}C{^{1}H} NMR (100 MHz, C_6D_6) δ(ppm) = 126.1 (s, N*CC*N), 33.5 (s, N*C*H$_3$), 24.7 (s, S*C*H$_2$), 17.1 (s, SCH$_2$*C*H$_3$), 7.8 (s, NC*C*H$_3$), not observed (B*C*N).

^{11}B{^{1}H} NMR (96.3 MHz, C_6D_6): δ(ppm) = -24.4 (s, $\omega_{½}$ = 6 Hz).

Elemental Analysis. Calcd. for $C_{11}H_{17}BN_4S$: C 53.24, H 6.91, N 22.58, S 12.92. Found: C 53.13, H 6.85, N 22.65, S 12.18.

MS (EI): m/z = 248.1 [M]$^+$, 187.1 [M-SEt]$^+$.

IR (ATR cm^{-1}): υ = 2961 (m), 2924 (m), 2869 (m), 2204 (w), 1653 (m), 1487 (m), 1446 (s), 1422 (m), 1403 (m), 1374 (m), 1263 (m), 1232 (w), 916(s), 895(s), 797(s), 681(s).

IMe(Me)$_2$-B(CN)$_2$I (7A).

IMe(Me)$_2$-B(CN)$_2$(SEt) (3.20 g, 12.89 mmol) was dissolved in toluene (70 mL) and methyl iodide (3,85 g, 27.09 mmol) was added. The mixture was stirred at 45 °C overnight, whereby the colorless product precipitated. Filtration, washing with pentane (40 mL) and drying *in vacuo* afforded a colorless crystalline material (2.39 mg, 7.61 mmol, 59%).

^{1}H NMR (300 MHz, CD_2Cl_2) δ(ppm) = 3.83 (s, 6 H, NCH_3), 2.20 (s, 6 H, NCCH_3).

^{13}C{^{1}H} NMR (100 MHz, CD_2Cl_2) δ(ppm) = 128.3 (s, N*CC*N), 34.6 (s, N*C*H$_3$), 9.3 (s, NC*C*H$_3$), not observed (B*C*N).

^{11}B{^{1}H} NMR (96.3 MHz, CD_2Cl_2): δ(ppm) = -41.6 (s, $\omega_{½}$ = 47 Hz).

Elemental Analysis. Calcd. for $C_9H_{12}BIN_4$: C 34.43, H 3.85, N 17.85. Found: C 34.69, H 3.95, N 17.67.

MS (EI): m/z = 313.9 $[M]^+$.

IR (ATR cm^{-1}): υ = 2959 (m), 2929 (w), 2210 (w), 1643 (s), 1482 (s), 1441 (s), 1403 (m), 1062 (m), 990 (s), 898 (s), 749 (s), 642 (s).

General procedure for the reactions 2A-C → 3A-C.

In a typical reaction a portion of **2A-C** (5.00 mmol) was dissolved in toluene (50 mL) and Me_3SiCN (645 mg, 6.50 mmol) and $B(SEt)_3$ (30 mg, 0.15 mmol) were added. The solution was maintained at 95 °C for 24 h. The work-up of compounds **3A-C** was performed as mentioned above and afforded samples giving comparable yield and identical ^{1}H and ^{11}B NMR spectra as stated (*vide supra*).

Alternative preparation of K-8B and K-8C by reduction with K in liquid NH_3.

In a typical reaction a portion of potassium (234 mg, 6.00 mmol) was dissolved in liquid NH_3 (10 mL) at −60 °C. Compounds **7B** or **7C** (2.00 mmol) were added as solids to give a rapid change in color from blue to green. After 20 min all volatiles were removed *in vacuo* maintaining a constant temperature of −60 °C. The red (**K-8B**) or orange (**K-8C**) residue was dissolved (**K-8B:** THF, 20 mL; **K-8C**: DME, 20 mL) and the solution filtered to remove potassium iodide. The solvent was removed *in vacuo*, which afforded samples giving comparable yield and identical ^{1}H and ^{11}B NMR spectra as stated (*vide supra*).

NMR Spectra

IMes-substituted compounds

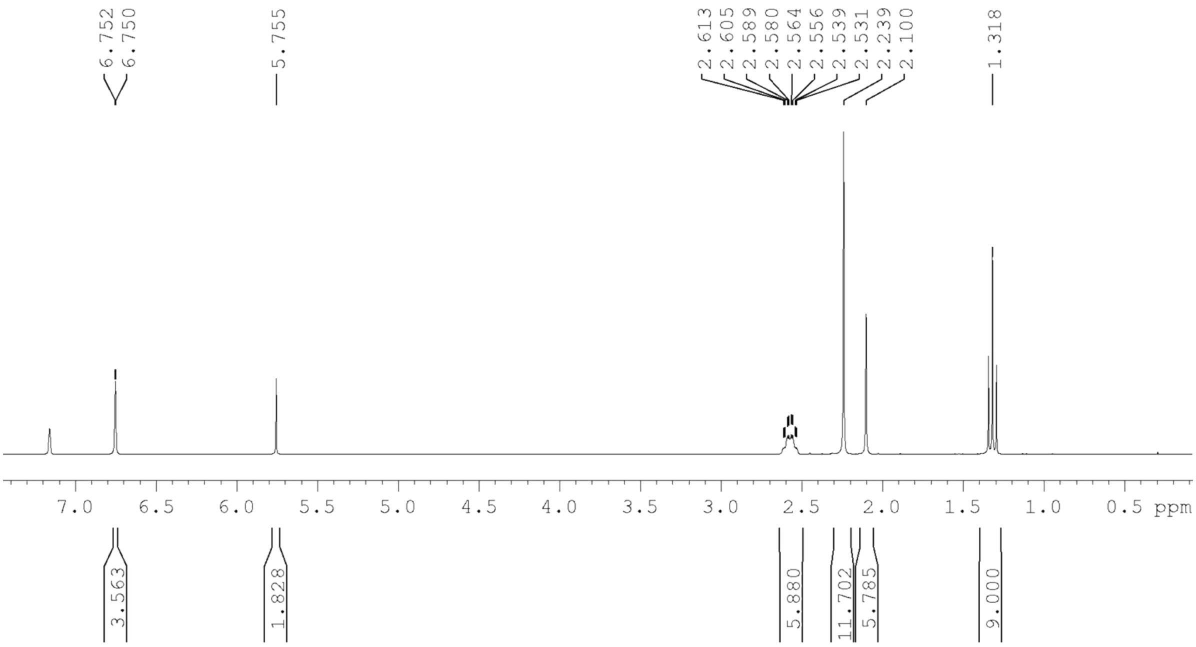

*Figure 9: ¹H NMR spectrum of IMes-B(SEt)₃, **1B** (300 MHz, C₆D₆, 25 °C).*

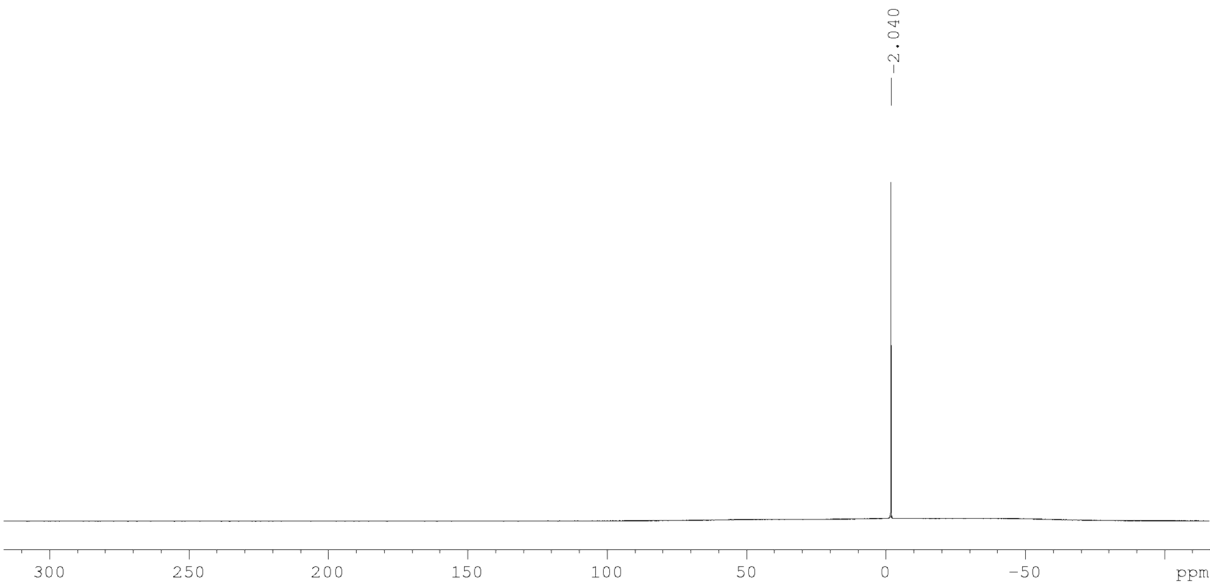

*Figure 10: ¹¹B{¹H} NMR spectrum of IMes-B(SEt)₃, **1B** (96.3 MHz, C₆D₆, 25 °C).*

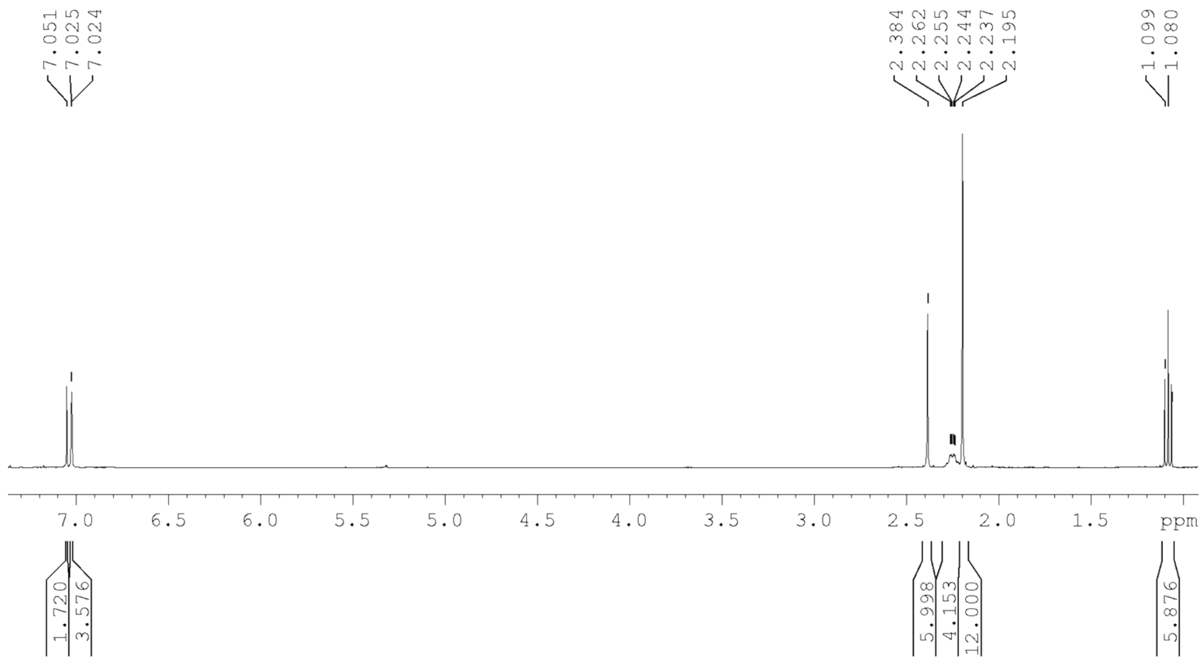

*Figure 11: ^{1}H NMR spectrum of IMes-B(CN)(SEt)$_2$, **2B** (400 MHz, CD_2Cl_2, 25 °C).*

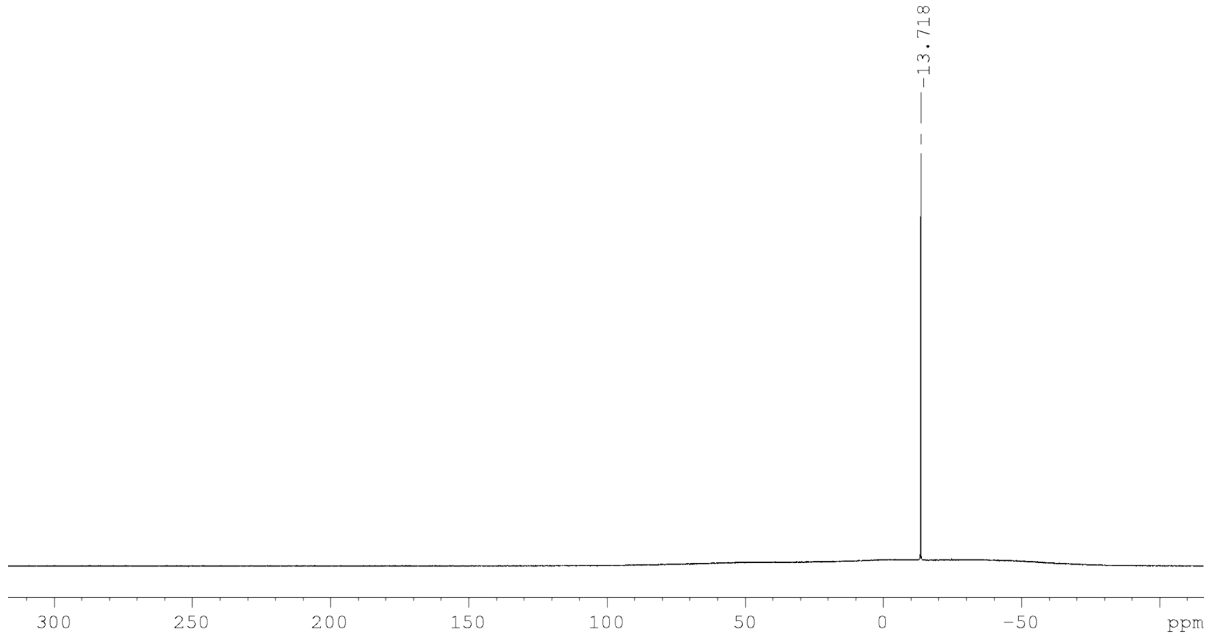

*Figure 12: $^{11}B\{^{1}H\}$ NMR spectrum of IMes-B(CN)(SEt)$_2$, **2B** (96.3 MHz, CD_2Cl_2, 25 °C).*

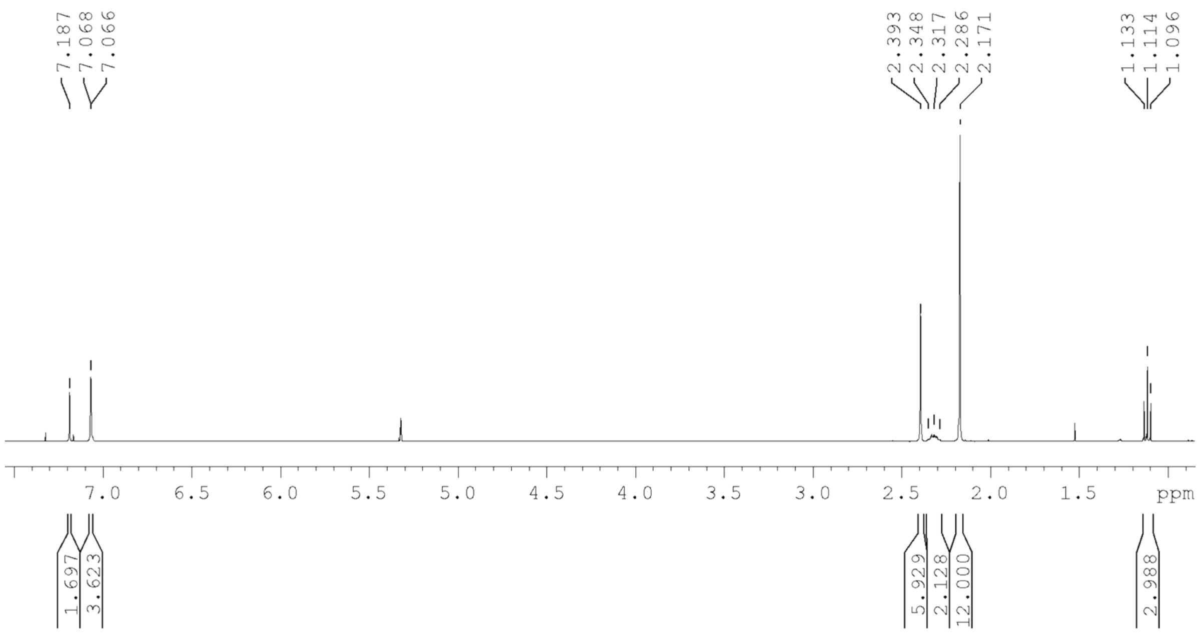

*Figure 13: ^{1}H NMR spectrum of IMes-B(CN)$_2$(SEt), **3B** (400 MHz, CD_2Cl_2, 25 °C).*

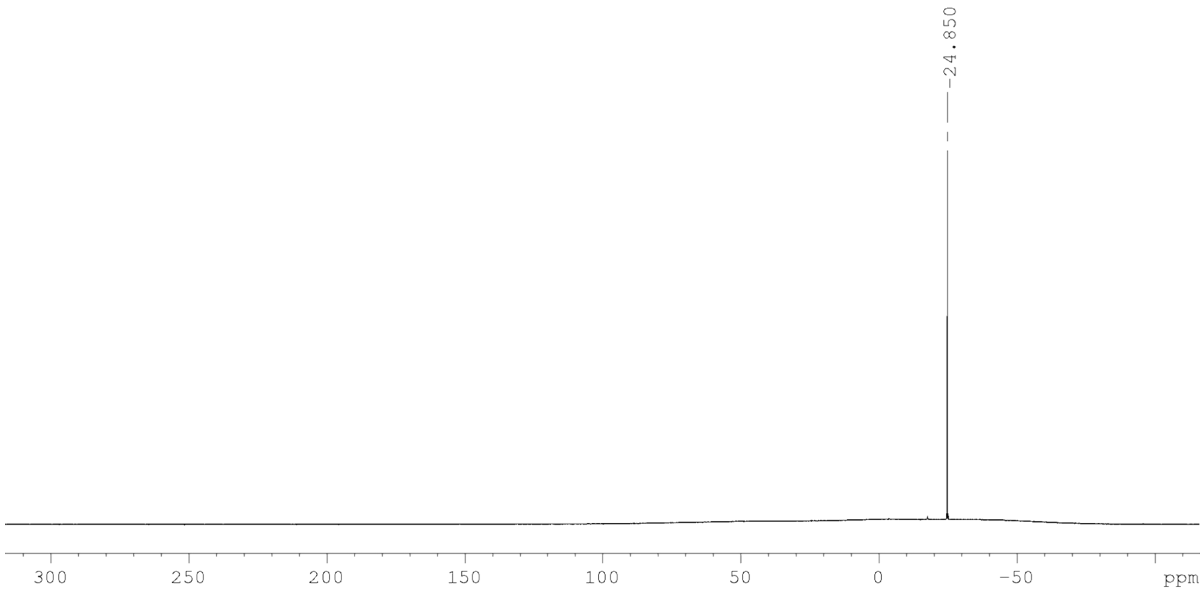

*Figure 14: $^{11}B\{^{1}H\}$ NMR spectrum of IMes-B(CN)$_2$(SEt), **3B** (96.3 MHz, CD_2Cl_2, 25 °C).*

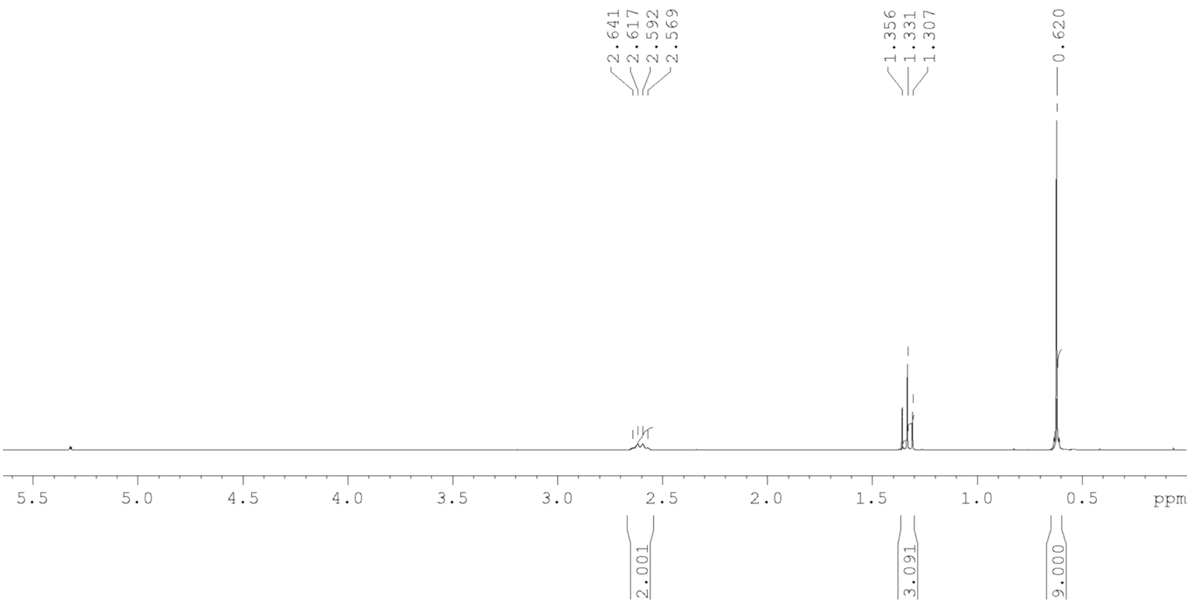

*Figure 15: ^{1}H NMR spectrum of TMS-N≡C-B(CN)$_2$SEt, **4** (300 MHz, CD_2Cl_2, 25 °C).*

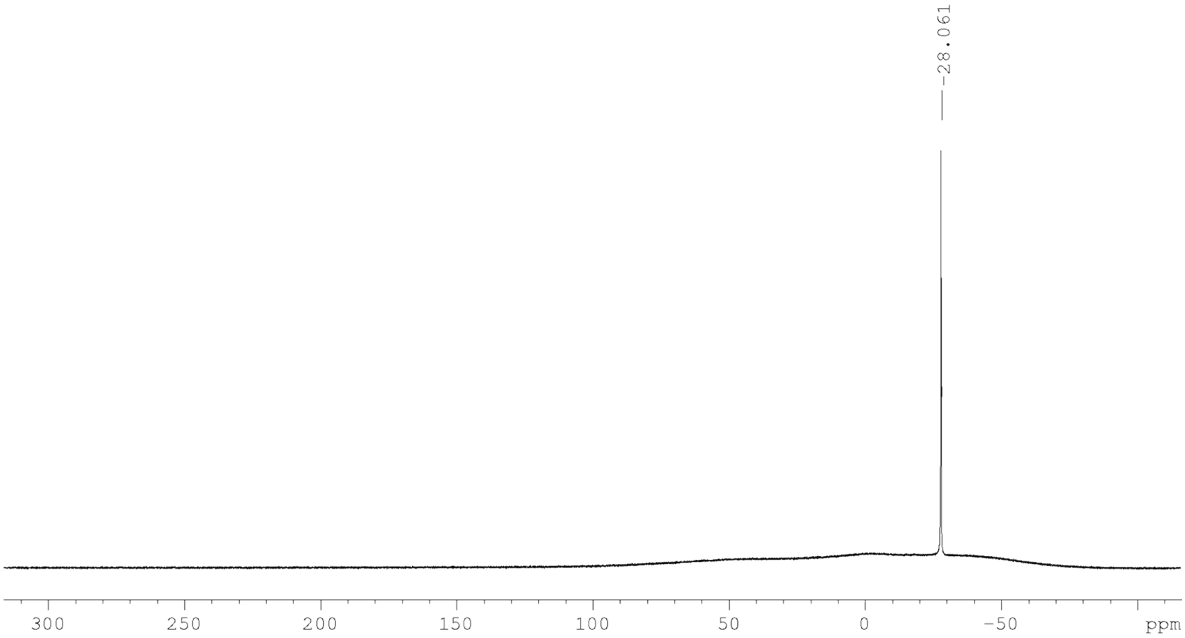

*Figure 16: $^{11}B\{^{1}H\}$ NMR spectrum of TMS-N≡C-B(CN)$_2$SEt, **4** (96.3 MHz, CD_2Cl_2, 25 °C).*

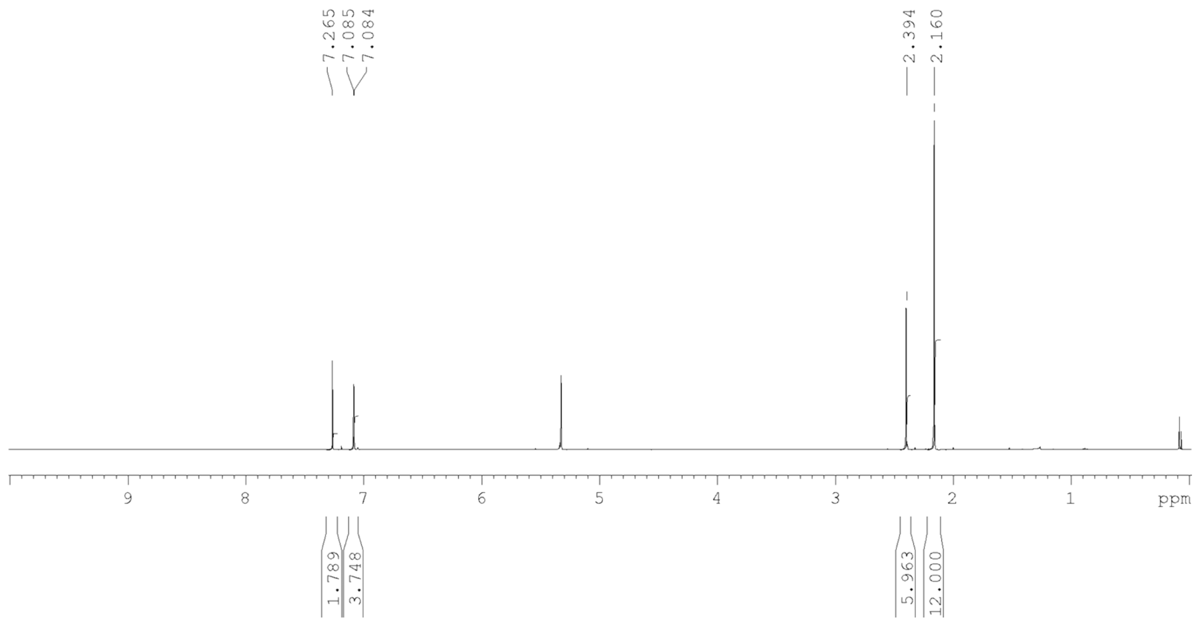

*Figure 17: ^{1}H NMR spectrum of IMes-B(CN)$_2$Br, **6** (400 MHz, CD_2Cl_2, 25 °C).*

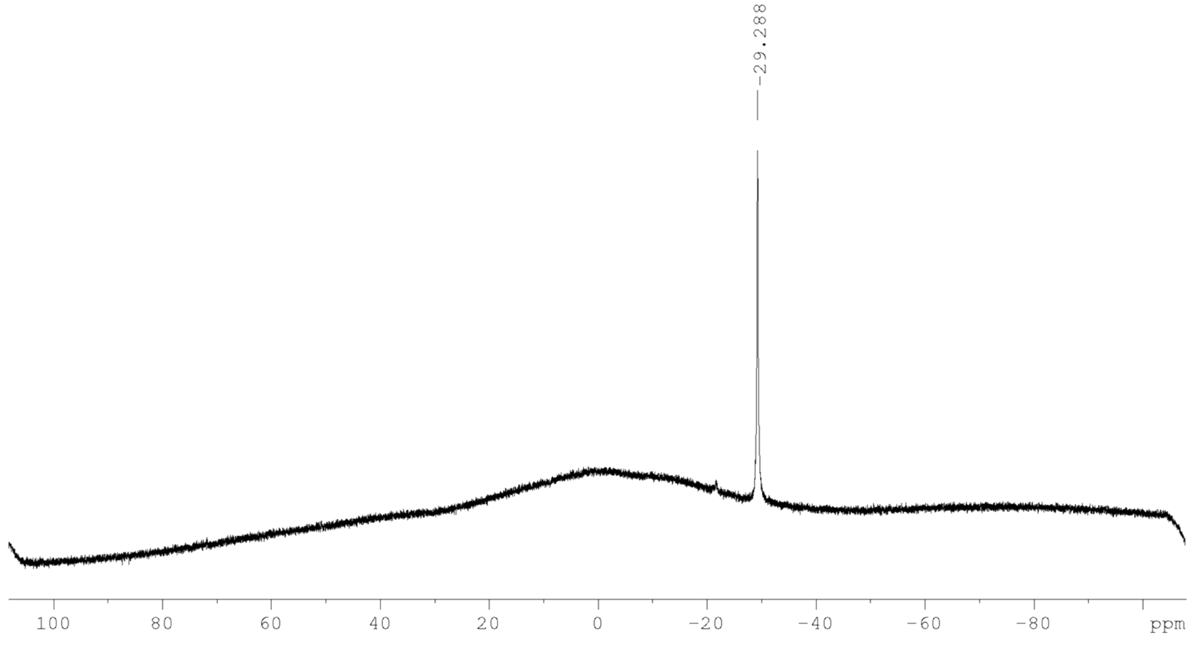

*Figure 18: $^{11}B\{^{1}H\}$ NMR spectrum of IMes-B(CN)$_2$Br, **6** (128.5 MHz, CD_2Cl_2, 25 °C).*

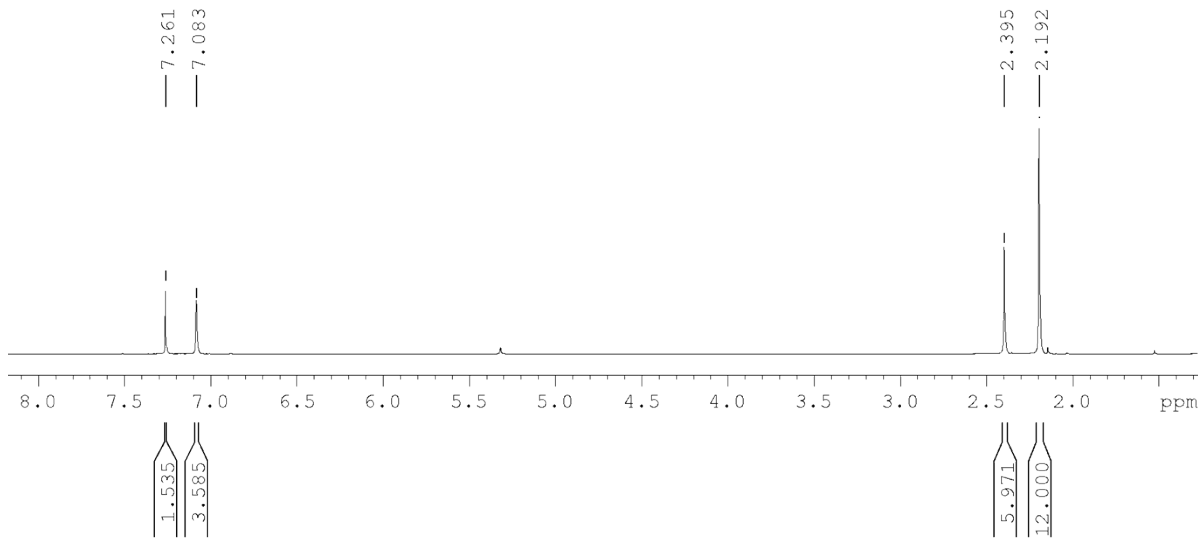

*Figure 19: ^{1}H NMR spectrum of IMes-$B(CN)_2I$, **7B** (400 MHz, CD_2Cl_2, 25 °C).*

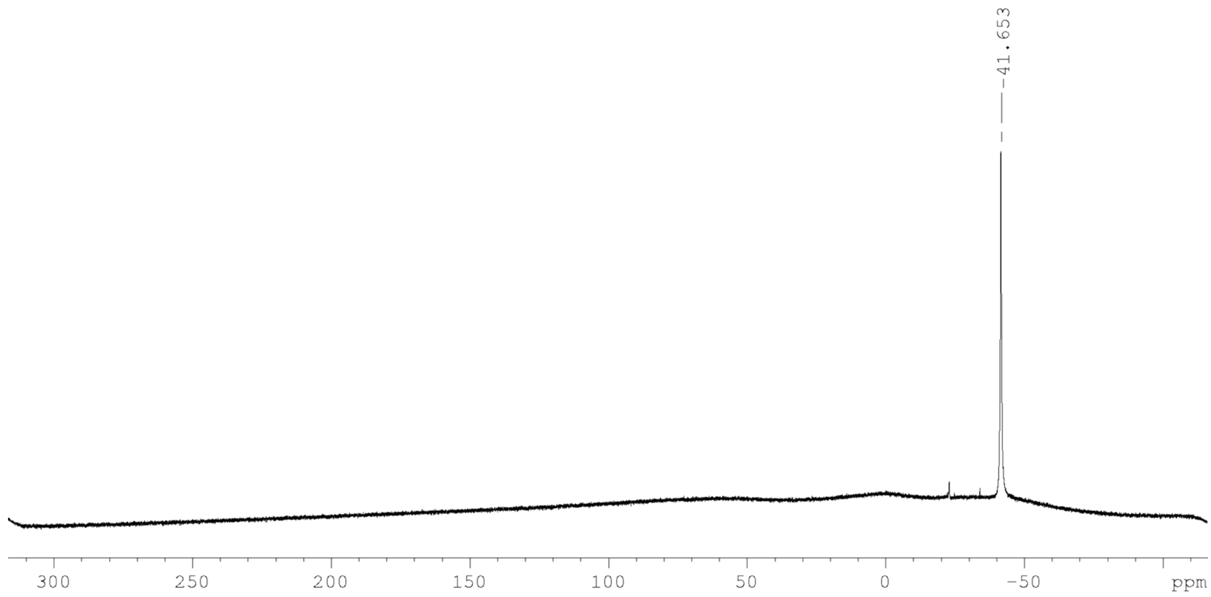

*Figure 20: $^{11}B\{^{1}H\}$ NMR spectrum of IMes-$B(CN)_2I$, **7B** (96.3 MHz, CD_2Cl_2, 25 °C).*

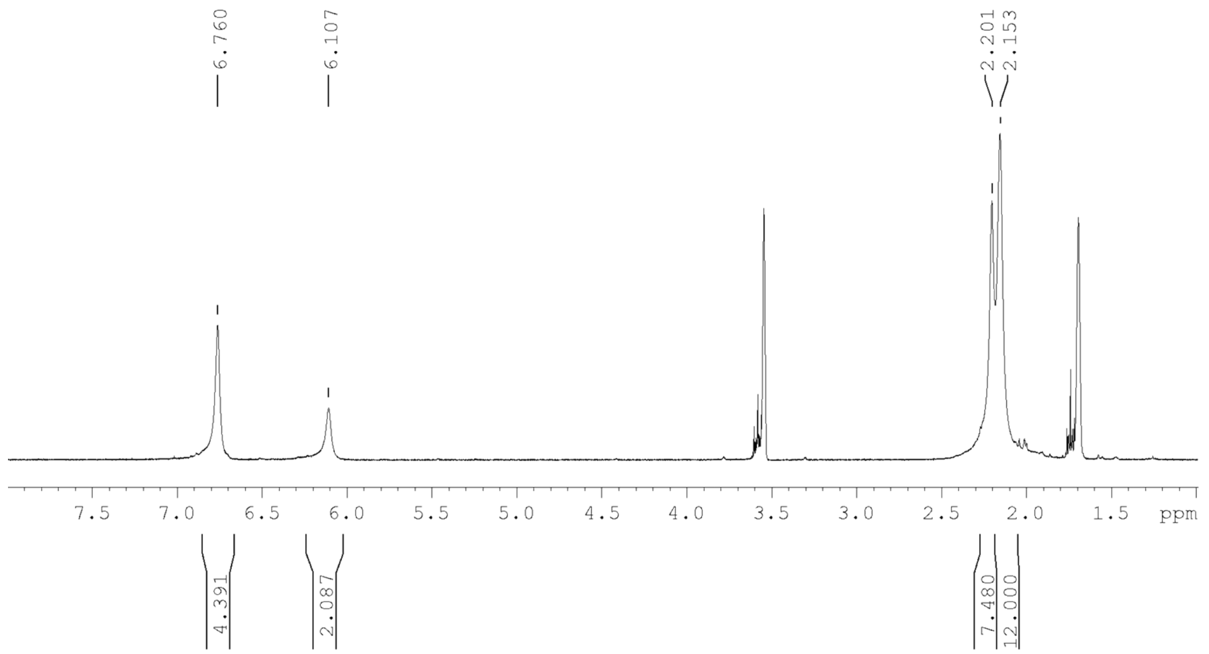

*Figure 21: ^{1}H NMR spectrum of K[IMes-B(CN)$_2$], **K-8B** (300 MHz, THF-d8, 25 °C).*

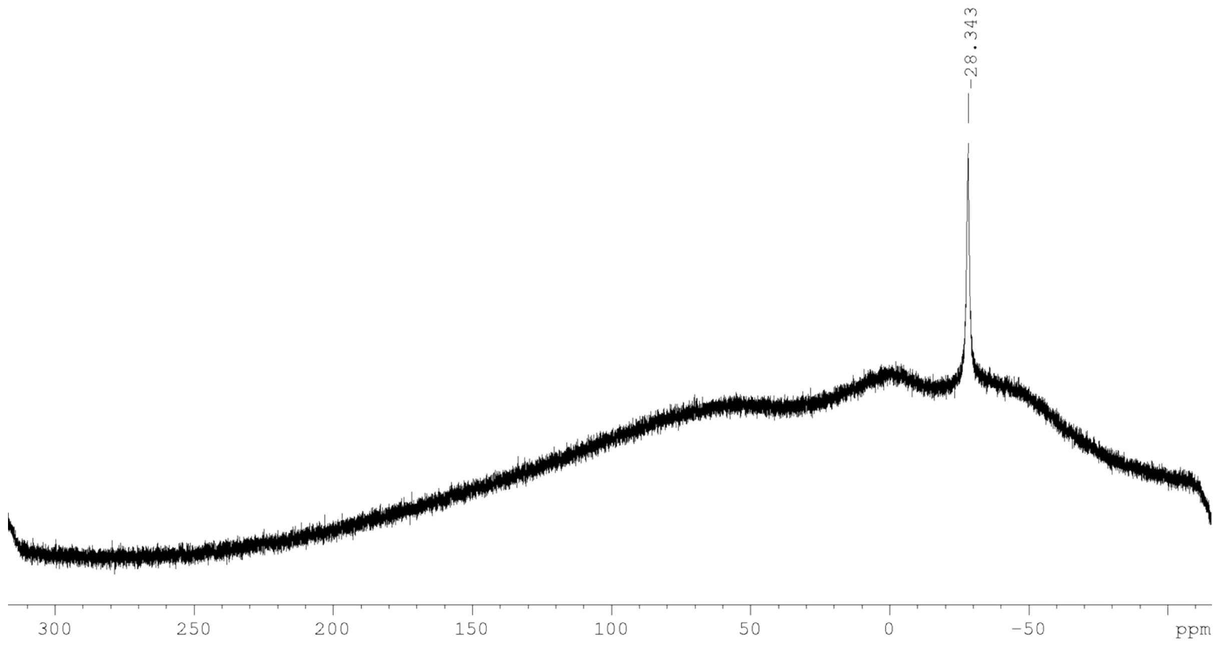

*Figure 22: ^{11}B{^{1}H} NMR spectrum of K[IMes-B(CN)$_2$], **K-8B** (96.3 MHz, THF-d8, 25 °C).*

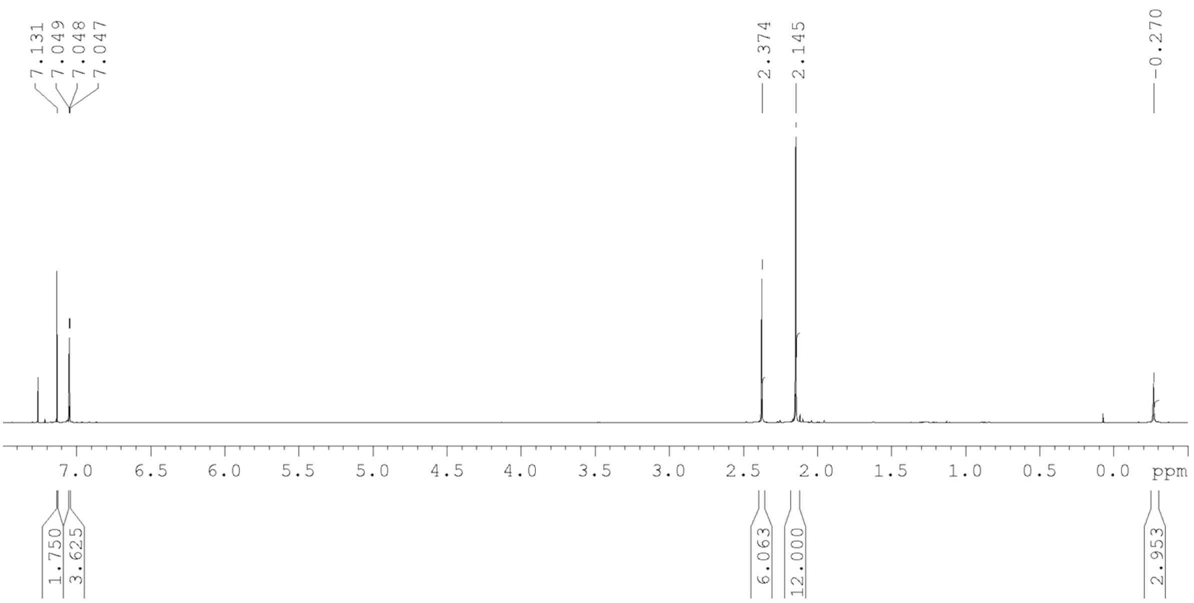

*Figure 23: ^{1}H NMR spectrum of IMes-B(CN)$_2$Me, **9** (600 MHz, CDCl$_3$, 25 °C).*

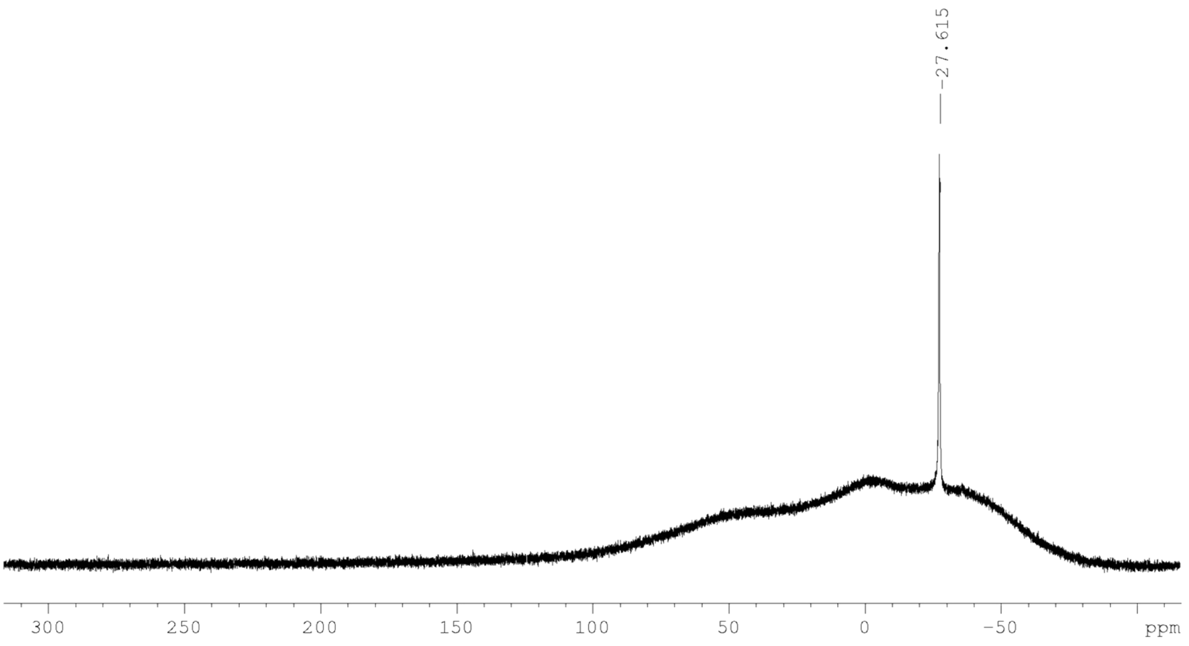

*Figure 24: ^{11}B{^{1}H} NMR spectrum of IMes-B(CN)$_2$Me, **9** (96.3 MHz, CDCl$_3$, 25 °C).*

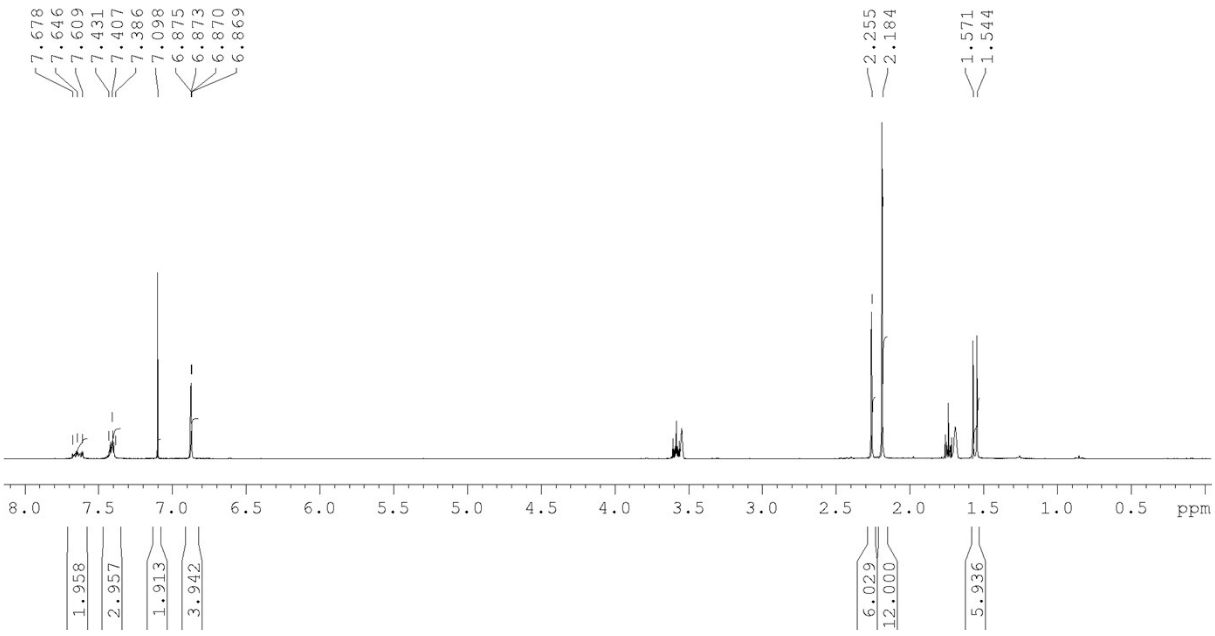

*Figure 25: ^{1}H NMR spectrum of IMes-B(CN)$_2$AuPMe$_2$Ph, **10** (300 MHz, THF-d$_8$, 25 °C).*

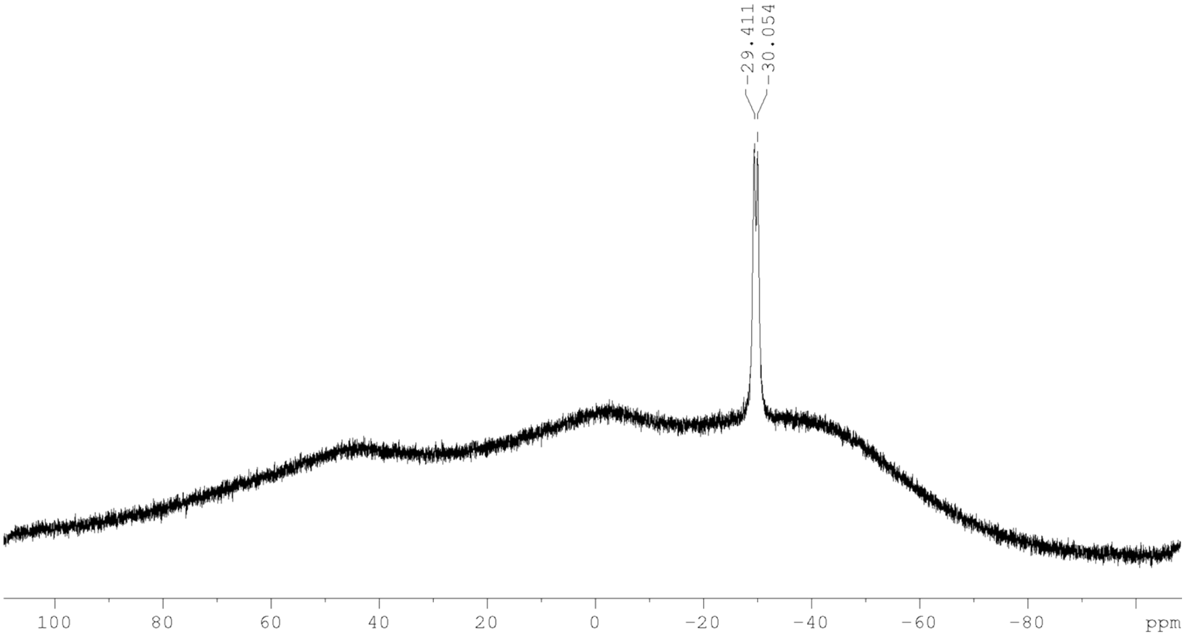

*Figure 26: $^{11}B\{^{1}H\}$ NMR spectrum of IMes-B(CN)$_2$AuPMe$_2$Ph, **10** (96.3 MHz, THF-d$_8$, 25 °C).*

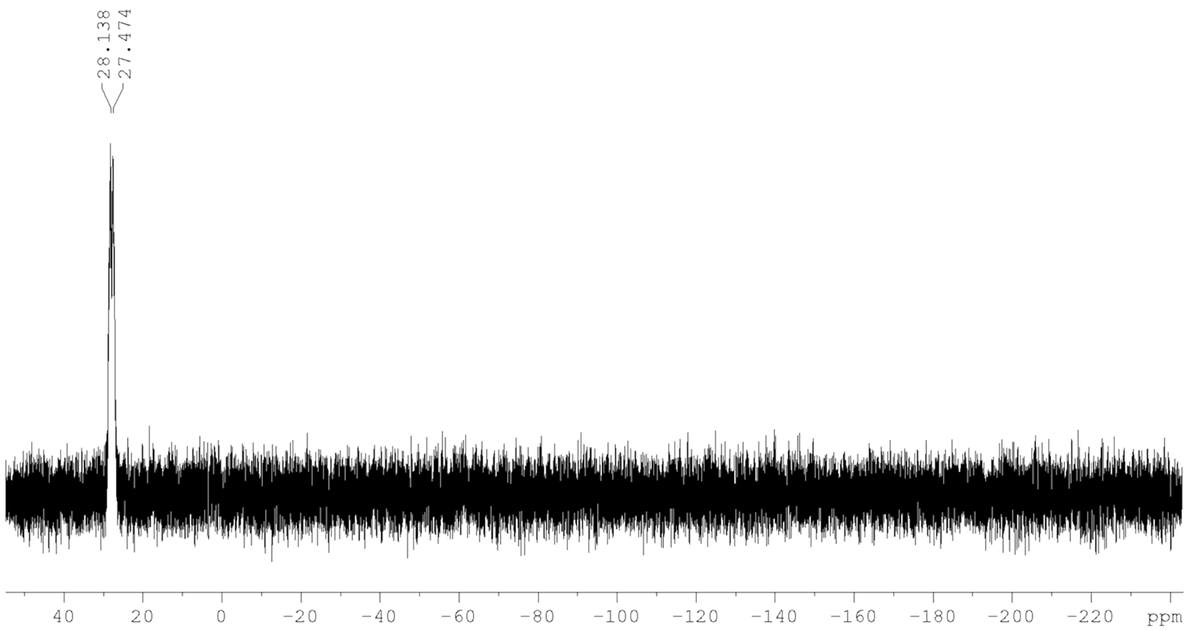

Figure 27: $^{31}P\{^{1}H\}$ *NMR spectrum of IMes-B(CN)*$_2$*AuPMe*$_2$*Ph,* ***10*** *(121.5 MHz, THF-d*$_8$*, 25 °C).*

BAC-substituted compounds

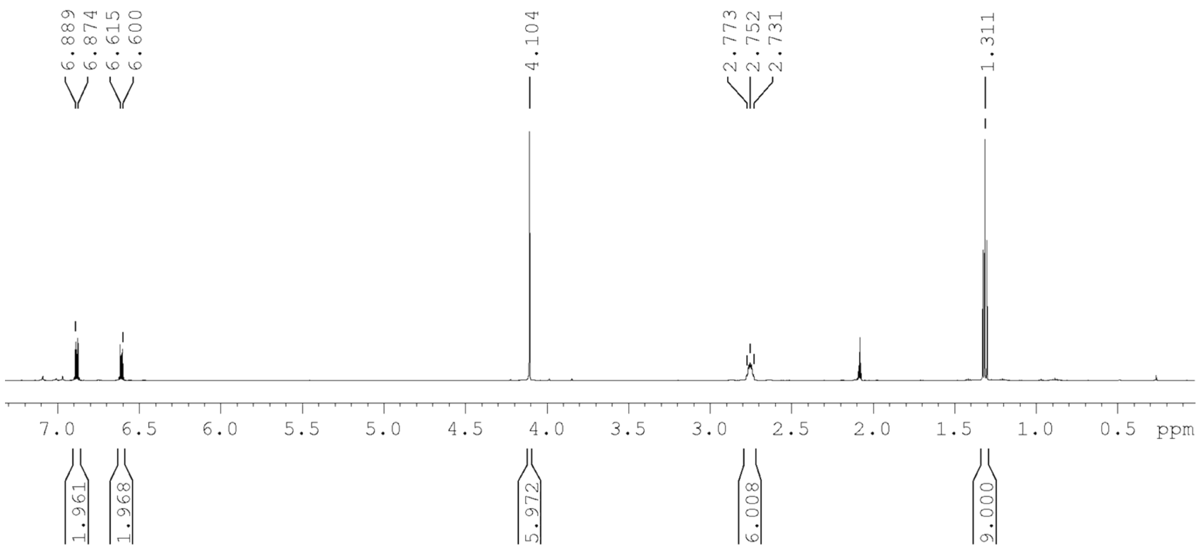

*Figure 28: ^{1}H NMR spectrum of BAC-B(SEt)$_3$, **1C** (600 MHz, toluene-d8, 25 °C).*

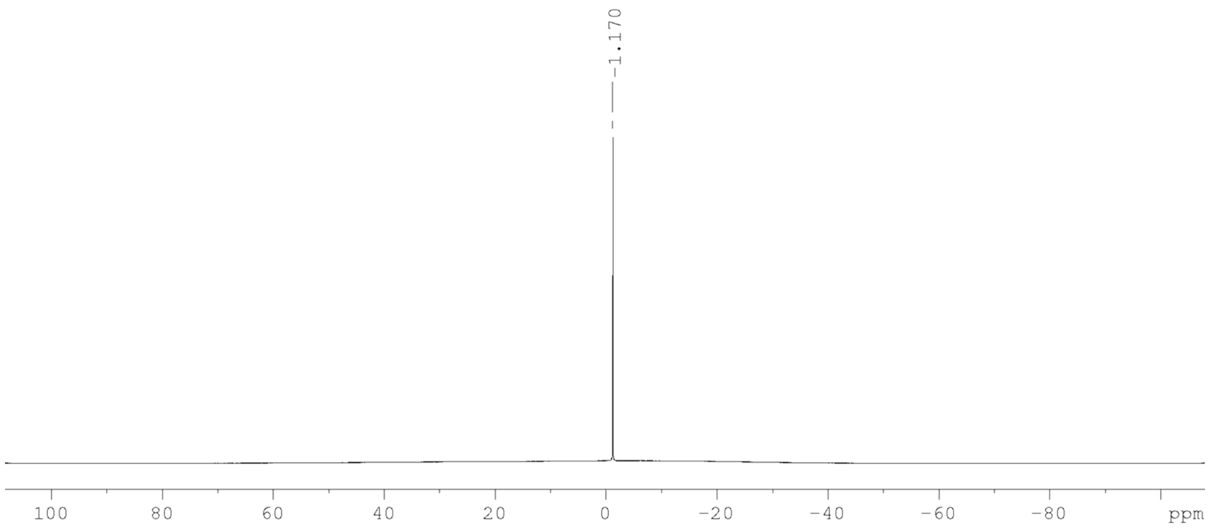

*Figure 29: $^{11}B\{^{1}H\}$ NMR spectrum of BAC-B(SEt)$_3$, **1C** (128.5 MHz, toluene-d8, 25 °C).*

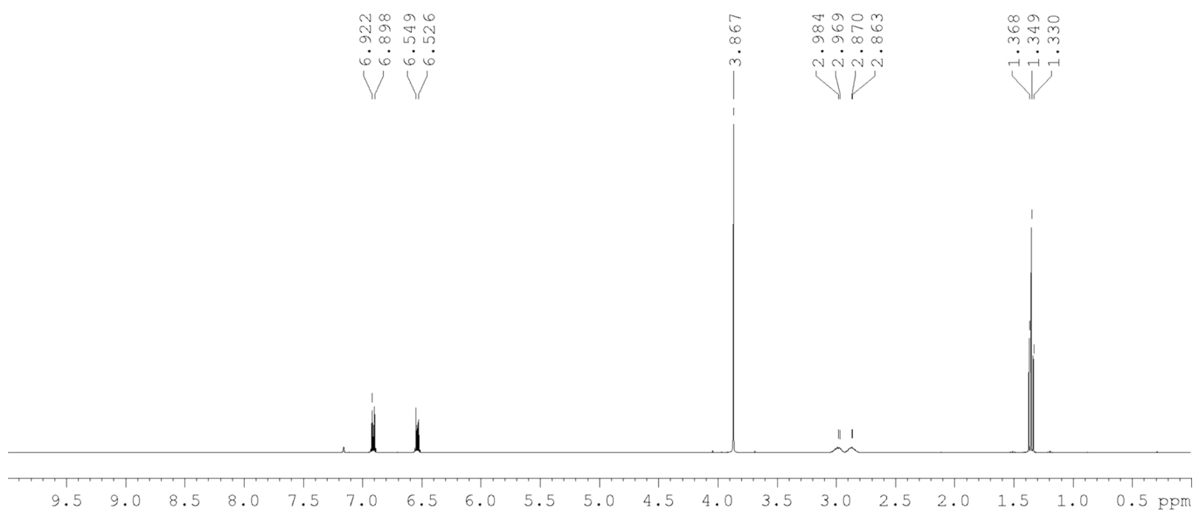

*Figure 30: ^{1}H NMR spectrum of BAC-B(SEt)$_2$CN, **2C** (400 MHz, C_6D_6, 25 °C).*

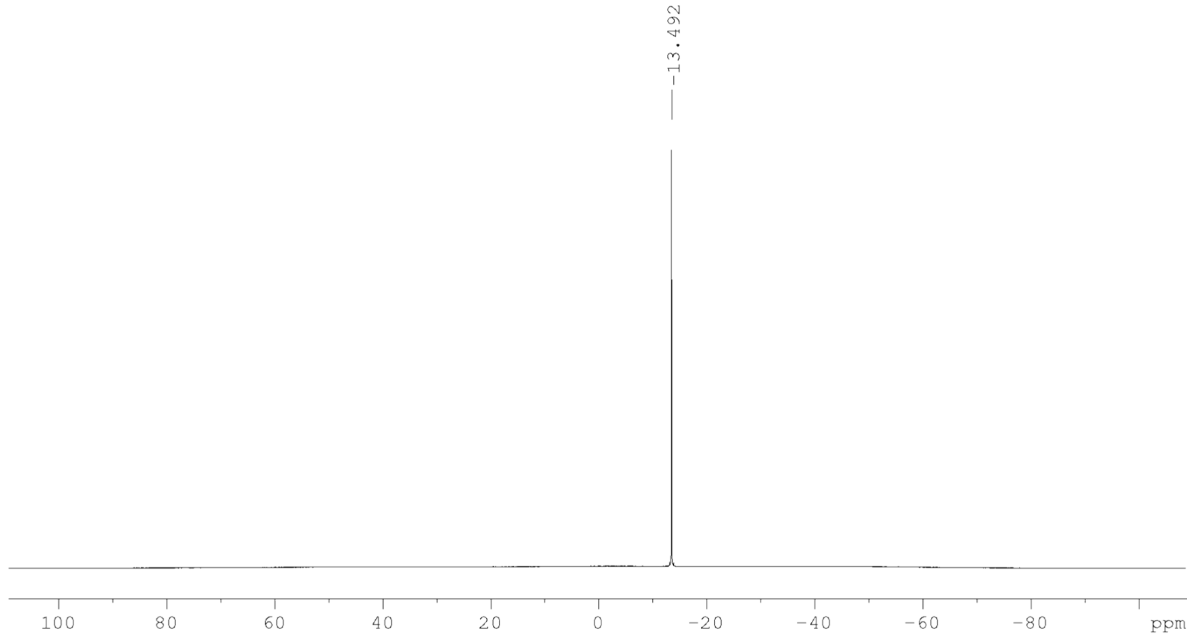

*Figure 31: ^{11}B{^{1}H} NMR spectrum of BAC-B(SEt)$_2$CN, **2C** (96.3 MHz, C_6D_6, 25 °C).*

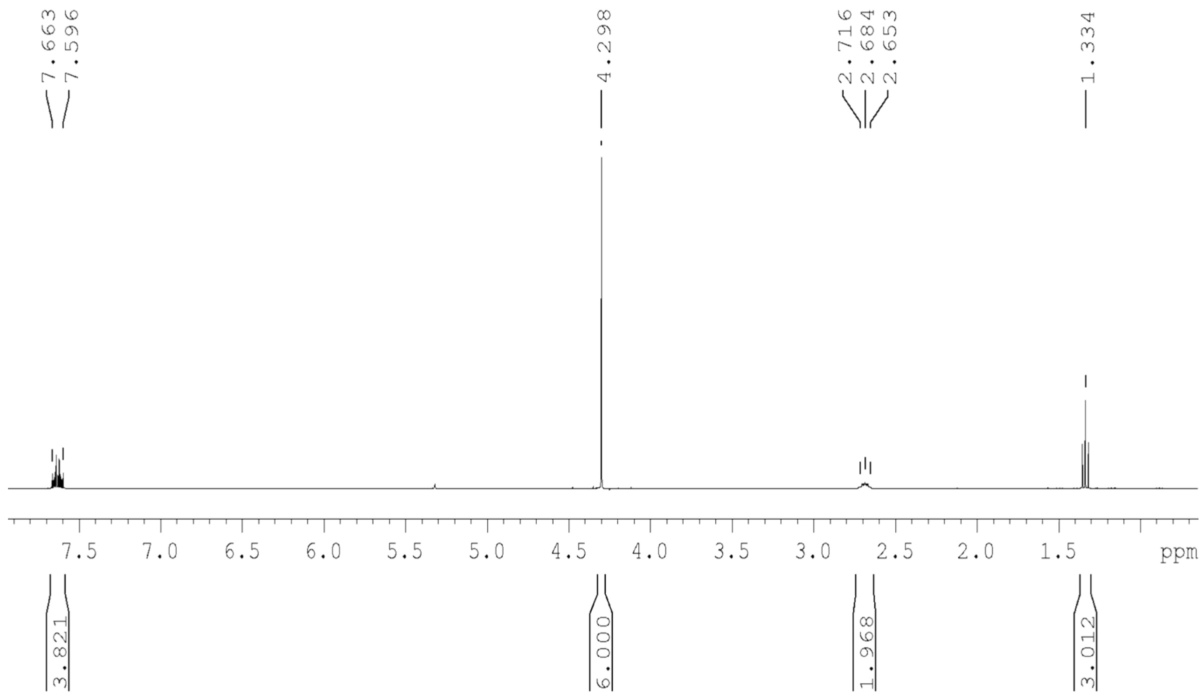

*Figure 32: ^{1}H NMR spectrum of BAC-B(CN)$_2$SEt, **3C** (400 MHz, CD_2Cl_2, 25 °C).*

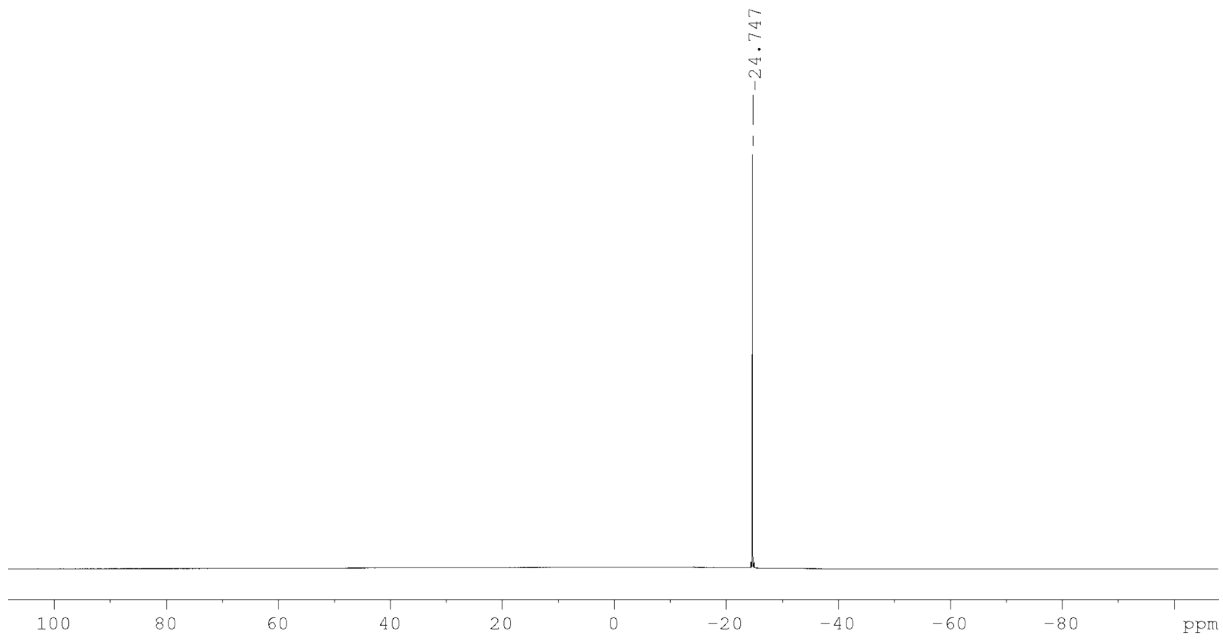

*Figure 33: $^{11}B\{^{1}H\}$ NMR spectrum of BAC-B(CN)$_2$SEt, **3C** (128.5 MHz, CD_2Cl_2, 25 °C).*

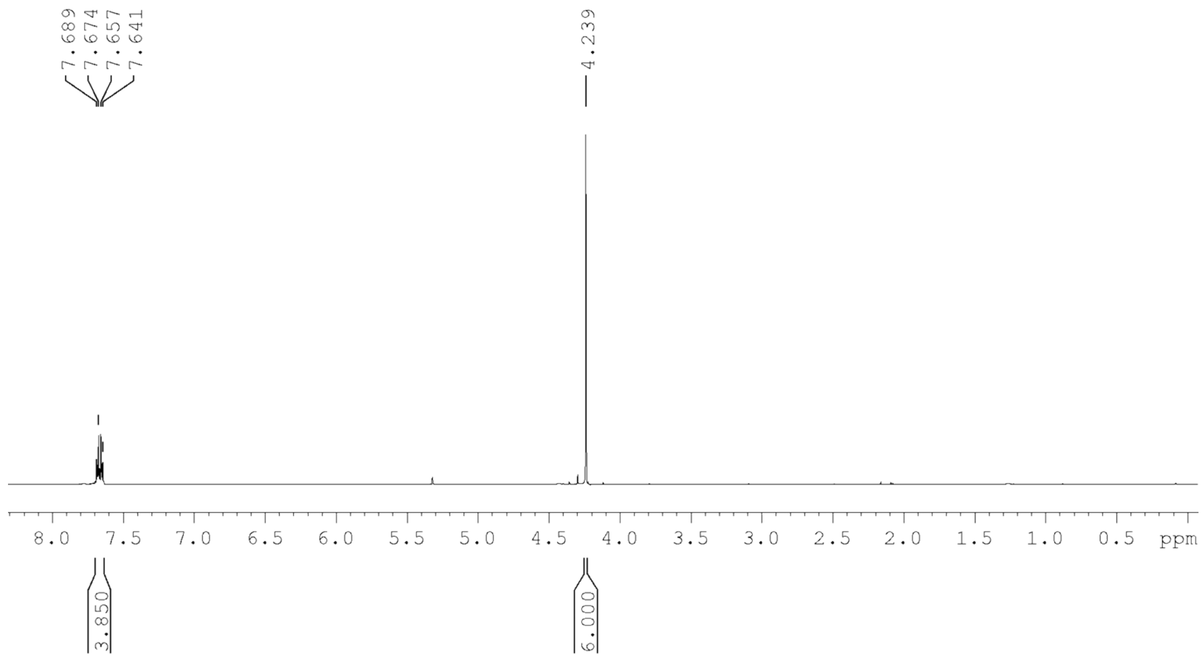

*Figure 34: ^{1}H NMR spectrum of BAC-B(CN)$_2$I, **7C** (600 MHz, CD_2Cl_2, 25 °C).*

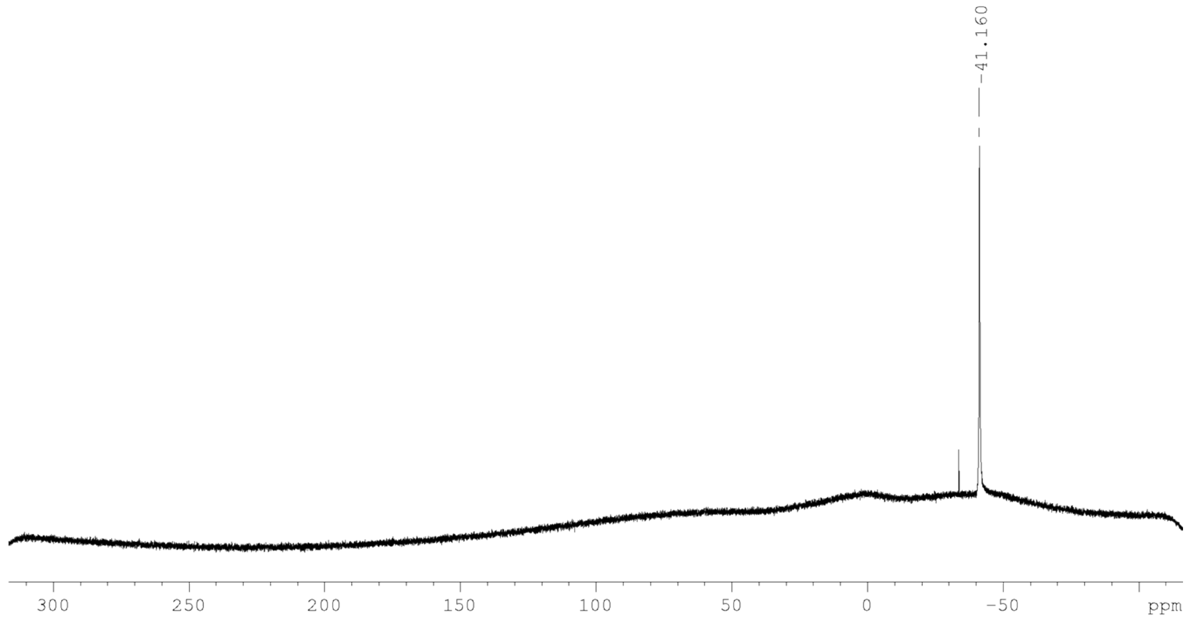

*Figure 35: $^{11}B\{^{1}H\}$ NMR spectrum of BAC-B(CN)$_2$I, **7C** (96.3 MHz, CD_2Cl_2, 25 °C).*

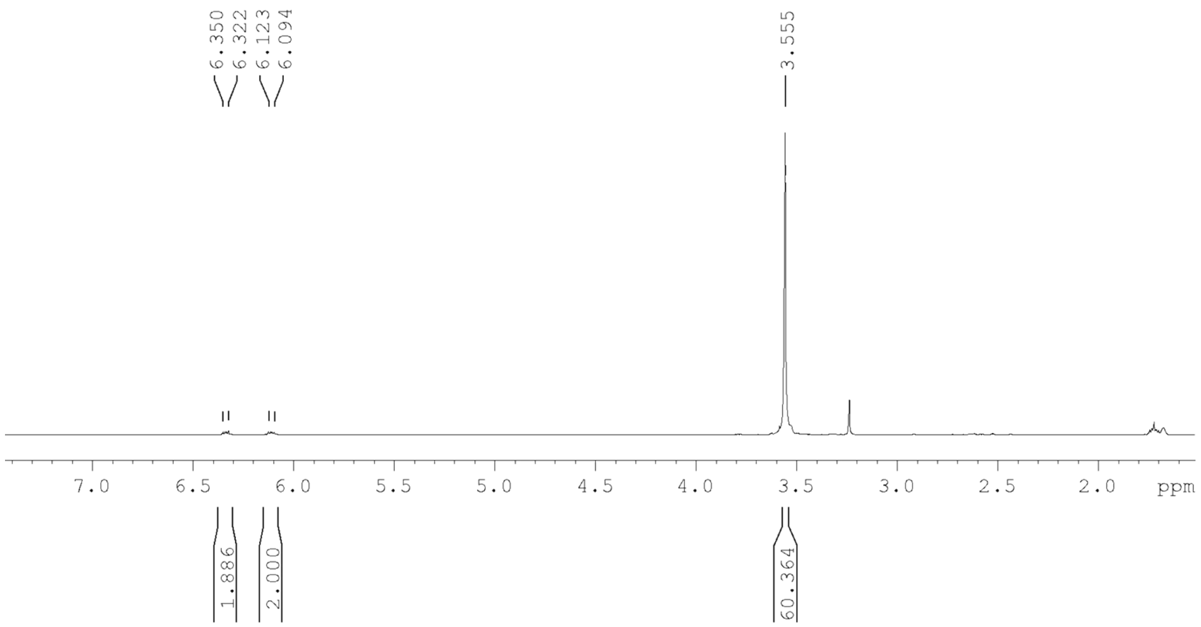

Figure 36: ^{1}H NMR spectrum of [K(18-cr-6)][BAC-B(CN)$_2$], ***[K(18-cr-6)]-8C*** *(300 MHz, THF-d8, 25 °C).*

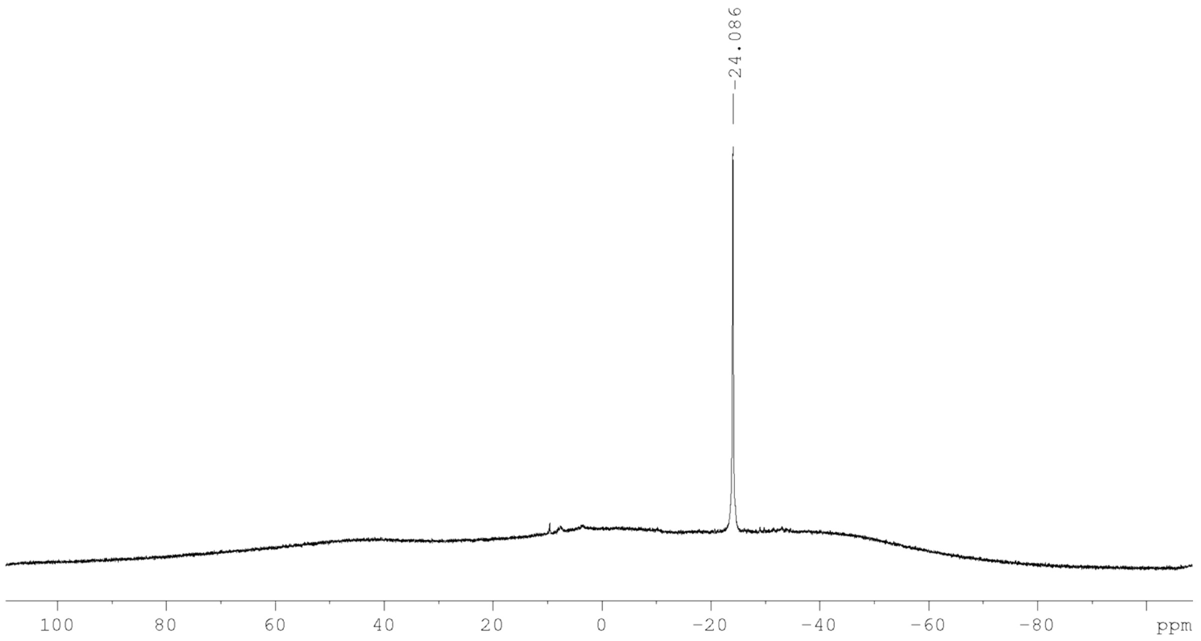

Figure 37: $^{11}B\{^{1}H\}$ NMR spectrum of [K(18-cr-6)][BAC-B(CN)$_2$], ***[K(18-cr-6)]-8C*** *(96.3 MHz, THF-d8, 25 °C).*

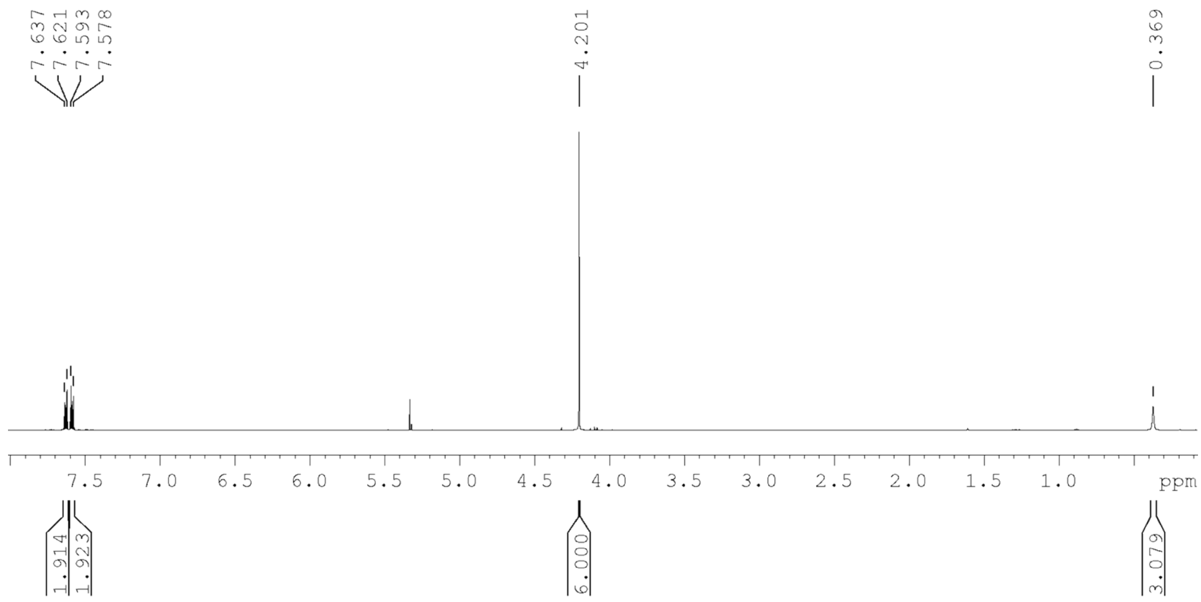

*Figure 38: ^{1}H NMR spectrum of BAC-B(CN)$_2$Me, **11** (600 MHz, CD_2Cl_2, 25 °C).*

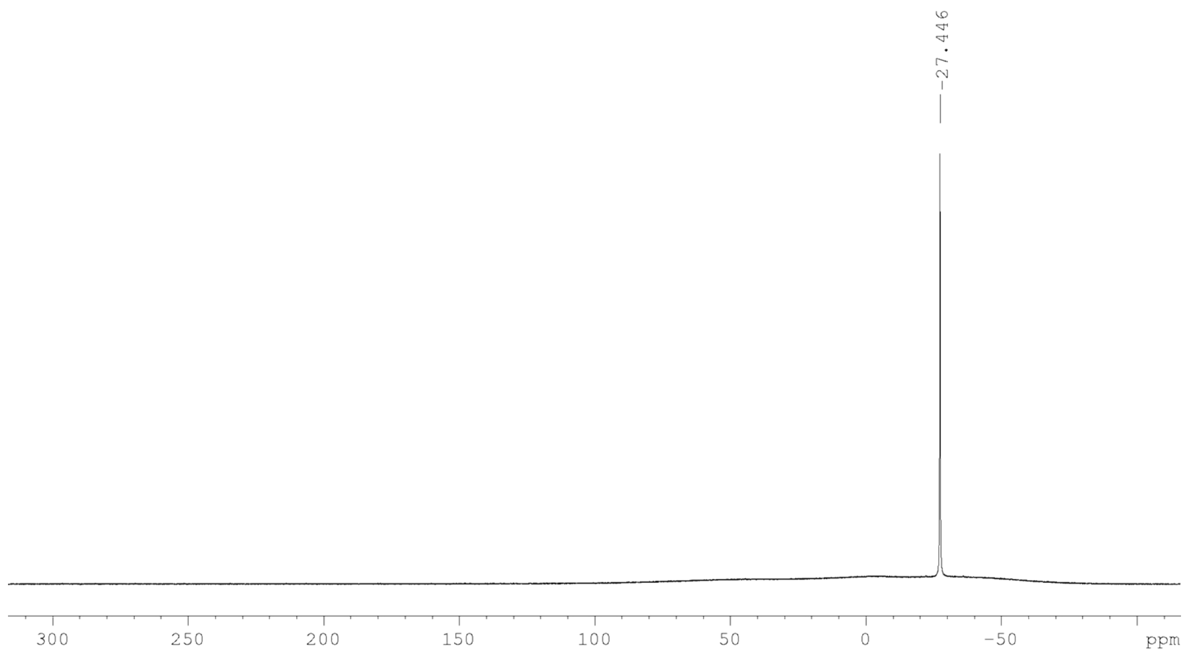

*Figure 39: $^{11}B\{^{1}H\}$ NMR spectrum of BAC-B(CN)$_2$Me, **11** (96.3 MHz, CD_2Cl_2, 25 °C).*

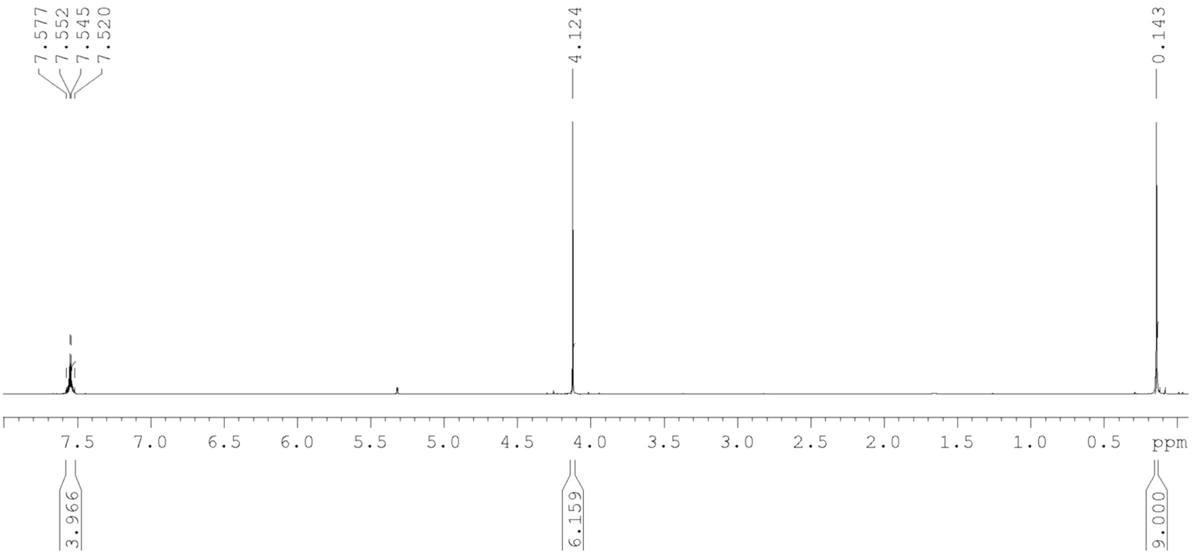

*Figure 40: ^{1}H NMR spectrum of BAC-B(CN)$_2$SiMe$_3$, **12** (400 MHz, CD_2Cl_2, 25 °C).*

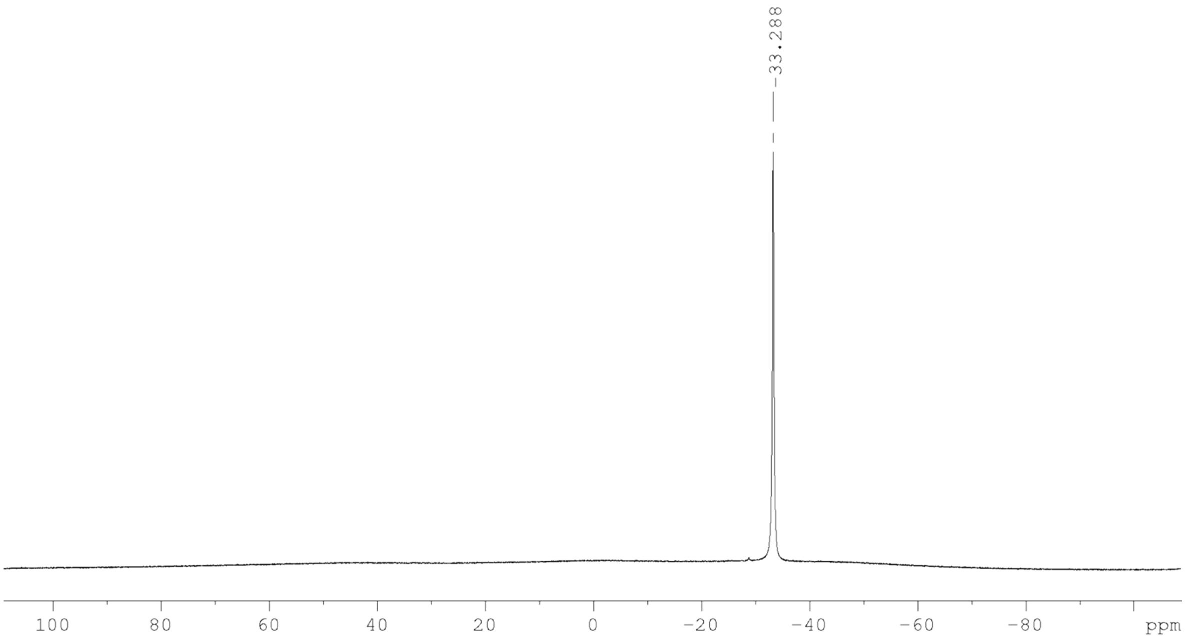

*Figure 41: $^{11}B\{^{1}H\}$ NMR spectrum of BAC-B(CN)$_2$SiMe$_3$, **12** (96.3 MHz, CD_2Cl_2, 25 °C).*

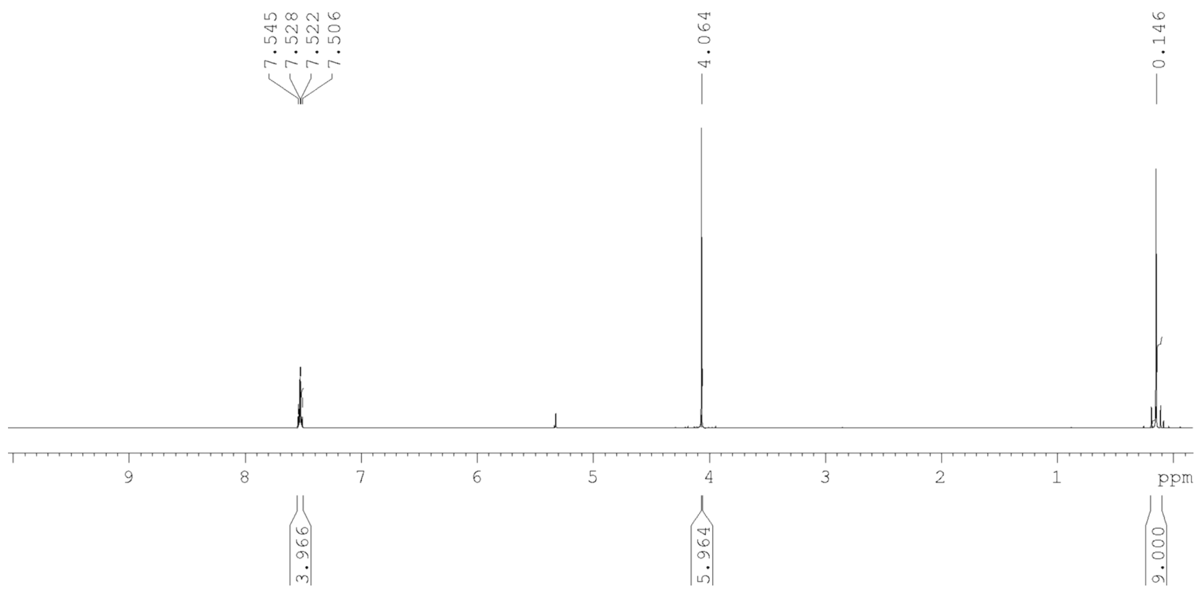

*Figure 42: ^{1}H NMR spectrum of BAC-B(CN)$_2$SnMe$_3$, **13** (600 MHz, CD_2Cl_2, 25 °C).*

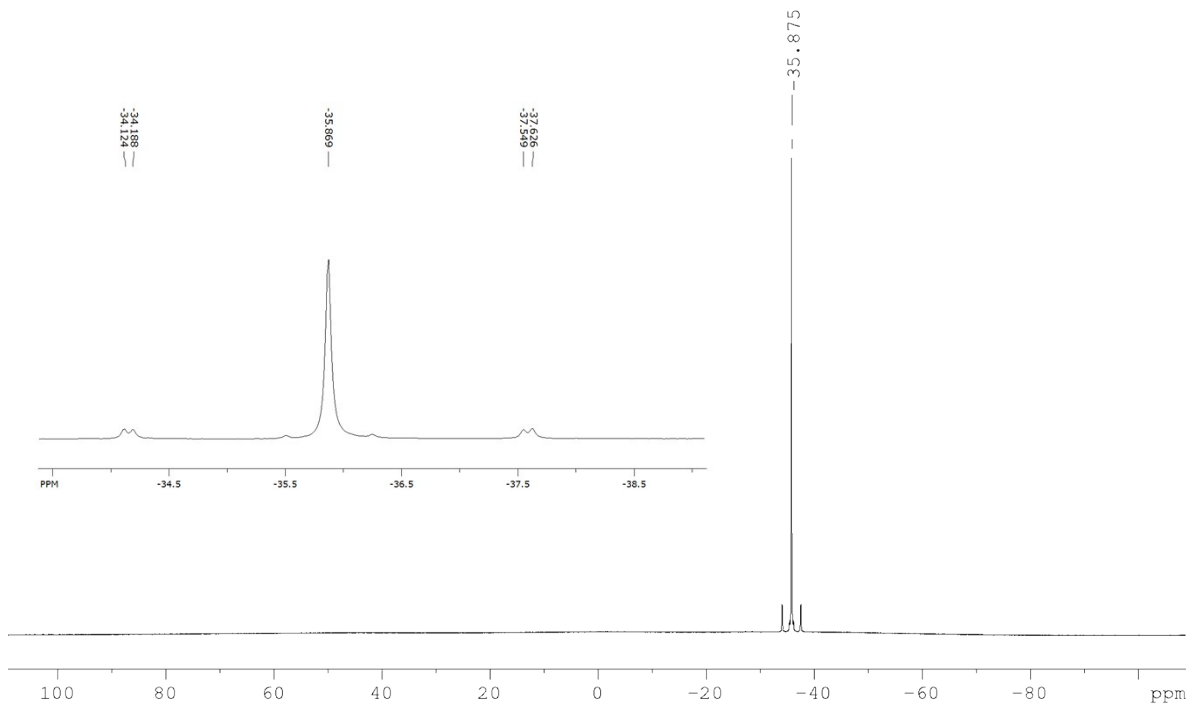

*Figure 43: $^{11}B\{^{1}H\}$ NMR spectrum of BAC-B(CN)$_2$SnMe$_3$, **13** (96.3 MHz, CD_2Cl_2, 25 °C), zoomed signal.*

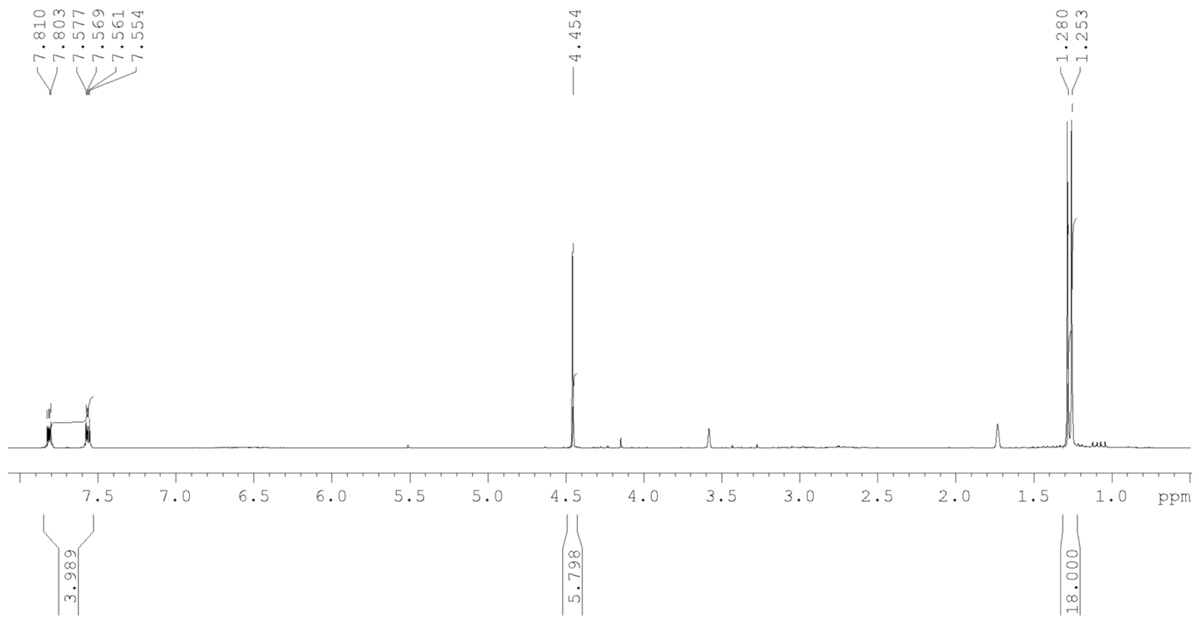

*Figure 44: ^{1}H NMR spectrum of BAC-B(CN)$_2$P(tBu)$_2$, **14** (400 MHz, THF-d_8, 25 °C).*

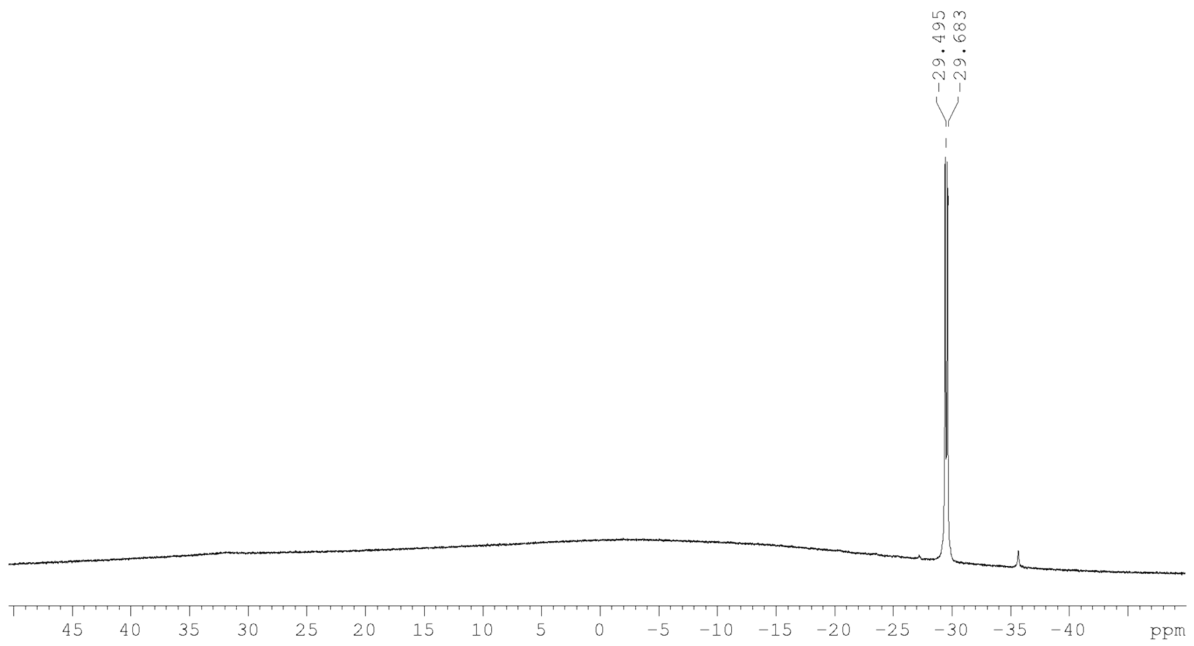

*Figure 45: $^{11}B\{^{1}H\}$ NMR spectrum of BAC-B(CN)$_2$P(tBu)$_2$, **14** (128.5 MHz, THF-d_8, 25 °C).*

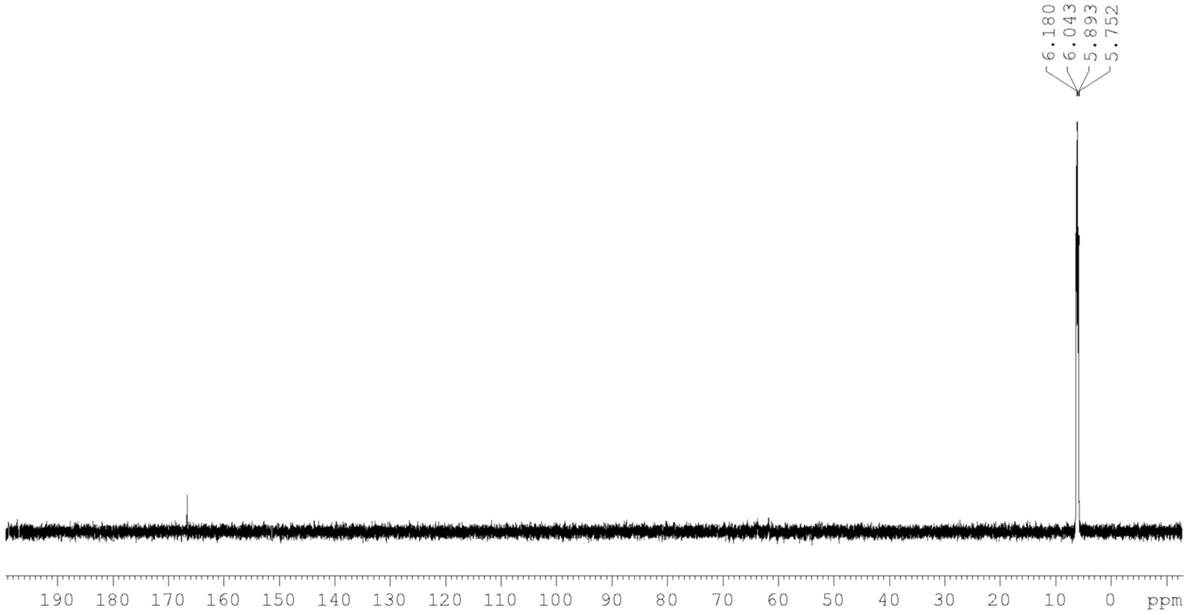

Figure 46: $^{31}P\{^{1}H\}$ NMR spectrum of BAC-B(CN)$_2$P(tBu)$_2$, ***14*** *(162.1 MHz, THF-d$_8$, 25 °C).*

IMe(Me)$_2$-substituted compounds

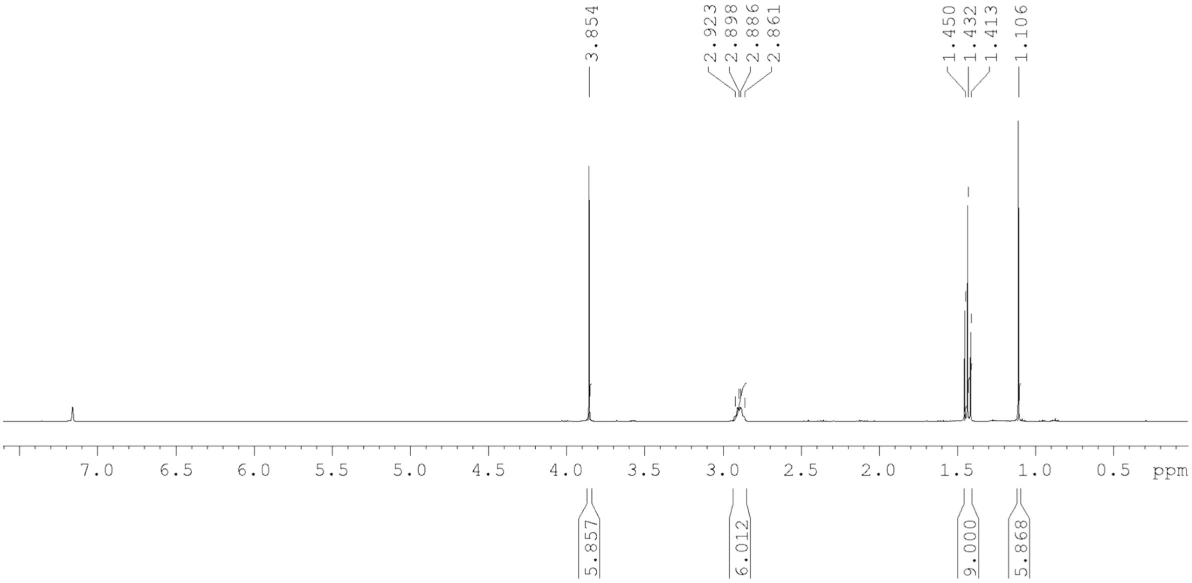

*Figure 47: ^{1}H NMR spectrum of IMe(Me)$_2$-B(SEt)$_3$, **1A** (400 MHz, C_6D_6, 25 °C).*

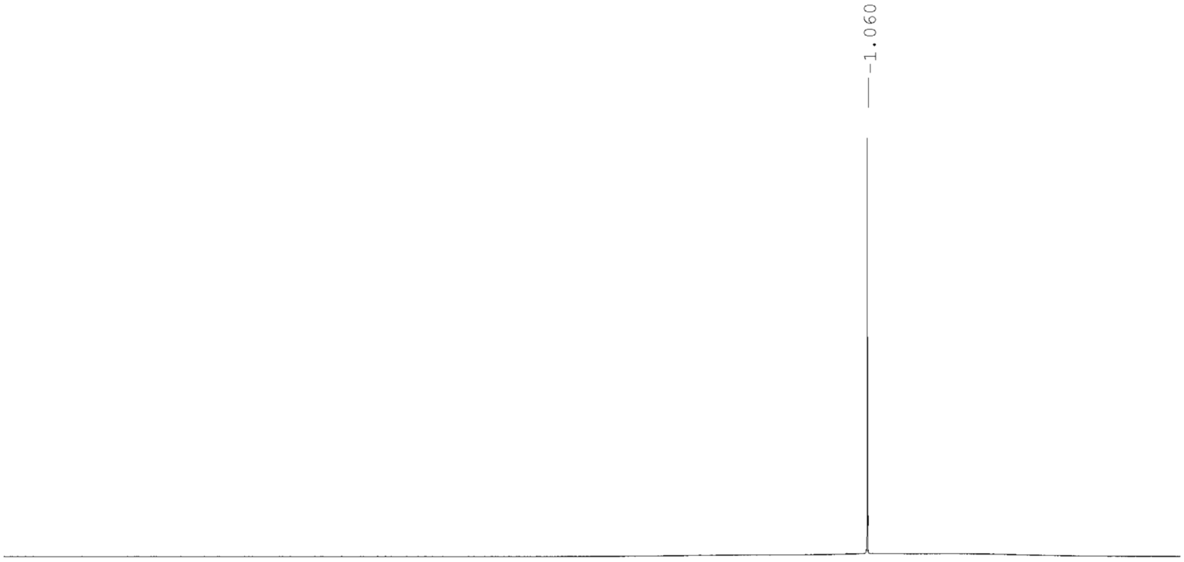

*Figure 48: ^{11}B{^{1}H} NMR spectrum of IMe(Me)$_2$-B(SEt)$_3$, **1A** (96 MHz, C_6D_6, 25 °C).*

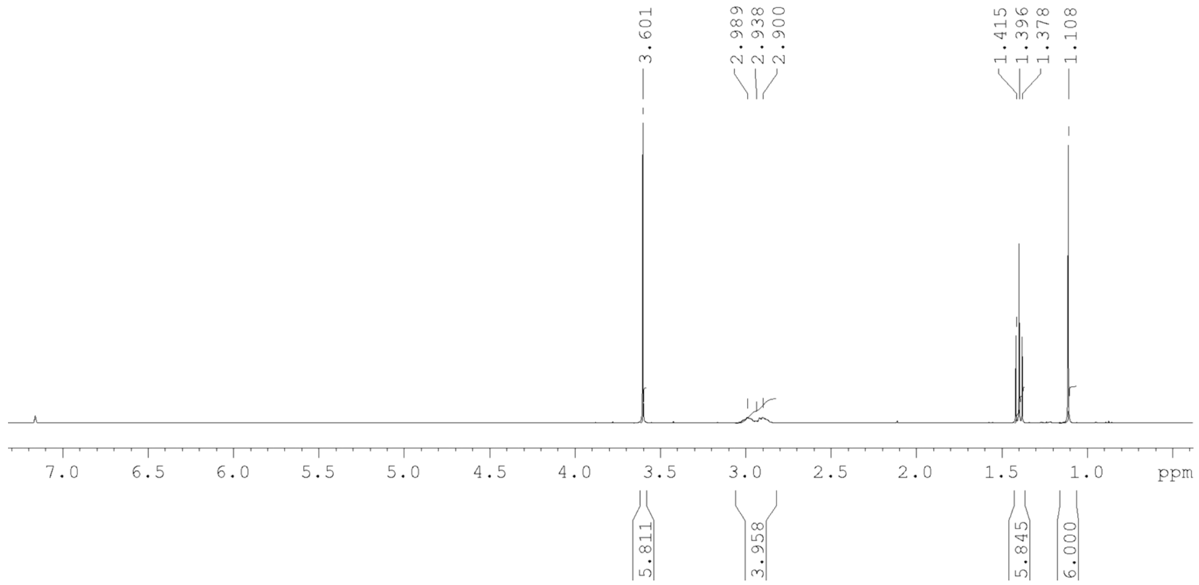

*Figure 49: ^{1}H NMR spectrum of IMe(Me)$_2$-B(SEt)$_2$CN, **2A** (400 MHz, C_6D_6, 25 °C).*

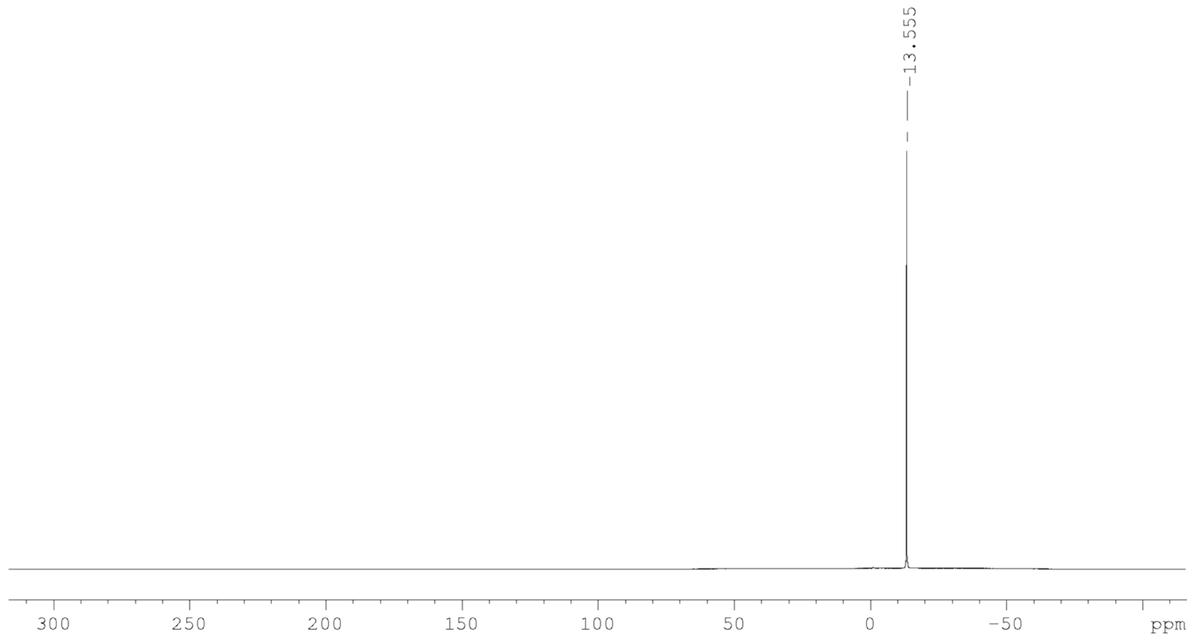

*Figure 50: $^{11}B\{^{1}H\}$ NMR spectrum of IMe(Me)$_2$-B((SEt)$_2$CN, **2A** (96.3 MHz, C_6D_6, 25 °C).*

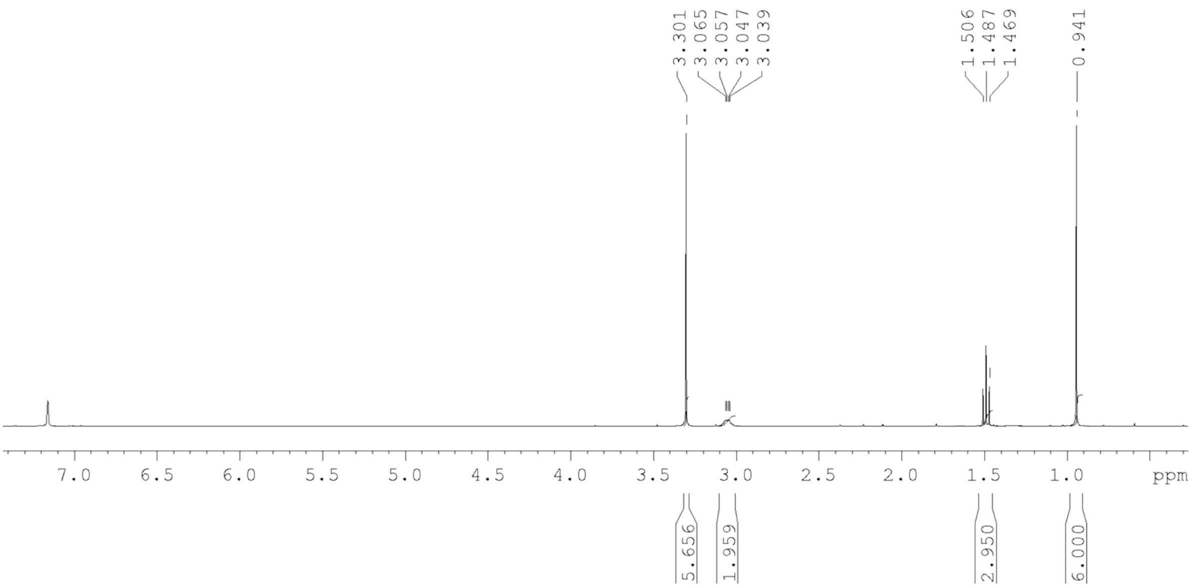

*Figure 51: ^{1}H NMR spectrum of IMe(Me)$_2$-B(CN)$_2$(SEt), **3A** (400.4 MHz, C_6D_6, 25 °C).*

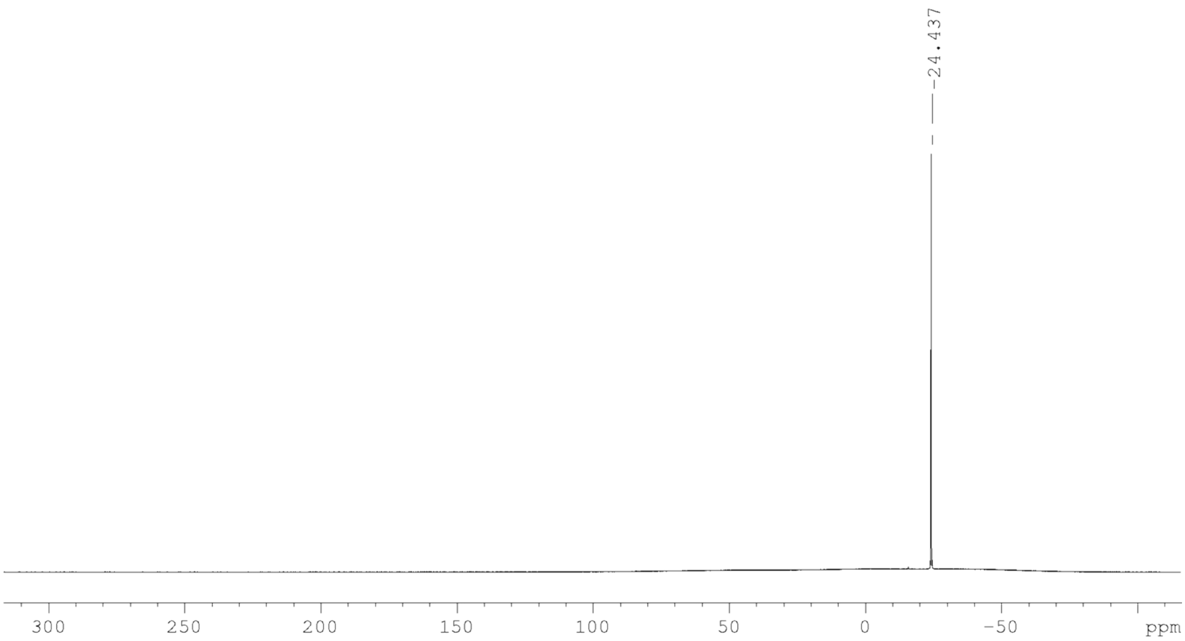

*Figure 52: $^{11}B\{^{1}H\}$ NMR spectrum of IMe(Me)$_2$-B(CN)$_2$(SEt), **3A** (96.3 MHz, C_6D_6, 25 °C).*

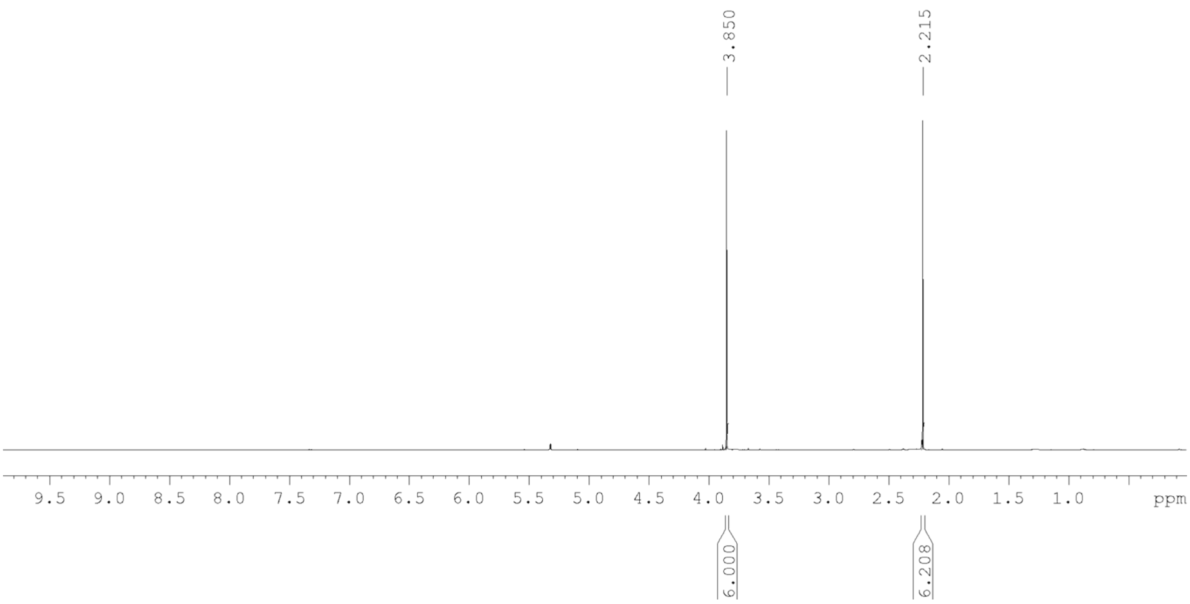

*Figure 53: ^{1}H NMR spectrum of IMe(Me)$_2$-B(CN)$_2$I, **7A** (400.4 MHz, CD_2Cl_2, 25 °C).*

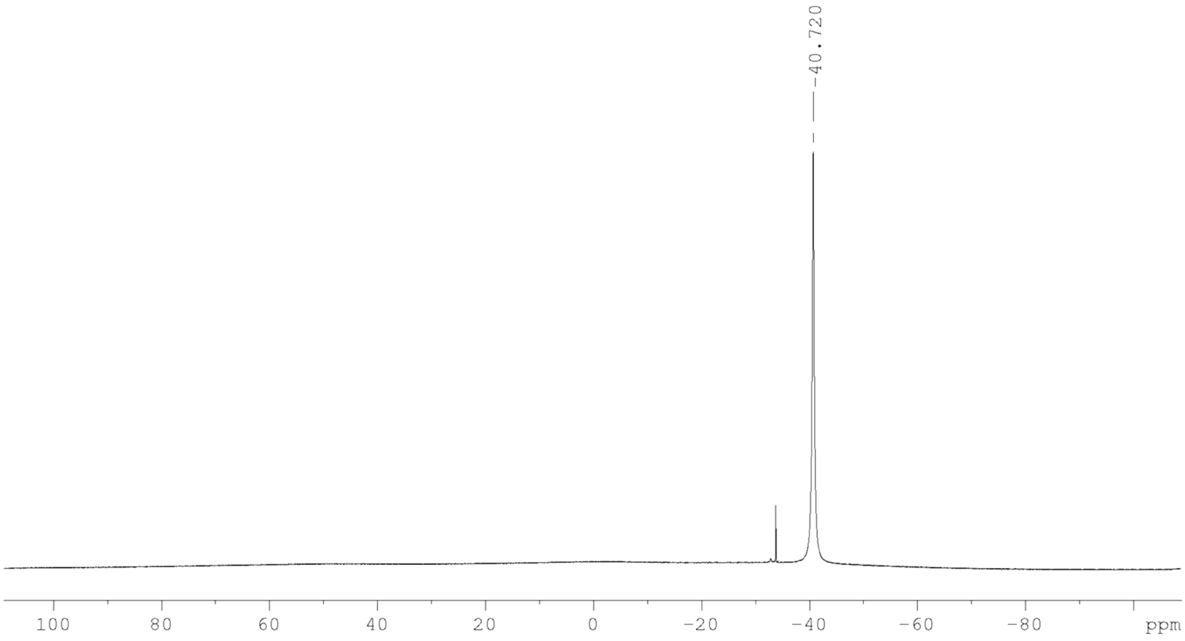

*Figure 54: $^{11}B\{^{1}H\}$ NMR spectrum of IMe(Me)$_2$-B(CN)$_2$I, **7A** (96.3 MHz, CD_2Cl_2, 25 °C).*

X-ray Structure Analysis

Table 1: Selected Crystallographic Data for IMes-based Compounds Measured by X-ray Structure Analysis.

	**1B**	**2B**	**7B**	**K-8B·4THF**	**9**
CCDC	1550754	1550755	1550760	1550767	1550769
Formula	$C_{27}H_{39}BN_2S_3$	$C_{26}H_{34}BN_3S_2$	$C_{23}H_{24}BIN_4$	$C_{243}H_{296}B_9K_9N_{36}O_4$	$C_{24}H_{27}BN_4$
M [g mol^{-1}]	498.59	463.49	494.17	4234.35	382.31
T/K	130(2)	100(2)	100(2)	100(2)	100(2)
λ/Å	1.54184	1.54184	0.71073	1.54184	1.54184
Crystal system	Orthorhombic	Monoclinic	Monoclinic	Monoclinic	Orthorhombic
Space group	$P2_12_12_1$	$P2_1/c$	$P2_1/n$	$P2_1/c$	*Pbca*
a/Å	12.63216(10)	8.20315(10)	16.5750(3)	33.0927(5)	16.1393(3)
b/Å	13.55052(10)	16.7145(2)	17.3066(2)	30.3266(4)	7.9763(2)
c/Å	16.37821(10)	37.3775(7)	23.9859(4)	25.1212(4)	34.8942(7)
α/°	90	90	90	90	90
β/°	90	95.505(2)	90.036(2)	106.881(2)	90
γ/°	90	90	90	90	90
V/Å^3	2803.50(3)	5101.26(13)	6880.51(19)	24125.0(6)	4492.02(17)
Z	4	8	12	4	8
D_x/g cm^{-3}	1.181	1.207	1.431	1.166	1.131
μ/mm^{-1}	2.533	2.017	1.412	1.898	0.518
F(000)	1072	1984	2976	9016	1632
Cryst. size/mm	0.22x0.10x0.08	0.15x0.10x0.03	0.22x0.20x0.18	0.22x0.15x0.10	0.30x0.18x0.16
Theta range/°	4.23 to 76.31	3.55 to 76.30	2.35 to 30.03	3.21 to 76.36	3.73 to 76.32
Reflections collected	74082	92739	398935	563295	86768
Indep. reflections	5849	10650	20085	50397	4686
R(int)	0.0620	0.0699	0.0571	0.0662	0.0410
Data / restraints / parameters	5849 / 0 / 307	10650 / 0 / 593	20085 / 0 / 803	50397 / 0 / 2404	4686 / 0 / 269
GooF	1.050	1.027	1.059	1.020	1.057
R1 (F, >4σ(F))	0.0256	0.0359	0.0292	0.0390	0.0514
wR2 (F^2, all refl.)	0.0693	0.0937	0.0627	0.1013	0.1501
Flack parameter	0.000(9)	—	—	—	—
max. Δρ/e Å^{-3}	0.326/−0.252	0.295/−0.291	0.538/−0.486	0.207/−0.267	0.302/−0.237

Table 2: Selected Crystallographic Data for BAC-based Compounds Measured by X-ray Structure Analysis.

	**1C**	**2C**	**7C**	**[K(18-cr-6)]-8C**	**11·½CH₂Cl₂**
CCDC	1550740	1550756	1550774	1550768	1550770
Formula	$C_{15}H_{25}BN_2S_3$	$C_{14}H_{20}BN_3S_2$	$C_{11}H_{10}BIN_4$	$C_{46}H_{68}B_2K_2N_8O_{12}$	$C_{12.50}H_{14}BClN_4$
M [g mol^{-1}]	340.36	305.26	335.94	1024.90	266.54
T/K	100(2)	100(2)	100(2)	100(2)	100(2)
λ/Å	0.71073	0.71073	0.71073	1.54184	1.54184
Crystal system	Triclinic	Monoclinic	Orthorhombic	Monoclinic	Orthorhombic
Space group	$P\bar{1}$	$P2_1/n$	*Pnnm*	$P2_1/c$	$Cmc2_1$
a/Å	8.4435(2)	10.4737(2)	8.8243(2)	9.0098(1)	11.3418(3)
b/Å	8.5770(2)	12.0926(2)	10.9964(2)	22.6206(2)	18.4372(5)
c/Å	12.2867(3)	13.1679(3)	12.7995(3)	13.0457(1)	12.9532(3)
α/°	84.407(2)	90	90	90	90
β/°	89.589(2)	104.764(2)	90	100.351(1)	90
γ/°	82.070(2)	90	90	90	90
V/Å^3	877.08(4)	1612.71(5)	1242.00(5)	2615.54(4)	2708.64(12)
Z	2	4	4	2	8
D_x/g cm^{-3}	1.289	1.257	1.797	1.301	1.307
μ/mm^{-1}	0.417	0.323	2.560	2.148	2.394
F(000)	364	648	648	1088	1112
Cryst. size/mm	0.40x0.30x0.15	0.40x0.30x0.15	0.28x0.22x0.18	0.18x0.18x0.02	0.20x0.12x0.08
Theta range/°	2.41 to 31.09	2.23 to 31.38	2.44 to 31.08	3.908 to 76.170	4.58 to 76.21
Reflections collected	89317	120276	31556	53304	27441
Indep. reflections	5288	5120	2021	5473	2795
R(int)	0.0363	0.0458	0.0353	0.0664	0.0400
Data / restraints / parameters	5288 / 0 / 195	5120 / 0 / 185	2021 / 0 / 83	5473 / 0 / 319	2795 / 1 / 184
GooF	1.044	1.064	1.107	1.047	1.049
R1 (F, >4σ(F))	0.0296	0.0364	0.0185	0.0361	0.0327
wR2 (F^2, all refl.)	0.0750	0.0868	0.0456	0.0986	0.0861
Flack parameter	—	—	—	—	0.019(17)
max. Δρ/e Å^{-3}	0.420/−0.287	0.433/−0.313	0.489/−0.411	0.376/−0.284	0.238/−0.401

Table 3: Selected Crystallographic Data for BAC-, IMe(Me)$_2$- and TMS-based Compounds Measured by X-ray Structure Analysis.

	**13**	**14·CH_2Cl_2**	**3A**	**7A·CH_2Cl_2**	**4**
CCDC	1550771	1550772	1550757	1550759	1550758
Formula	$C_{14}H_{19}BN_4Sn$	$C_{20}H_{30}BCl_2N_4P$	$C_{11}H_{17}BN_4S$	$C_{10}H_{14}BCl_2IN_4$	$C_8H_{14}BN_3SSi$
M [g mol^{-1}]	372.83	439.16	248.16	398.86	223.18
T/K	100(2)	100(2)	100(2)	100(2)	100(2)
λ/Å	0.71073	1.54184	0.71073	0.71073	1.54184
Crystal system	Monoclinic	Orthorhombic	Orthorhombic	Orthorhombic	Monoclinic
Space group	$P2_1/c$	*Pbcn*	$P2_12_12_1$	$Cmc2_1$	$P2_1/c$
a/Å	11.0134(1)	31.987(2)	8.5576(3)	10.8977(4)	9.5297(2)
b/Å	21.1517(2)	8.6554(6)	9.7069(3)	12.4002(4)	8.5096(2)
c/Å	13.8999(1)	16.7007(9)	16.4514(6)	11.6286(2)	16.0181(4)
α/°	90	90	90	90	90
β/°	90.555(1)	90	90	90	94.741(2)
γ/°	90	90	90	90	90
V/$Å^3$	3237.86(5)	4623.7(5)	1366.60(8)	1571.42(8)	1294.52(5)
Z	8	8	4	4	4
D_x/g cm^{-3}	1.530	1.262	1.206	1.686	1.145
μ/mm^{-1}	1.573	3.272	0.221	2.366	2.852
F(000)	1488	1856	528	776	472
Cryst. size/mm	0.40x0.10x0.10	0.15x0.08x0.06	0.30x0.25x0.18	0.25x0.20x0.12	0.16x0.08x0.03
Theta range/°	2.420 to 31.094	5.29 to 76.23	2.44 to 31.08	2.49 to 30.96	4.66 to 76.22
Reflections collected	91552	50662	36589	20643	25988
Indep. reflections	9835	4827	4129	2470	2701
R(int)	0.0416	0.0833	0.0500	0.0367	0.0501
Data / restraints / parameters	9835 / 0 / 371	4827 / 0 / 261	4129 / 0 / 159	2470 / 1 / 93	2701 / 0 / 131
GooF	1.102	1.032	1.062	1.062	1.060
R1 (F, >4σ(F))	0.0263	0.0393	0.0380	0.0169	0.0267
wR2 (F^2, all refl.)	0.0520	0.0990	0.0823	0.0359	0.0704
Flack parameter	—	—	0.01(6)	−0.037(13)	—
max. Δρ/e $Å^{-3}$	0.568/−0.640	0.381/−0.460	0.465/−0.408	0.308/−0.342	0.305/−0.239

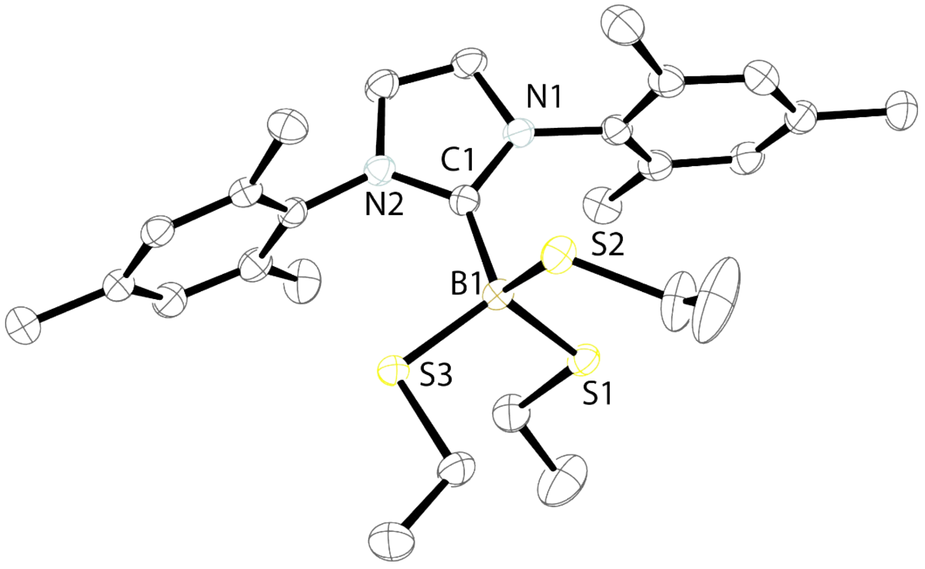

*Figure 55: ORTEP diagram of IMes-B(SEt)$_3$, **1B**. Ellipsoids are drawn at 50% probability levels. Hydrogen atoms are omitted for clarity. Selected bond lengths [Å] and angles [°]: S1-B1 1.9210(15), S2-B1 1.9363(15), S3-B1 1.9277(15), B1-C1 1.6421(19), N1-C1 1.3633(18), N2-C1 1.3589(18), C1-B1-S1 111.89(9), C1-B1-S2 109.39(7), C1-B1-S3 111.63(7).*

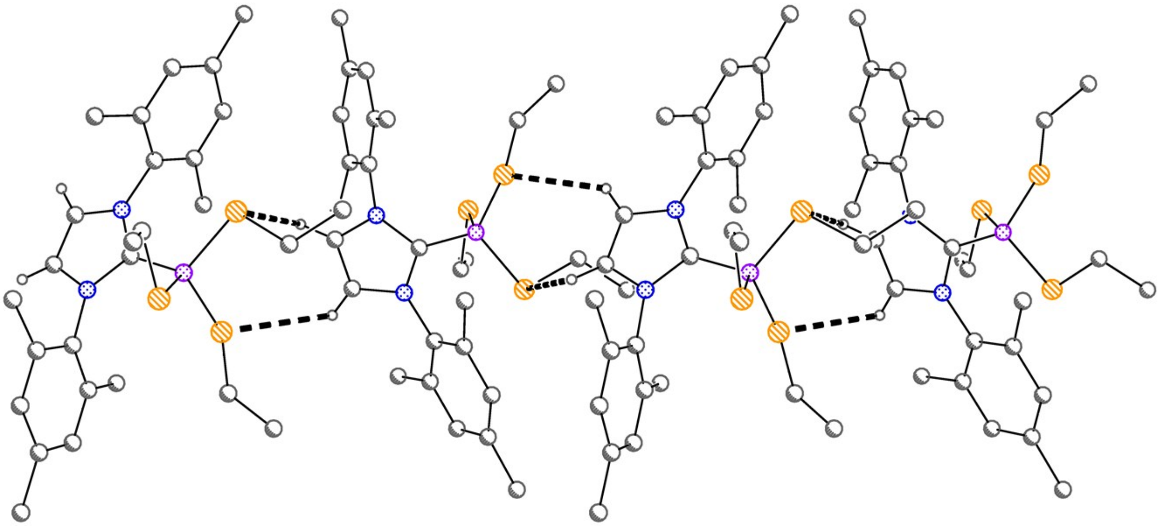

*Figure 56: Packing diagram of IMes-B(SEt)$_3$, **1B**. The molecules are linked in chains parallel to the b axis by the contacts H2□S3 2.97 and H3□S2 2.85 Å (shown as dashed bonds).*

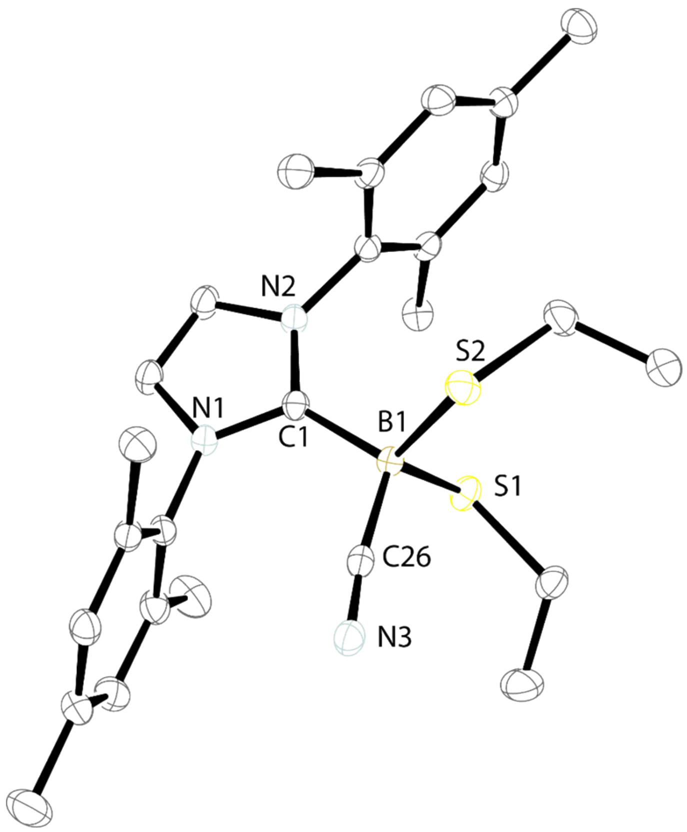

*Figure 57: ORTEP diagram of IMes-B(SEt)$_2$CN, **2B**. Ellipsoids are drawn at 50% probability levels. Hydrogen atoms are omitted for clarity. Selected bond lengths [Å] and angles [°]: S1-B1 1.9121(17), S2-B1 1.9450(16), B1-C26 1.593, B1-C1 1.632, N1-C1 1.3561(19), N2-C1 1.3586(18), C1-B1-S1 104.89(10), C1-B1-S2 114.77(8), C1-B1-C26 111.92(12). The asymmetric unit contains two independent molecules, of which only one is shown here; a least-squares fit of all non-hydrogen atoms except ethyl C gives an r.m.s. deviation of 0.2 Å.*

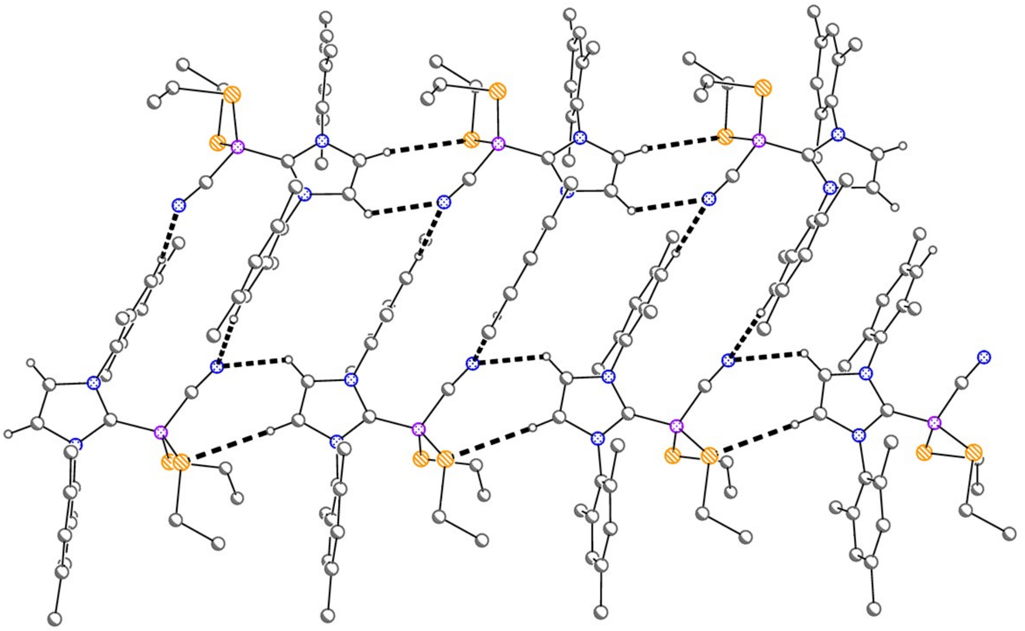

*Figure 58: Packing diagram of IMes-B(SEt)$_2$CN, **2B**. Each independent molecule is linked in chains parallel to the a axis by the contacts H2□N3 2.44, H3□S2 2.98, H2'□N3' 2.42, H3'□S2' 2.91 Å (shown as dashed bonds); the chains are crosslinked to form ribbons by the contacts H8□N3' 2.61 and H8'□N3 2.69 Å.*

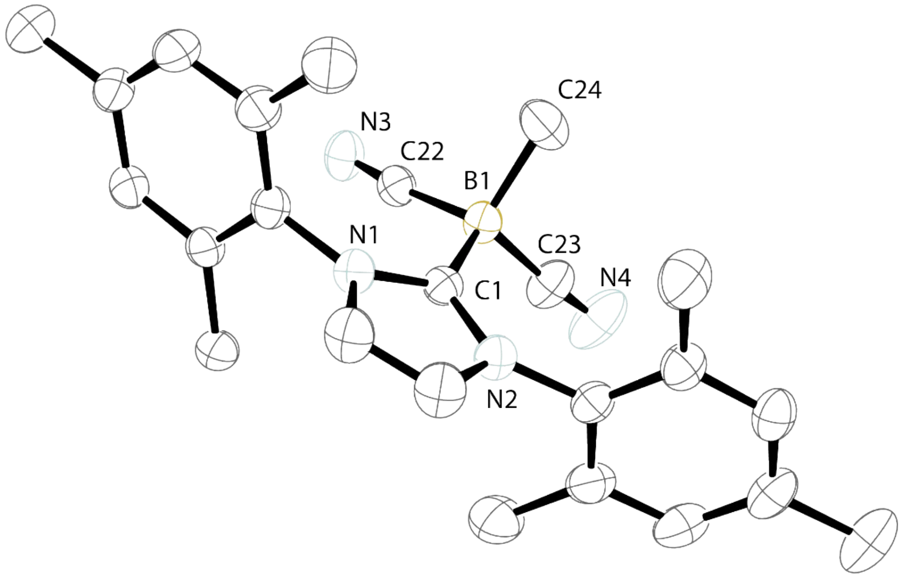

*Figure 59: ORTEP diagram of IMes-B(CN)$_2$Me, **9**. Ellipsoids are drawn at 50% probability levels. Hydrogen atoms are omitted for clarity. Selected bond lengths [Å] and angles [°]: B1-C1 1.627(2), B1-C22 1.604(2), B1-C23 1.594(2), B1-C24 1.628(2), C22-B1-C1 112.38(11), C23-B1-C1 112.55(12), C24-B1-C1 109.96(12). The molecule displays approximate mirror symmetry (r.m.s. deviation 0.05 Å).*

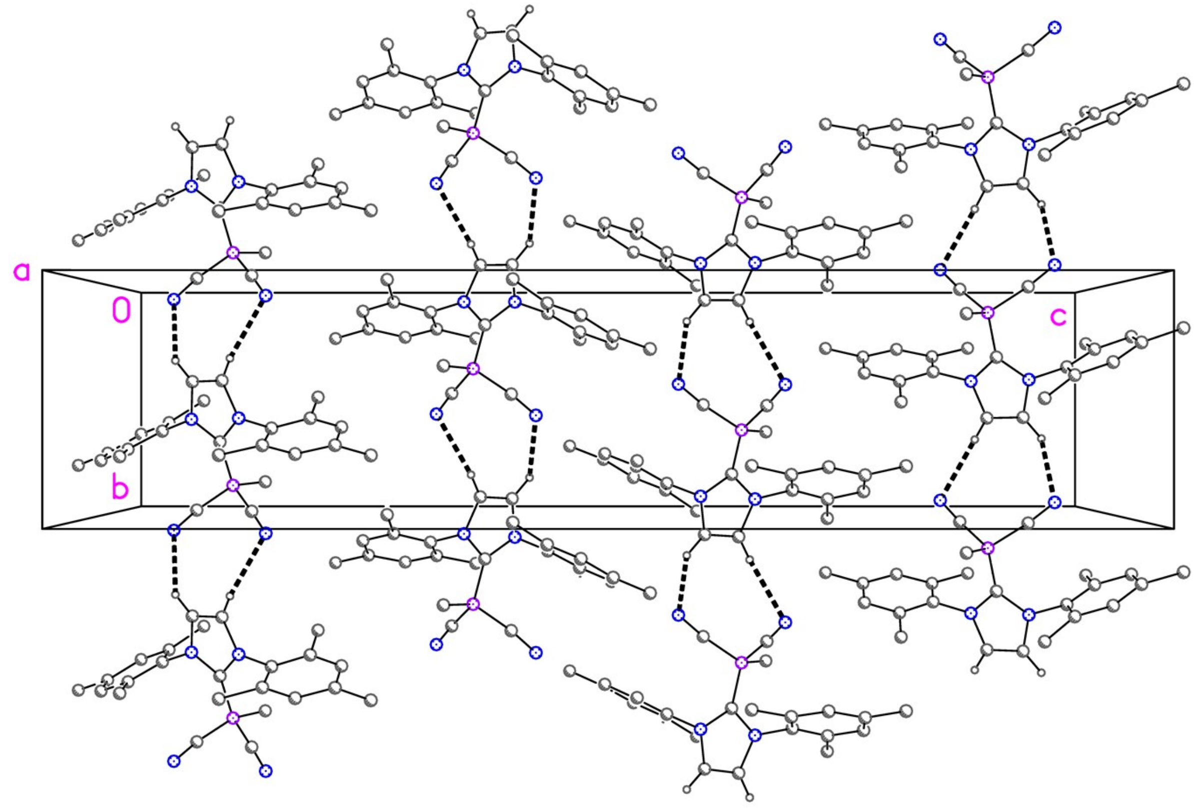

*Figure 60: Packing diagram of IMes-B(CN)$_2$Me, **9**. The molecules form chains parallel to the b axis via the contacts H2□N3 2.42 and H3□N4 2.42 Å.*

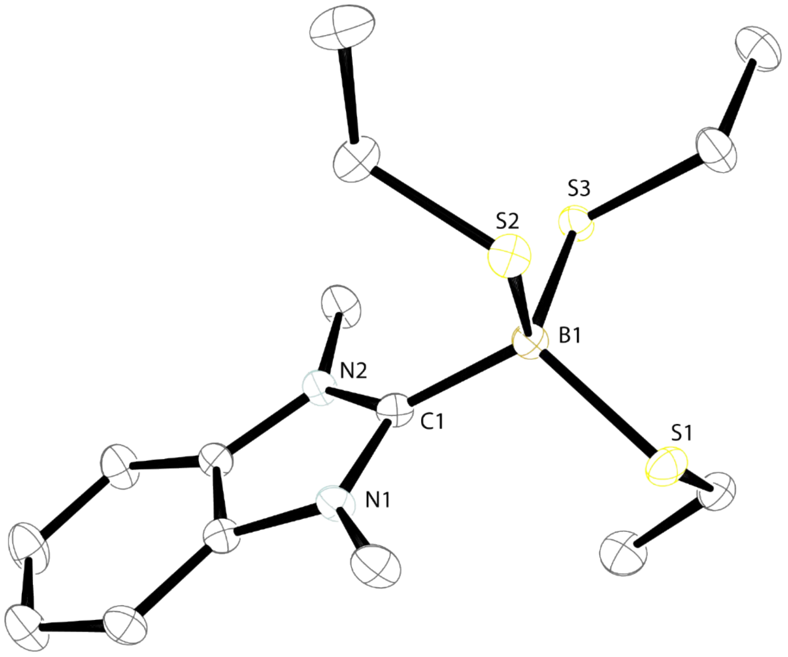

*Figure 61: ORTEP diagram of BAC-B(SEt)$_3$, **1C**. Ellipsoids are drawn at 50% probability levels. Hydrogen atoms are omitted for clarity. Selected bond lengths [Å] and angles [°]: S1-B1 1.9324(12), S2-B1 1.9217(12), S3-B1 1.9169(12), B1-C1 1.6419(15), N1-C1 1.3604(13), N2-C1 1.3613(13), C1-B1-S1 111.27(7), C1-B1-S2 106.97(7), C1-B1-S3 112.47(7).*

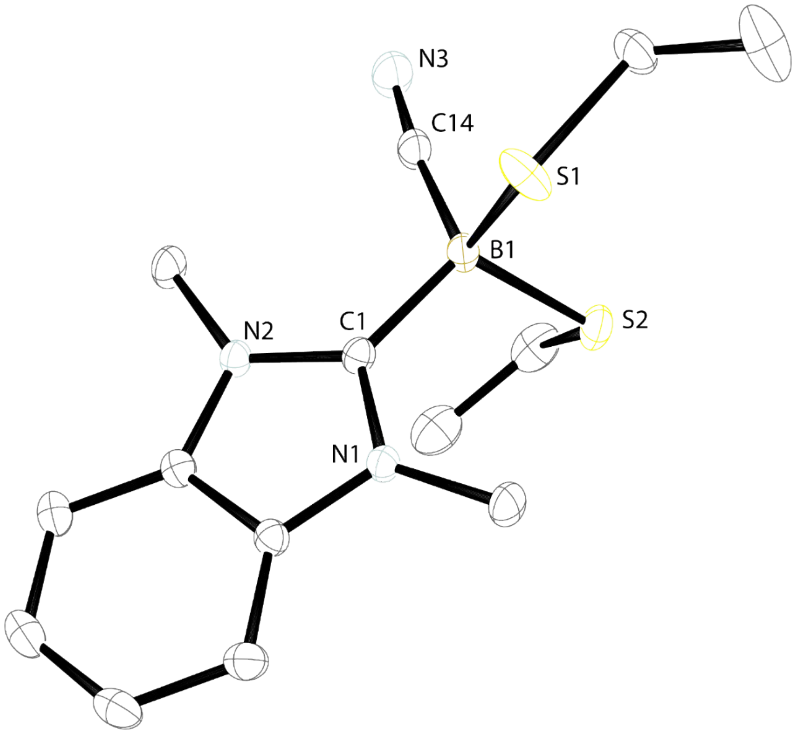

*Figure 62: ORTEP diagram of BAC-B(SEt)$_2$CN, **2C**. Ellipsoids are drawn at 50% probability levels. Hydrogen atoms are omitted for clarity. Selected bond lengths [Å] and angles [°]: S1-B1 1.9084(13), S2-B1 1.9250(12), B1-C14 1.5895(16), B1-C1 1.6229(16), N1-C1 1.3562(13), N2-C1 1.3558(13), C1-B1-S1 105.12(7), C1-B1-S2 109.70(6), C1-B1-C14 115.08(9).*

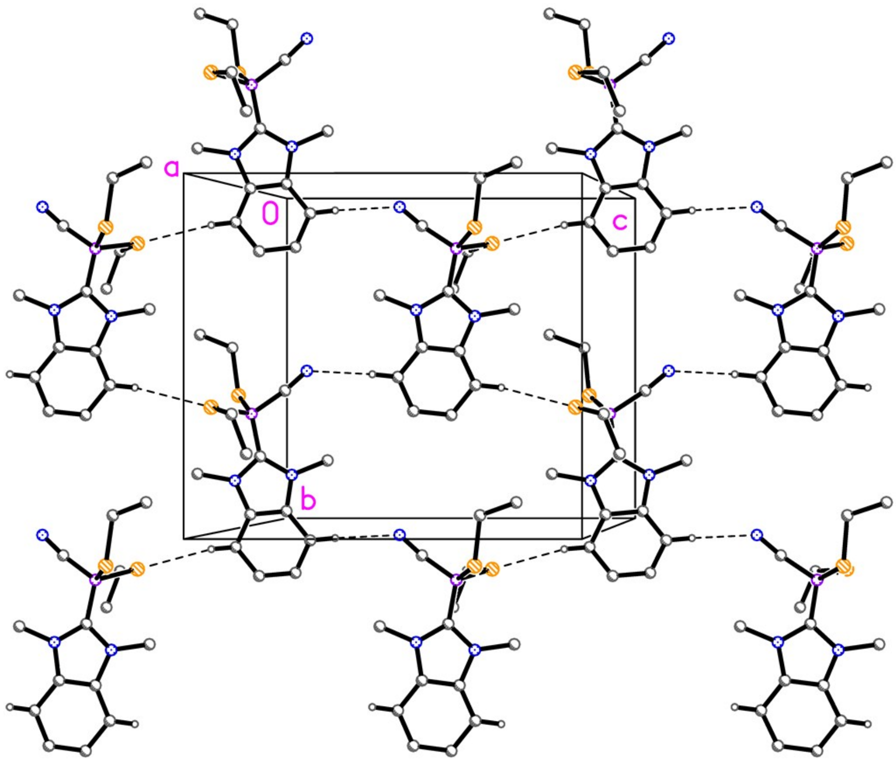

*Figure 63: Packing diagram of BAC-B(SEt)$_2$CN, **2C**. The molecules are linked to form layers parallel to the bc plane by the contacts H6□N3 2.39 and H3□S2 2.82 Å.*

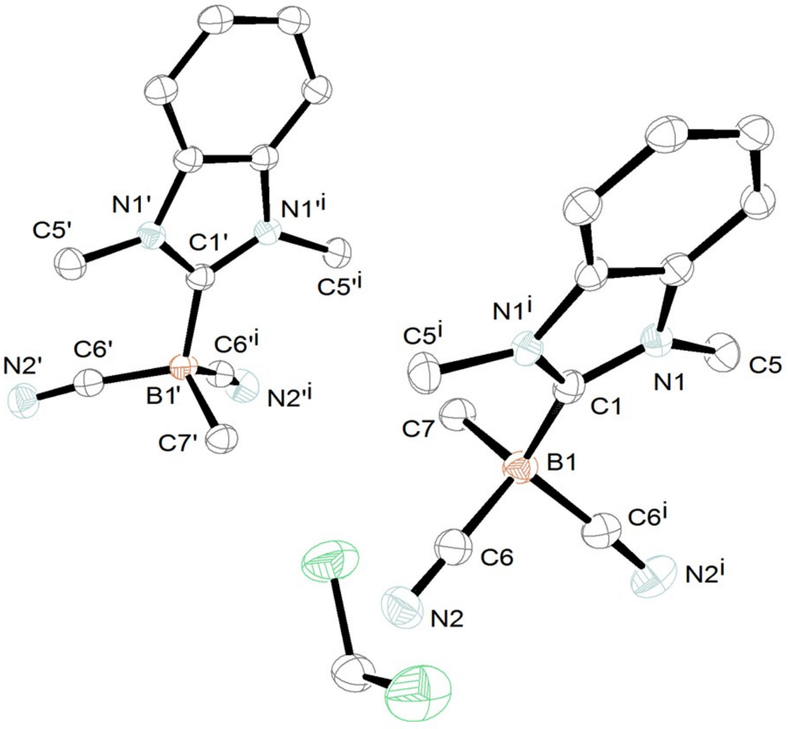

*Figure 64: ORTEP diagram of BAC-B(CN)$_2$Me, **11**. Ellipsoids are drawn at 50% probability levels. Hydrogen atoms are omitted for clarity. Selected bond lengths [Å] and angles [°]: B1-C1 1.638(3), B1-C6 1.603(2), B1-C7 1.624(2), C6-B1-C1 111.22(13), C7-B1-C1 111.22(13), C6-B1-C6^{i} 103.84(18), B1′-C1′ 1.625(3), B1-C6 1.609(2), B1′-C7′ 1.629(2), C6′-B1′-C1′ 111.88(13), C7′-B1′-C1′ 109.86(19), C6′-B1′-C6′^{i} 102.55(18)*

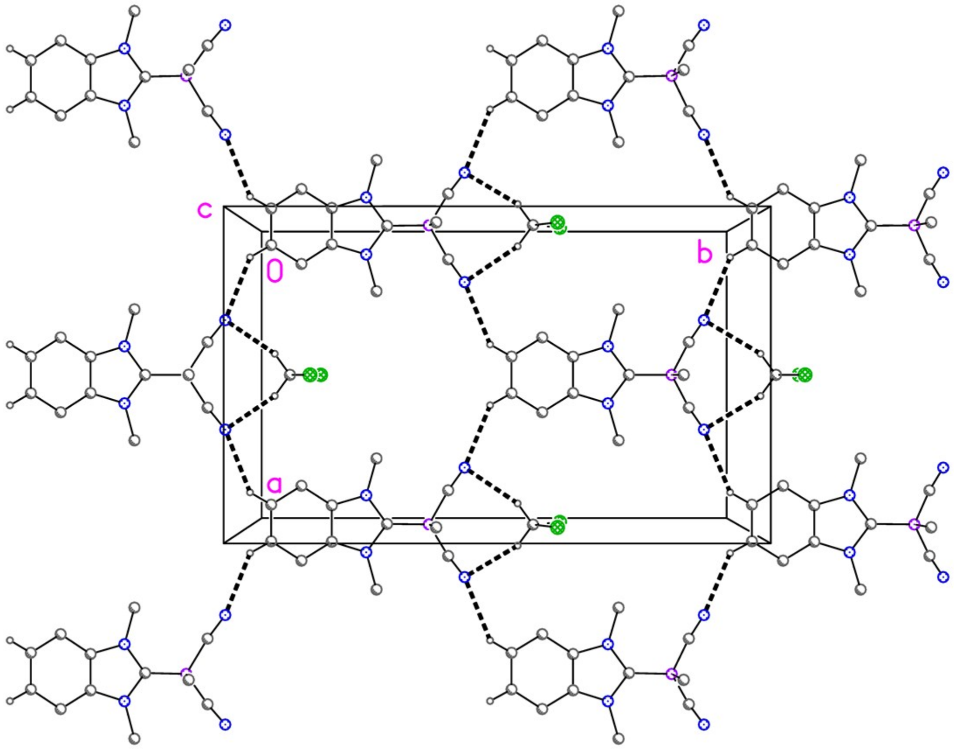

*Figure 65: Packing diagram of BAC-B(CN)$_2$Me, **11**. The second independent molecule of **11** (atom names with primes in the coordinate lists) together with the dichloromethane, forms a layer parallel to the ab plane. The contacts are H99(solvent)□N2' 2.70 and H4'□N2' 2.61 Å. The first molecule forms a similar plane but without direct contacts to dichloromethane.*

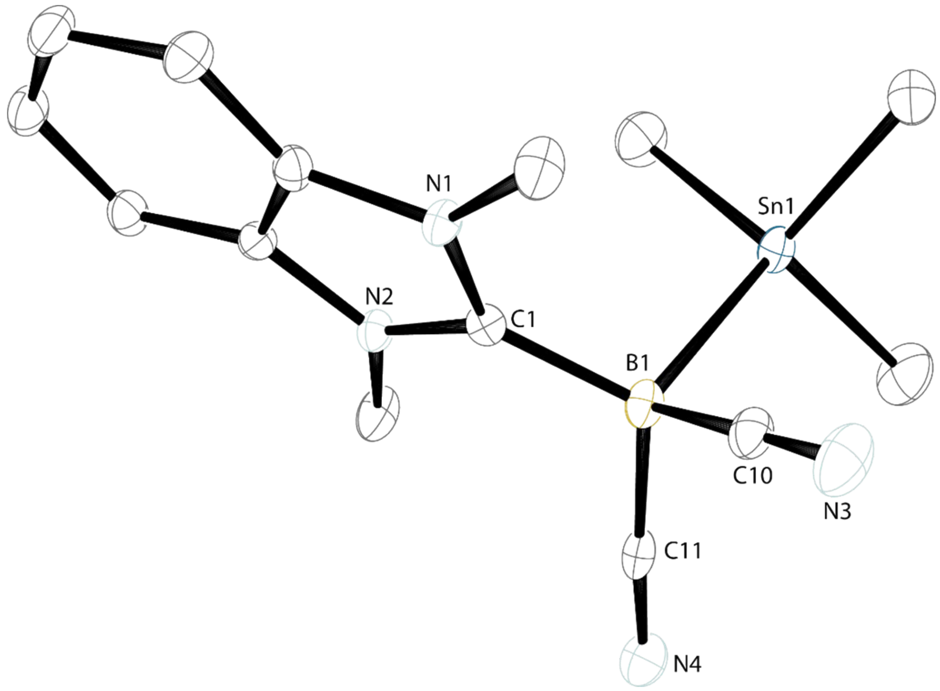

*Figure 66: ORTEP diagram of BAC-B(CN)$_2$SnMe$_3$, **13**. Ellipsoids are drawn at 50% probability levels. Hydrogen atoms are omitted for clarity. Selected bond lengths [Å] and angles [°]: Sn1-B1 2.324(2), B1-C1 1.586(3), B1-C10 1.572(3), B1-C11 1.573(3), Sn1-B1-C1 106.56(13), C10-B1-C1 116.03(17), C11-B1-C1 115.64(16). This is one of two independent molecules; a least-squares fit of both molecules gave an r.m.s. deviation of 0.11 Å.*

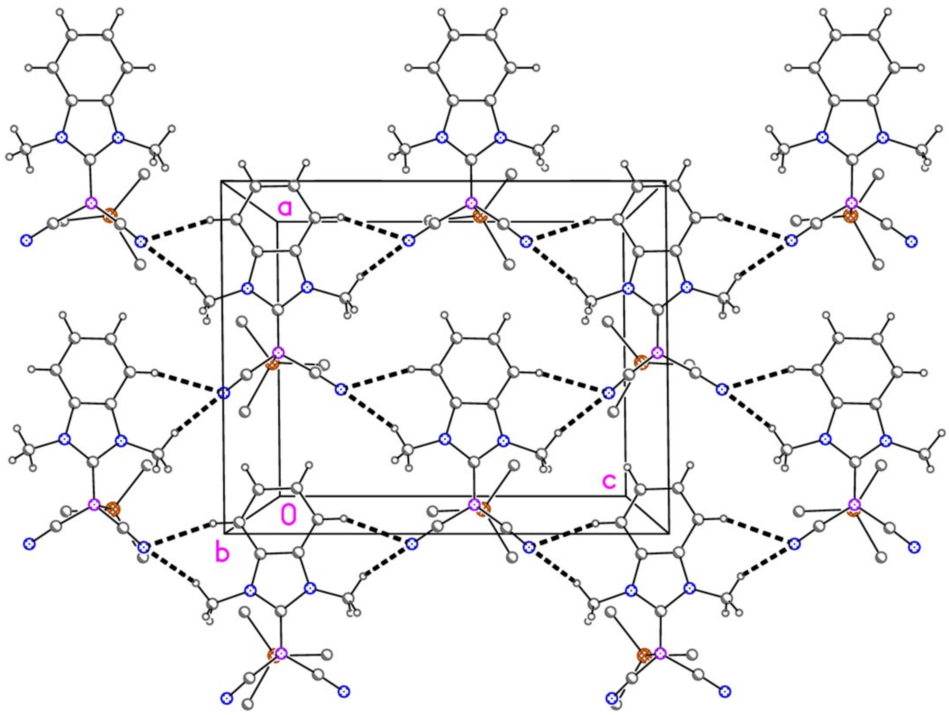

*Figure 67: Packing diagram of BAC-B(CN)$_2$SnMe$_3$, **13**. The molecules are linked by several H□N contacts of 2.35 – 2.73 Å to form a layer structure parallel to the ac plane. However, there are further links in the third dimension between these layers.*

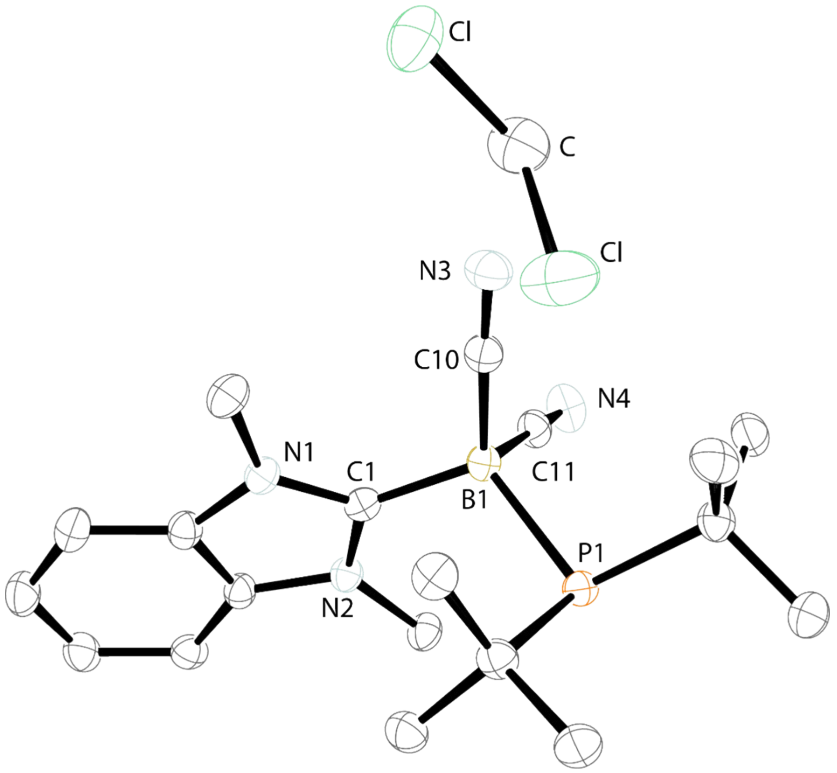

*Figure 68: ORTEP diagram of BAC-B(CN)$_2$P(tBu)$_2$, **14**. Ellipsoids are drawn at 50% probability levels. Hydrogen atoms are omitted for clarity. Selected bond lengths [Å] and angles [°]: B1-C10 1.586(2), B1-C11 1.601(2), B1-C1 1.628(2), B1-P1 2.0408(19), C10-B1-C1 113.02(14), C11-B1-C1 104.81(13), C1-B1-P1 111.72(11).*

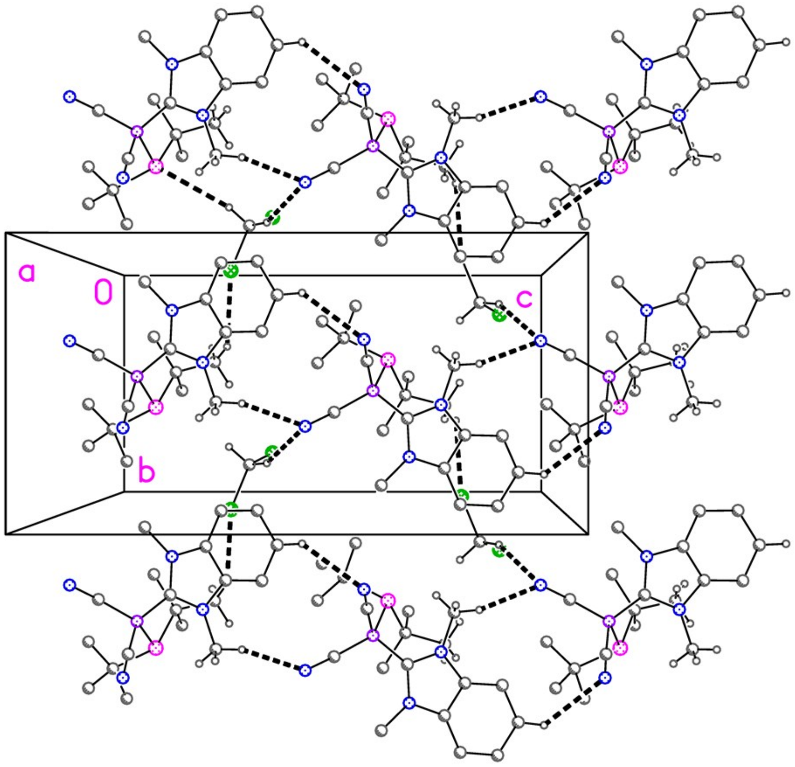

*Figure 69: Packing diagram of BAC-B(CN)$_2$P(tBu)$_2$, **14**. The molecules are linked by several H□N and H□Cl contacts to form a layer structure parallel to the bc plane. However, there are further links in the third dimension between these layers. A P□H(–Cl) contact of 3.02 Å is omitted.*

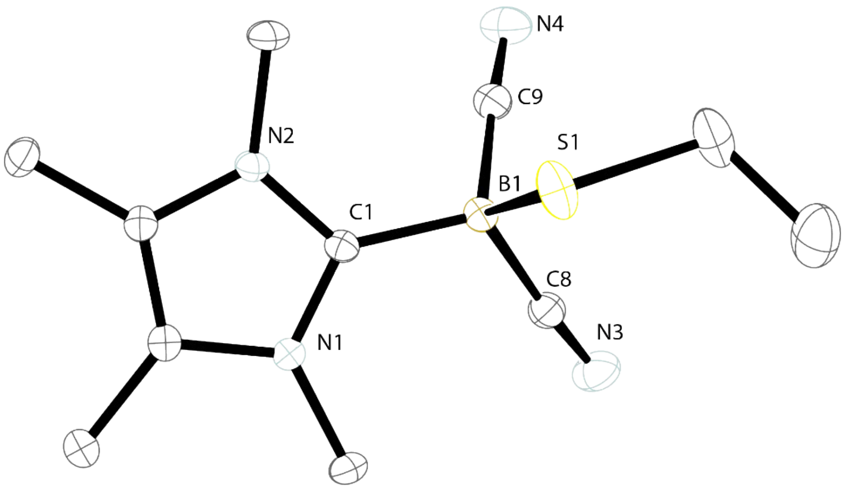

*Figure 70: ORTEP diagram of IMe$_2$(Me)$_2$-B(CN)$_2$SEt, **3A**. Ellipsoids are drawn at 50% probability levels. Hydrogen atoms are omitted for clarity. Selected bond lengths [Å] and angles [°]: S1-B1 1.8250(17), B1-C8 1.597(2), B1-C9 1.600(2), B1-C1 1.611(2), N1-C1 1.3475(17), N2-C1 1.3501(17), C8-B1-C1 113.28(12), C9-B1-C1 111.16(12), C1-B1-S1 106.12(9). The molecule possesses non-crystallographic mirror symmetry with an r.m.s. deviation of 0.20 Å.*

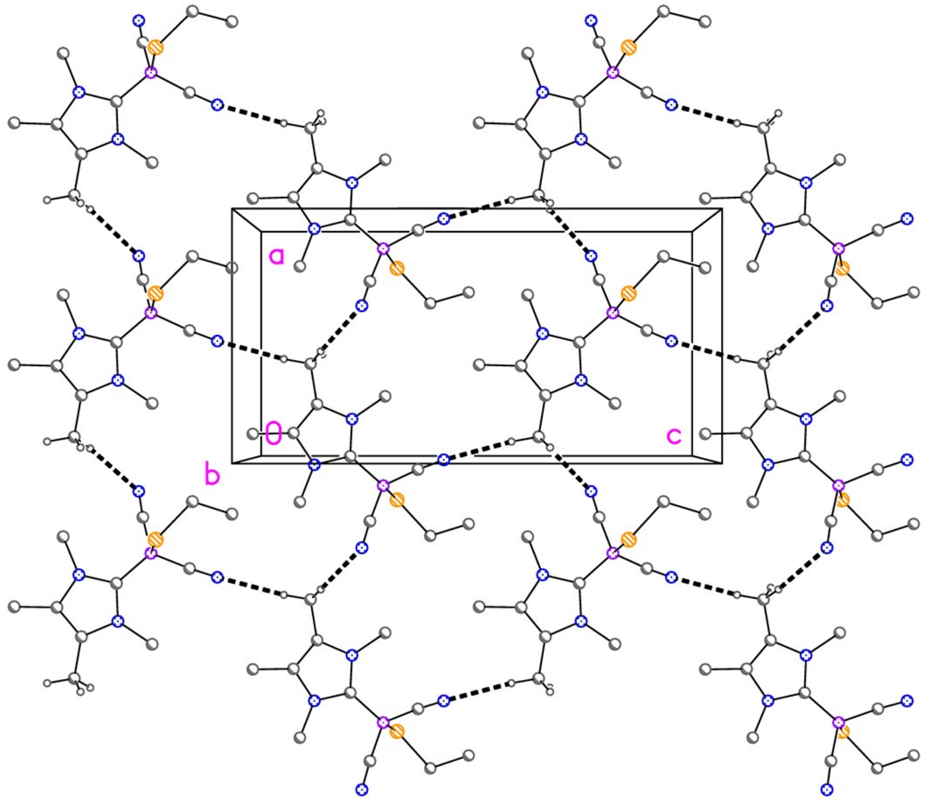

*Figure 71: Packing diagram of $IMe_2(Me)_2$-$B(CN)_2SEt$, **3A**. The molecules are linked to form layers parallel to the ac plane by the contacts H5A□N3 2.49 and H5C□N4 2.52 Å.*

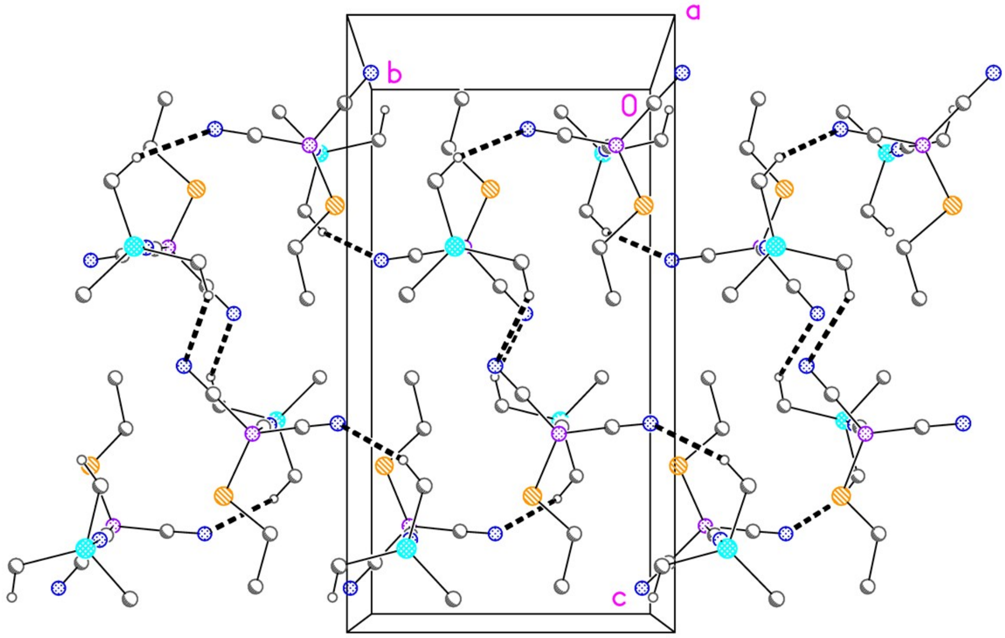

*Figure 72: Packing diagram of Me_3Si-N≡C-$B(CN)_2SEt$, **4**. The molecules are linked to form thick bands parallel to the bc plane by the contacts H2A□N3 2.66 and H3A□N2 2.55 Å.*

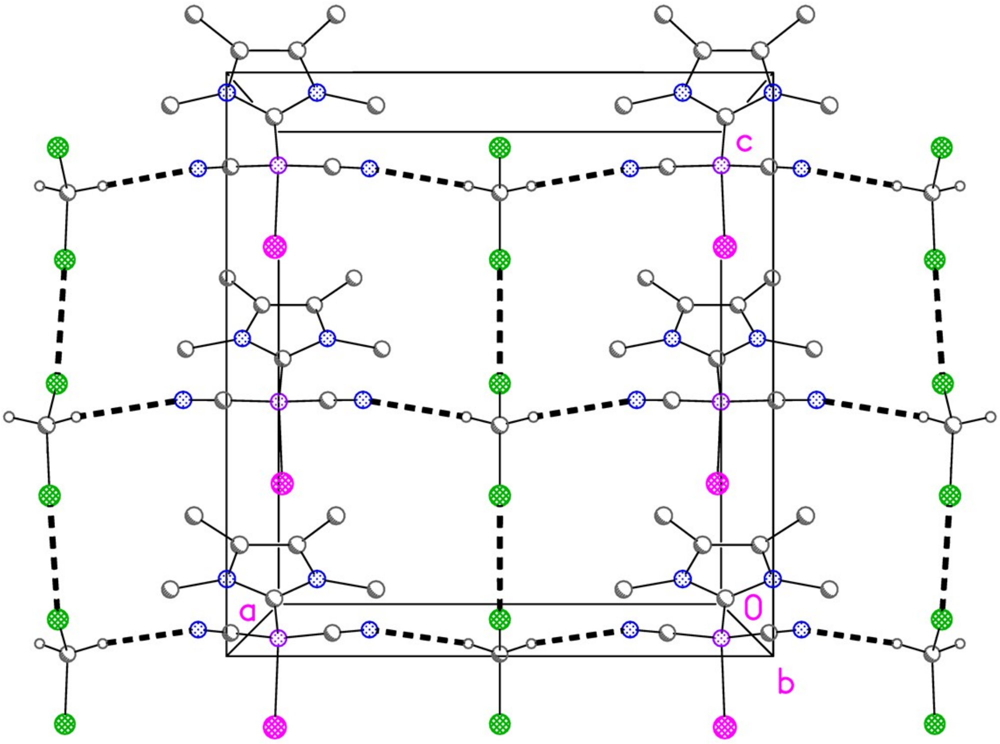

*Figure 73: Packing diagram of IMe(Me)$_2$-B(CN)$_2$I, **7A** (dichloromethane solvate). The molecules are linked to form corrugated layers parallel to the ac plane by the contacts H99A□N2 2.57 and Cl1□Cl2 3.33 Å.*

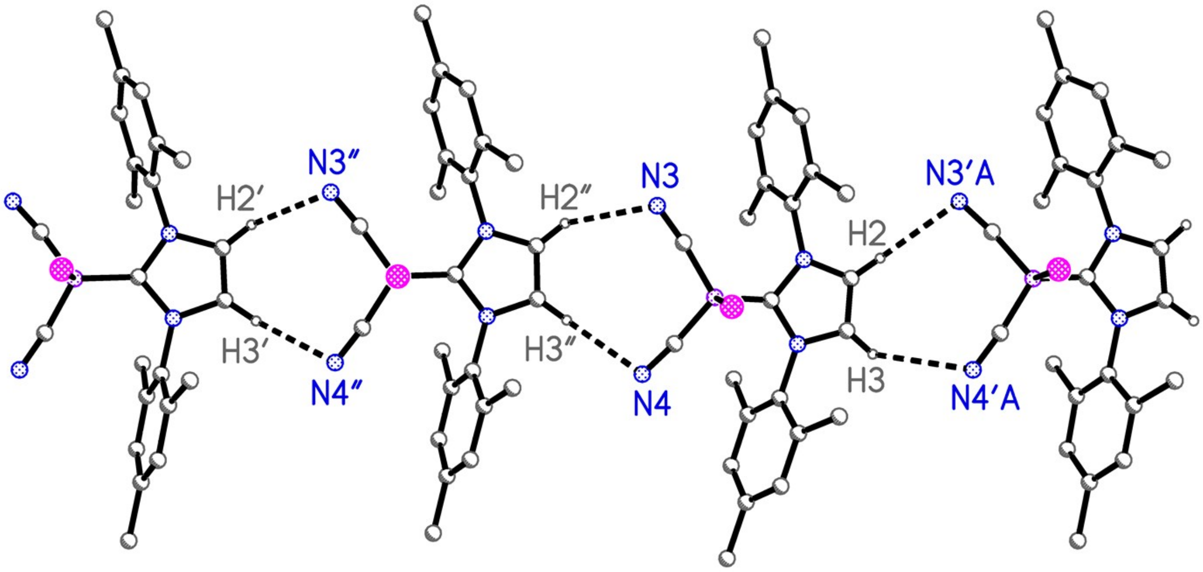

*Figure 74: The three independent molecules of IMes-B(CN)$_2$I, **7B**, together with one further symmetry-generated molecule (extreme right). The molecules are form a chain parallel to the c axis by the contacts H2'□N3" 2.56, H3'□N4" 2.50, H2□N3'A 2.50, H3□N4'A 2.71, H2"□N3 2.6356, H3"□N4 2.51 Å.*

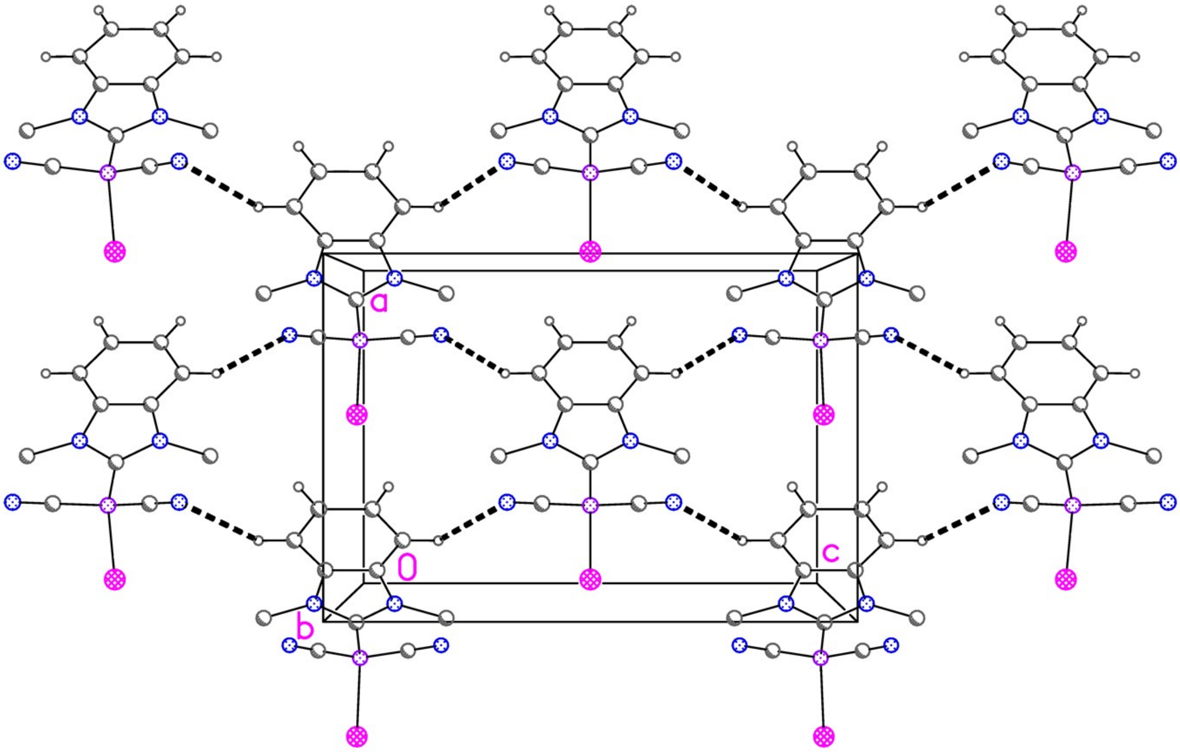

*Figure 75: Packing diagram of BAC-B(CN)$_2$I, **7C**. The molecules form corrugated chains parallel to the ac plane by the contacts H3□N2 2.60 Å. The layers are linked in the third dimension by the contact H5C(methyl) □N2 2.57 Å (not shown here).*

DFT Calculations

All DFT calculations were performed using ORCA2.9.1.[7] TZVP basis sets[8] were used throughout. Geometric structure optimizations of several boryl anions (see Figure 76) were performed with the GGA functional BP86.[9] Dispersion contributions were approximated using Grimme's DFT-D3 atom-pair-wise dispersion corrections.[10] Solvent effects were taken into account at the polarizable continuum model level, using COSMO (Conductor-Like Screening Model)[11] implemented in ORCA, with parameters for tetrahydrofuran. Numerical frequency calculations revealed the optimized structures of **8B** and **8C** to be stationary points on the energy surface. xyz-Data of the optimized structures are given in Tables 5−9. UV/VIS/NIR spectroscopic properties of **8B** were extracted from single-point TD-DFT calculations at the BLYP-35[12]-D3/TZVP/COSMO(THF) level of theory in the BP86-optimized atomic positions. Spectral positions and intensities of the computed transitions are given in Table 4.

*Table 4: Computed UV-Vis transitions of anion IMes-B(CN)$_2^-$ (**8B**); transitions with vanishing oscillator strength (f_{osc} < 10^{-5}) are neglected.*

	$\tilde{\nu}$ [cm^{-1}]	λ [nm]	f_{osc}
2	19305.5	518	0.00523
5	20924.2	477.9	0.02796
8	21625.6	462.4	9.61838E-4
9	21953.8	455.5	9.2088E-5
12	28257.3	353.9	0.01404
16	27855.9	359	0.03597
17	30487.1	328	0.04296
19	33875.7	295.2	0.50733
23	34271.5	291.8	7.8436E-5
28	35374.6	282.7	4.4663E-5
30	36844.9	271.4	1.0188E-4
31	37317.1	268	0.00255
33	35213.8	284	0.00237
38	38590.9	259.1	0.00464
39	38667.1	258.6	0.0046
40	41347.5	241.9	0.23018

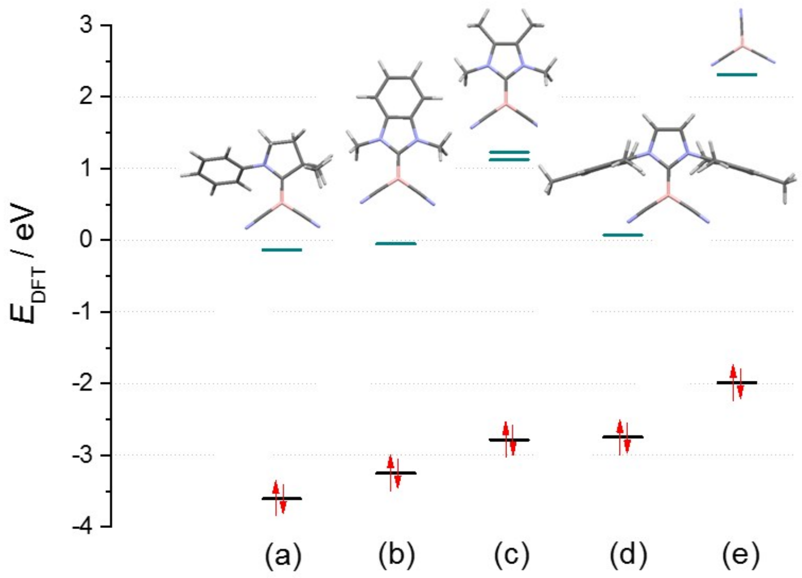

	(a)	(b)	(c)	(d)	(e)
**Bond-Order** (BC) =	1.59	1.46	1.35	1.43	1.20
**Mulliken-Charge** (B) =	−0.14	−0.22	−0.33	−0.39	−0.57

*Figure 76. Energies of frontier orbitals in the boryl anion series L–B(CN)$_2$$^-$, L = carbene or CN$^-$: (a) truncated anion **VI**; (b) **8C**; (c) hypothetical **8A**; (d) **8B**; (e) anion **VII**.*

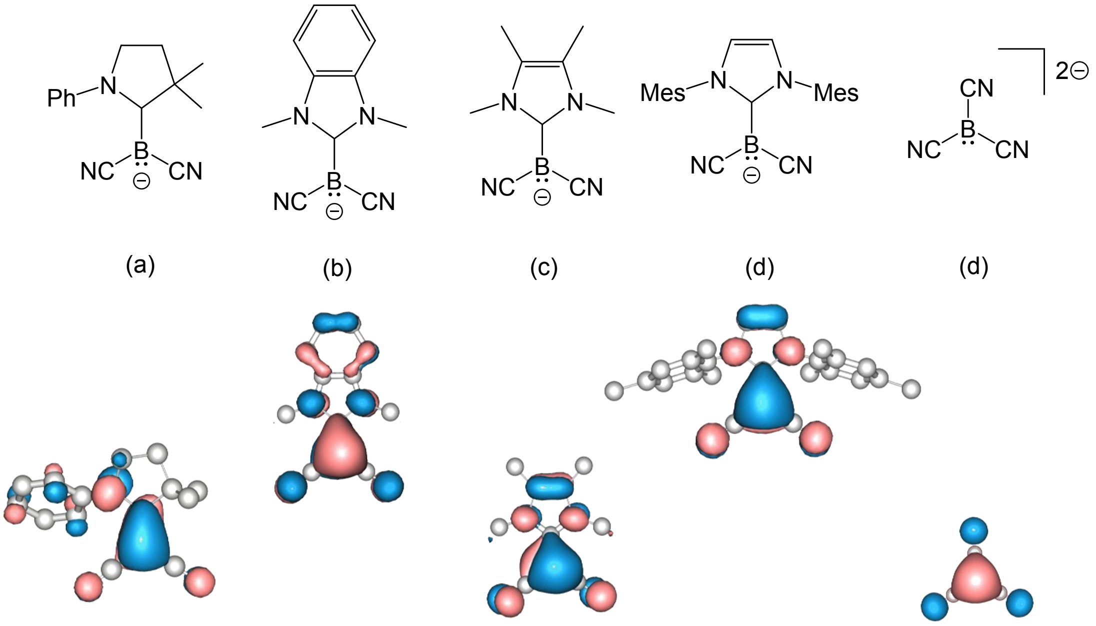

*Figure 77. HOMOs within the boryl anion series L–B(CN)$_2$$^-$, L = carbene or CN$^-$: (a) truncated anion **VI**; (b) **8C**; (c) hypothetical **8A**; (d) **8B**; (e) anion **VII**.*

*Table 5: Coordinates of boryl anion **VI**.*

B	4.733395	14.944204	11.189277
C	5.907152	15.486530	13.539550
N	4.001091	14.077897	13.511682
N	6.393406	16.533066	9.748827
N	3.156310	13.420185	9.600004
C	4.804623	14.852776	12.663406
C	5.502744	15.078507	14.981678
H	4.871526	15.862684	15.428299
C	4.672009	13.813134	14.795165
H	3.926399	13.657453	15.585885
C	5.957212	17.017195	13.412605
C	2.707685	13.602312	13.282523
C	1.789048	14.324148	12.490218
C	0.481630	13.873172	12.337068
H	-0.211923	14.455210	11.726271
C	0.047829	12.691323	12.957215
C	0.951726	11.972121	13.746708
H	0.637877	11.046212	14.233344
C	2.265977	12.416994	13.912647
C	5.687468	15.847853	10.394212
C	3.819371	14.085836	10.306115
H	-0.976059	12.338714	12.823978
H	2.961012	11.833489	14.517822
H	2.116628	15.244008	12.008687
H	6.671808	17.433841	14.141363
H	4.967263	17.453668	13.611468
H	6.273206	17.327058	12.408410
H	5.306329	12.910178	14.732948
H	6.376516	14.927106	15.630394
C	7.283330	14.883268	13.189886
H	8.065345	15.315645	13.834602
H	7.281830	13.791662	13.329656
H	7.541319	15.088880	12.142491

*Table 6: Coordinates of boryl anion **8C**.*

C	3.766663	15.250594	6.945704
C	4.558200	15.976901	8.980468
C	4.727342	16.466581	10.275755
H	3.877348	16.783249	10.880478
C	6.041175	16.536342	10.782507
H	6.200516	16.917622	11.792137
C	7.136326	16.118965	10.017357
H	8.143066	16.182926	10.433865
C	6.967356	15.622172	8.708749
H	7.826197	15.311803	8.113533
C	5.667581	15.550121	8.206672
C	2.103003	16.256191	8.559121
H	1.446921	15.423094	8.848960
H	1.650463	16.774707	7.703343
H	2.188146	16.956628	9.398872
C	5.956097	14.450472	5.971882
H	6.920378	14.159641	6.403888
H	6.131038	15.104500	5.105940
H	5.430866	13.549825	5.628329
C	1.326303	14.830665	5.983027
C	3.343597	14.578246	4.409335
B	2.844484	14.898707	5.818133
N	3.429861	15.796528	8.196883
N	5.164735	15.110221	6.993024
N	0.153796	14.731729	6.062776
N	3.669890	14.353354	3.298585

Table 7: Coordinates of boryl anion ***8A***.

B	4.767290	14.835205	11.342291
N	5.763524	15.447061	13.683326
N	3.882892	14.323618	13.750863
N	6.197881	16.538634	9.800083
N	3.269140	13.070980	9.939050
C	4.803265	14.867383	12.858851
C	5.441821	15.262025	15.038315
C	6.300788	15.769458	16.143699
C	4.268203	14.563603	15.080334
C	3.462179	14.102964	16.244508
C	7.014756	15.987746	13.187858
C	2.610397	13.764149	13.337781
C	5.593133	15.792220	10.490943
C	3.903930	13.844286	10.570846
H	2.149666	14.427207	12.589011
H	1.950158	13.681244	14.208073
H	2.730424	12.770766	12.883207
H	6.871663	16.960955	12.697367
H	7.441261	15.293640	12.446926
H	7.714857	16.108044	14.021856
H	7.303844	15.312946	16.137209
H	5.839563	15.534692	17.111382
H	6.435884	16.861966	16.091640
H	3.326143	13.009394	16.243595
H	2.459237	14.559301	16.265357
H	3.967059	14.377104	17.179379

*Table 8: Coordinates of boryl anion **8B**.*

B	4.761874	14.877285	11.340643
N	5.783265	15.414671	13.686436
N	3.877692	14.328162	13.741134
N	6.689524	15.945234	9.773476
N	2.794280	13.734522	9.879805
C	4.800483	14.880851	12.841456
C	5.492810	15.146214	15.028306
H	6.142650	15.488032	15.824649
C	4.314854	14.474478	15.062290
H	3.725117	14.109443	15.894270
C	6.924888	16.146142	13.244264
C	6.774778	17.499480	12.896295
C	7.905711	18.194274	12.455198
H	7.802468	19.245042	12.170547
C	9.158059	17.572432	12.351569
C	9.268426	16.222659	12.712830
H	10.236284	15.721033	12.625437
C	8.161360	15.487495	13.154149
C	5.421208	18.151542	12.961232
H	5.485161	19.218347	12.710353
H	4.971069	18.048414	13.960599
H	4.734798	17.656769	12.253212
C	10.349937	18.331187	11.822198
H	11.293748	17.856443	12.124350
H	10.355156	19.371163	12.178797
H	10.332183	18.364085	10.720370
C	8.265549	14.020466	13.478285
H	8.095307	13.824812	14.548713
H	9.257257	13.631712	13.211965
H	7.502918	13.452233	12.925588
C	2.657296	13.690877	13.369553
C	1.549964	14.483421	13.021319
C	0.368247	13.834391	12.647008
H	-0.496232	14.437796	12.356587
C	0.274141	12.435894	12.613896
C	1.402089	11.679572	12.963054
H	1.351588	10.587711	12.916907
C	2.606282	12.287333	13.338567
C	1.665585	15.982036	13.010875
H	2.001320	16.366292	13.985924
H	0.705132	16.450490	12.759397
H	2.422360	16.292975	12.270986
C	-1.017335	11.759071	12.223484
H	-1.682490	11.642389	13.095313
H	-0.834047	10.755840	11.814014
H	-1.562818	12.345951	11.470573
C	3.835819	11.475354	13.650349
H	4.126046	11.564513	14.709109
H	4.690219	11.830812	13.055313
H	3.668593	10.412967	13.428604
C	5.872233	15.491333	10.495862
C	3.633449	14.217838	10.556544

*Table 9: Coordinates of boryl anion **VII**.*

B	4.737921	14.878634	11.285838
N	4.817272	14.923721	13.989730
N	6.256239	16.602101	9.856613
N	3.134897	13.111828	10.010245
C	4.782968	14.904206	12.802148
C	5.588878	15.845529	10.484502
C	3.839076	13.887742	10.570654

References

[1] A. Pelter, T. E. Levitt, K. Smith, A. Jones, *J. Chem. Soc.,* **1977**, *1*, 1672.
[2] M. T. Reetz, I. Chatziiosifidis, *Synthesis*, **1982**, 330.
[3] H.V. Huynh, J. H. H. Ho, T. C. Neo, L. L. Koh, *J. Organomet. Chem.* **2005**, *690*, 3854−3860.
[4] A. J. Arduengo, R. Krafczyk, R. Schmutzler, *Tetrahedron*, **1999**, *55*, 14523−14534.
[5] N. Kuhn, T. Kratz, *Synthesis* **1993**, 561−562.
[6] G. M. Sheldrick, *Acta Crystallogr., Sect. A. Crystallogr*. **2008**, *64*, 112–122.
[7] F. Neese, *WIREs Comput. Mol. Sci.* **2012,** *2* (1), 73−78.
[8] A. Schäfer, H. Horn, R. Ahlrichs, *J. Phys. Chem.* **1992,** *97* (), 2571−2577.
[9] A. D. Becke, *Phys. Rev. A* **1988,** *38* (6), 3098−3100.
[10] (a) S. Grimme, *J. Comput. Chem.* **2006,** *27* (15), 1787−1799; (b) S. Grimme, S. Ehrlich, L. Goerigk, *J. Comput. Chem.* **2011,** *32* (7), 1456−1465.
[11] A. Klamt, G. Schüürmann, *J. Chem. Soc. Perkin Trans. 2*, **1993**, *5*, 799.
[12] M. Parthey, M. Kaupp, *Chem. Soc. Rev.* **2014,** *43*, 5067−5088.
